# Supplementary figures and images for: Eugenol mimics exercise to promote skeletal muscle fiber remodeling and myokine IL-15 expression by activating TRPV1 channel (part 1 of 2)
Source: eLife. 2024 Jun 24;12:RP90724. doi: 10.7554/eLife.90724 (PMC11196110; doi:10.7554/eLife.90724)

Con

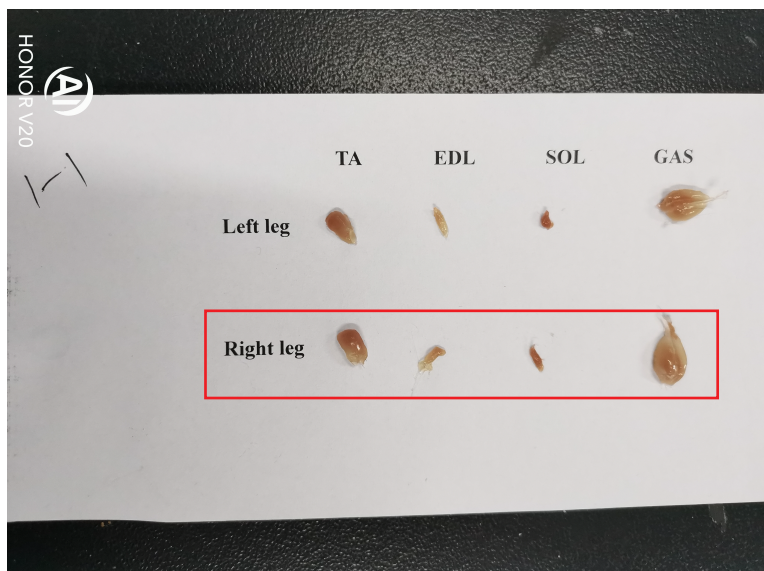

EUG50

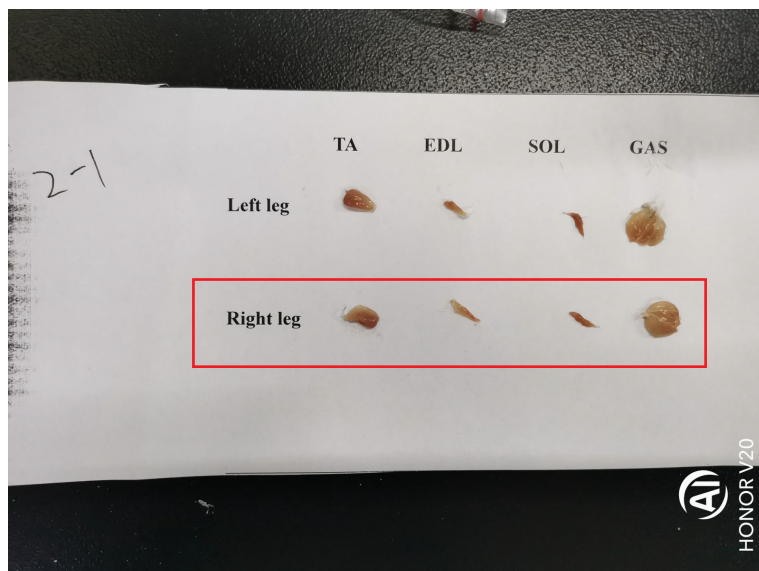

EUG100

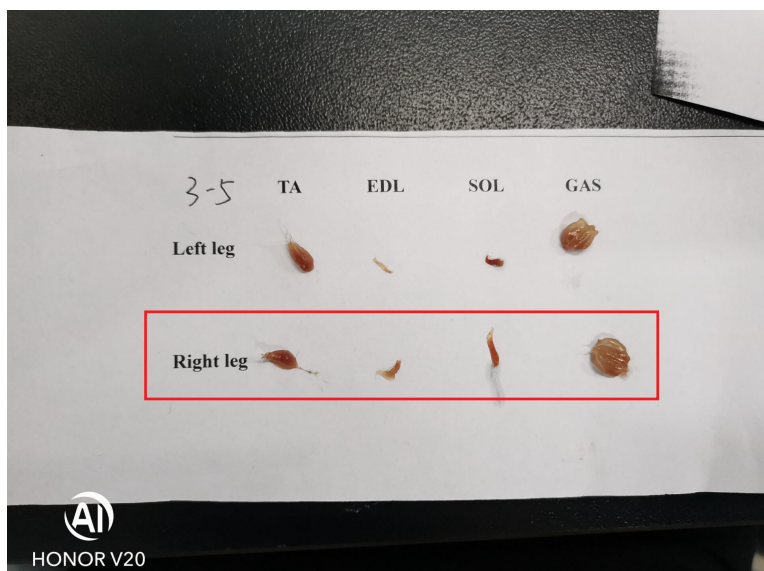

EUG200

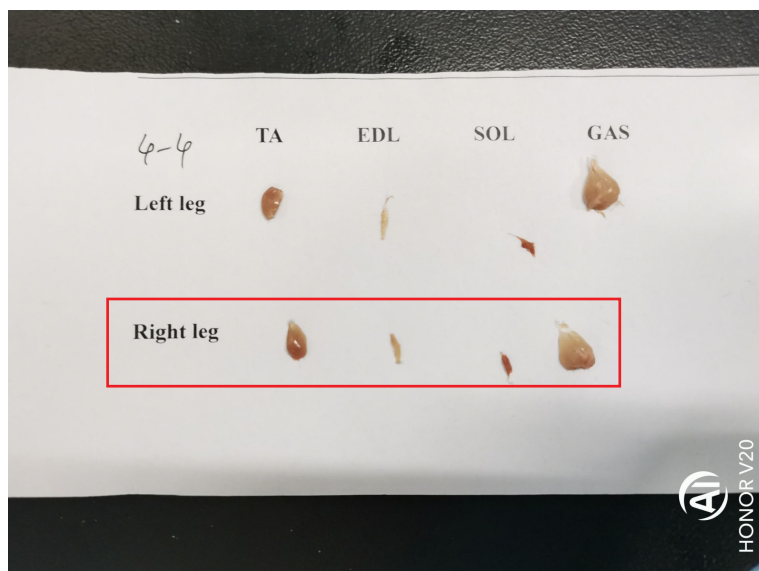

Supplement: Figure 1—source data 3. [file elife-90724-fig1-data3.pdf]

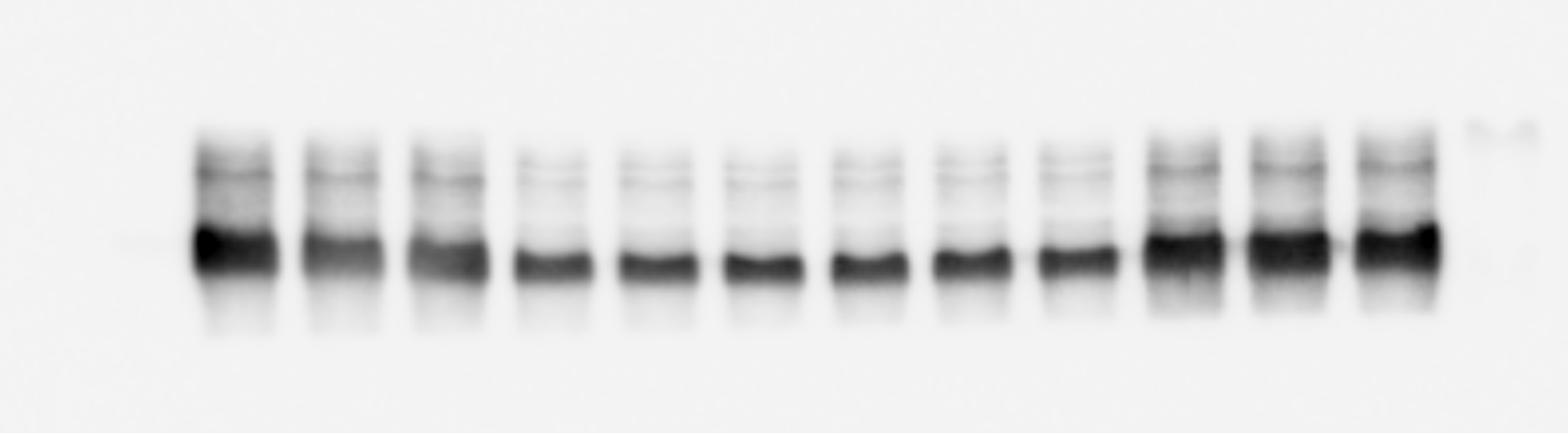

Supplement: Figure 1—source data 4. [file elife-90724-fig1-data4.zip › Fast MyHC.tif]

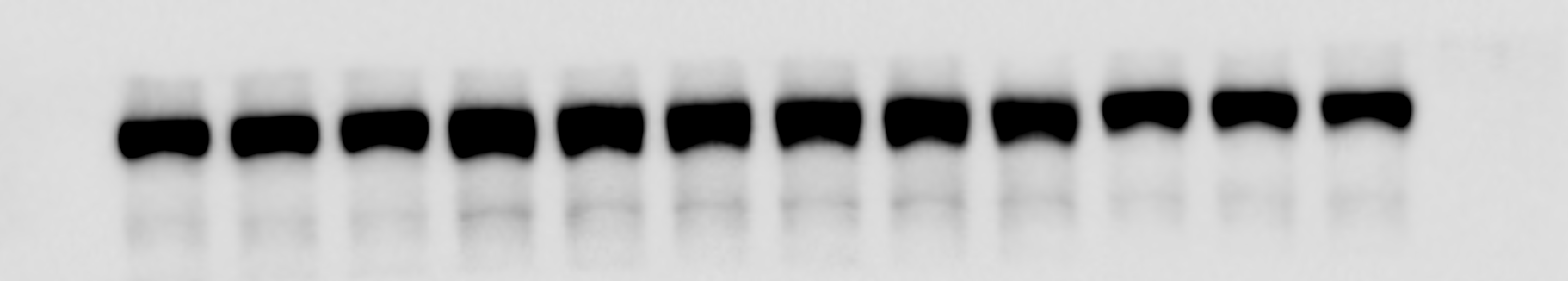

Supplement: Figure 1—source data 4. [file elife-90724-fig1-data4.zip › Slow MyHC.tif]

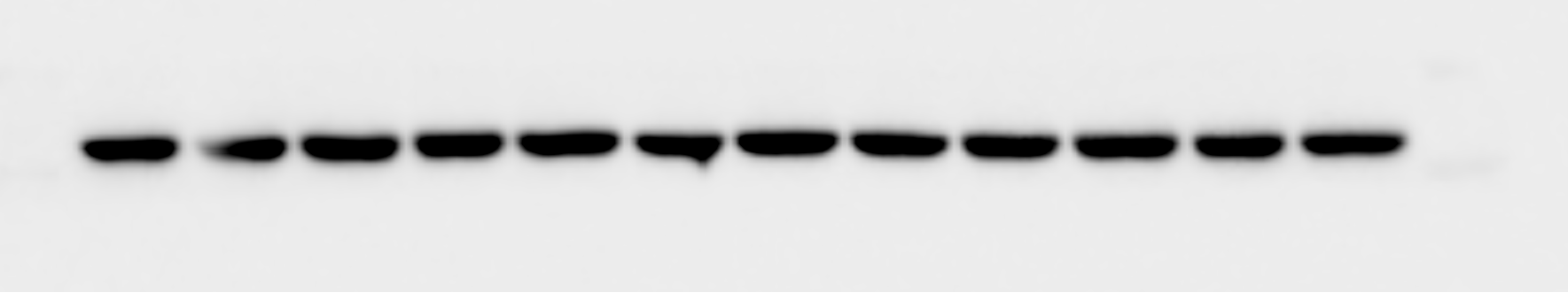

Supplement: Figure 1—source data 4. [file elife-90724-fig1-data4.zip › a┬-Actin.tif]

## Figure 1D

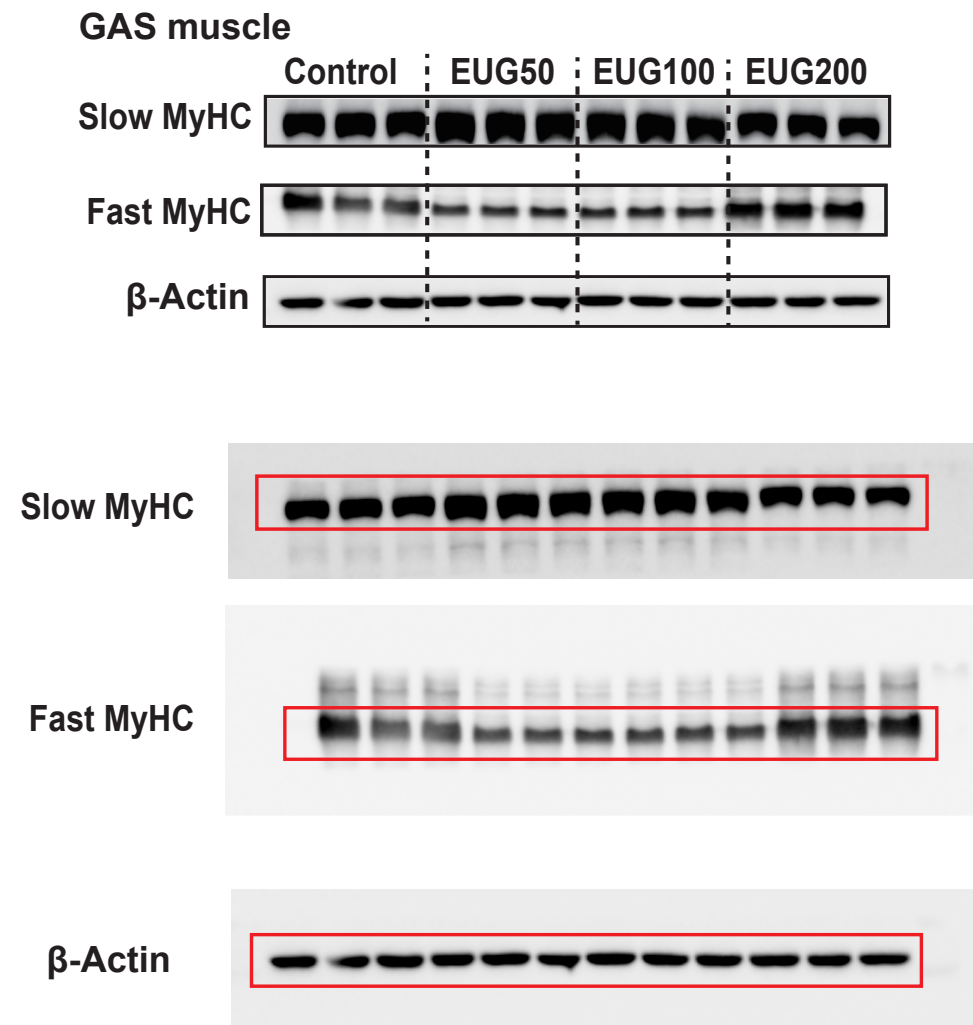

Supplement: Figure 1—source data 5. [file elife-90724-fig1-data5.pdf]

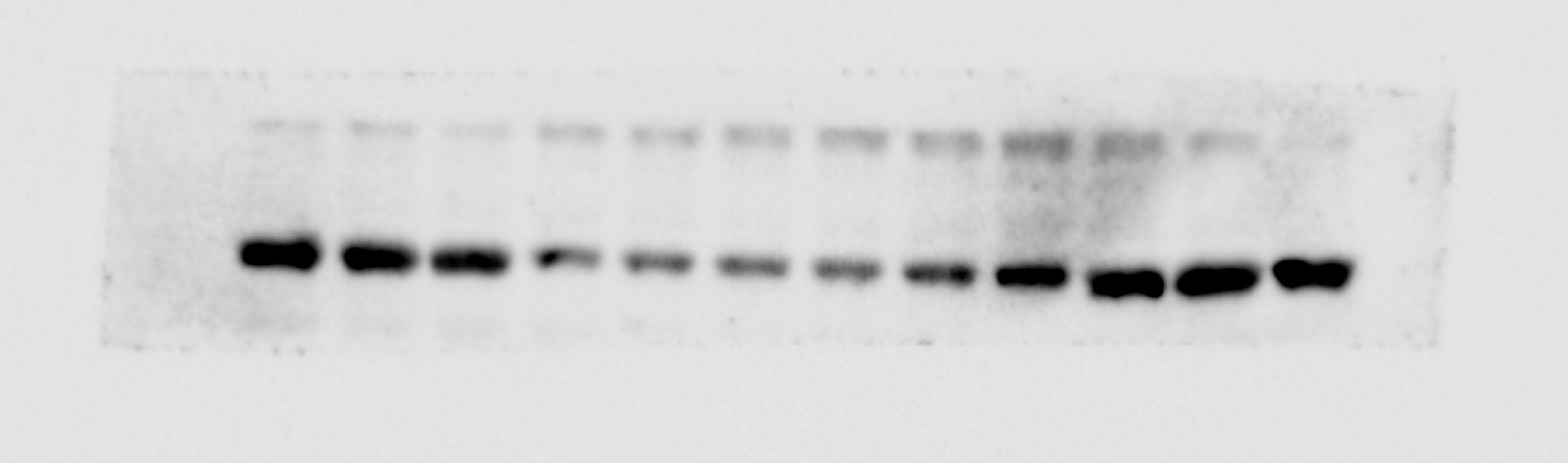

Supplement: Figure 1—source data 6. [file elife-90724-fig1-data6.zip › Fast MyHC.tif]

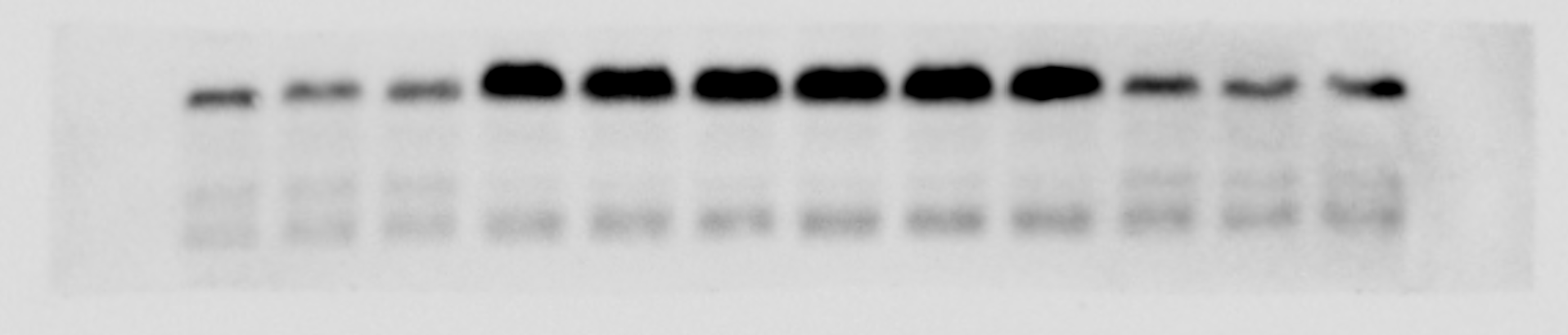

Supplement: Figure 1—source data 6. [file elife-90724-fig1-data6.zip › Slow MyHC.tif]

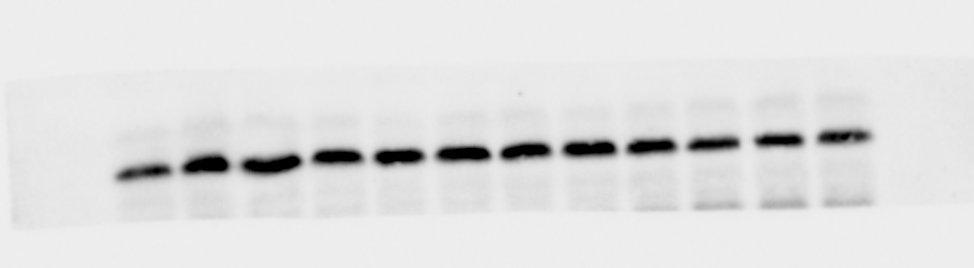

Supplement: Figure 1—source data 6. [file elife-90724-fig1-data6.zip › a┬-Actin.tif]

Figure 1E

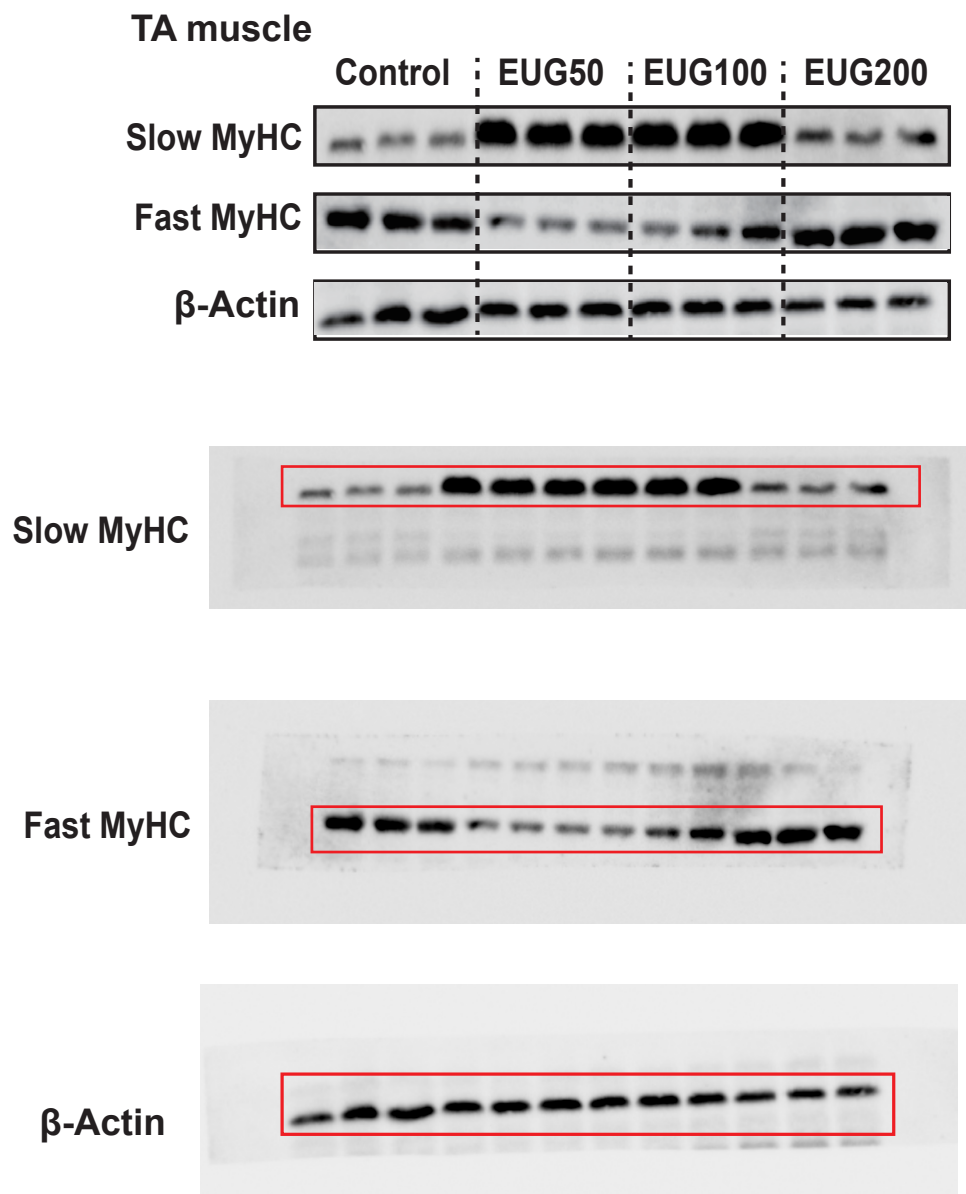

Supplement: Figure 1—source data 7. [file elife-90724-fig1-data7.pdf]

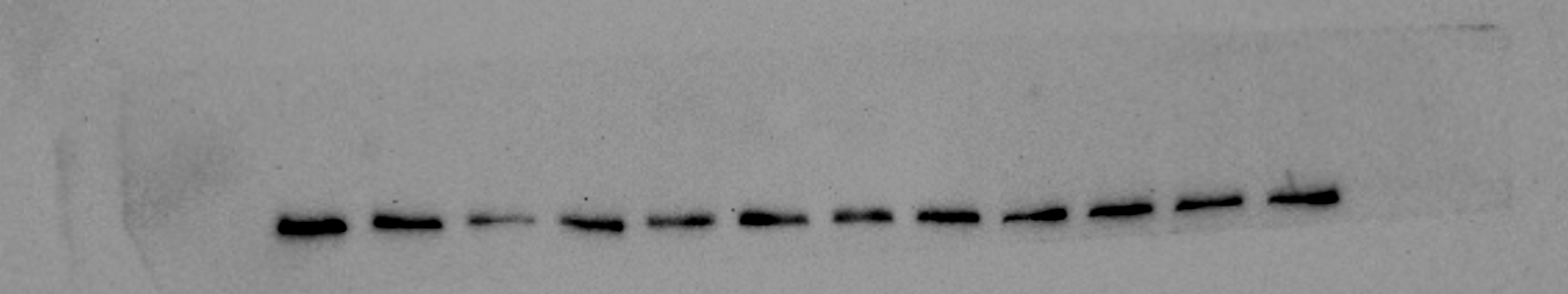

Supplement: Figure 1—source data 8. [file elife-90724-fig1-data8.zip › Fast MyHC.tif]

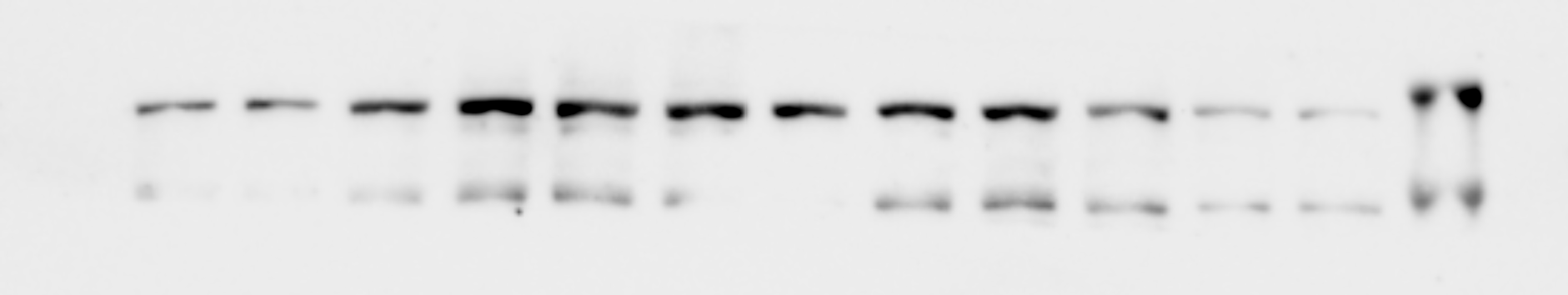

Supplement: Figure 1—source data 8. [file elife-90724-fig1-data8.zip › Slow MyHC.tif]

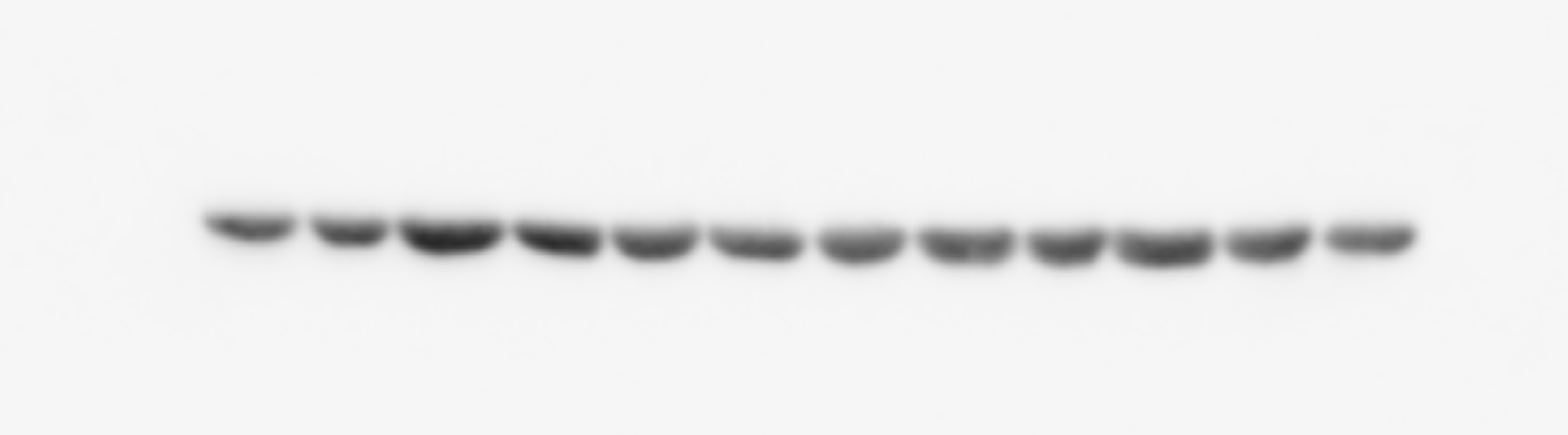

Supplement: Figure 1—source data 8. [file elife-90724-fig1-data8.zip › a┬-Actin.tif]

**Figure 1F**

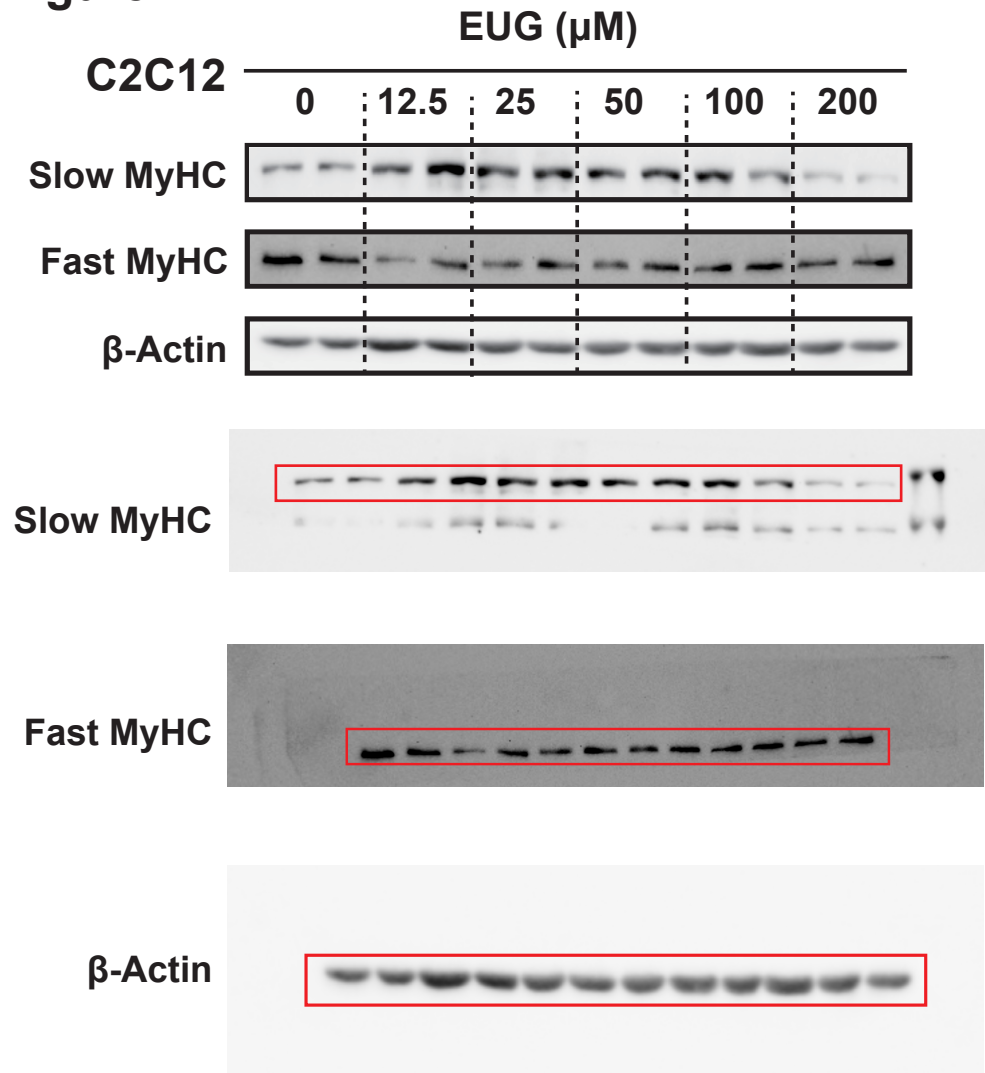

Supplement: Figure 1—source data 9. [file elife-90724-fig1-data9.pdf]

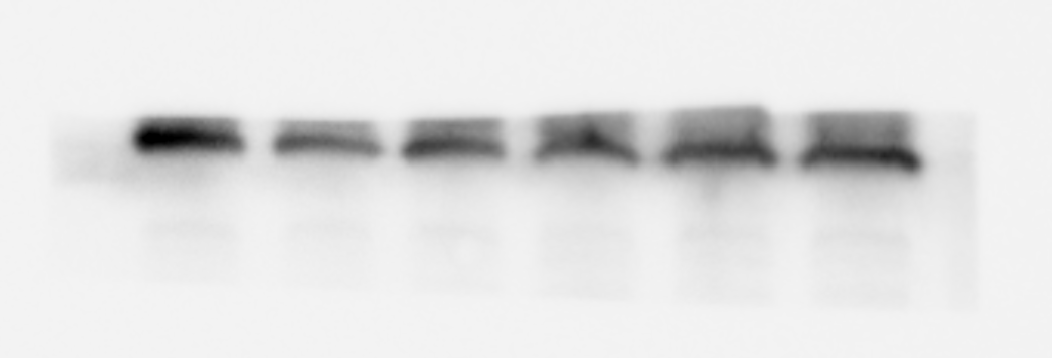

Supplement: Figure 2—source data 4. [file elife-90724-fig2-data4.zip › Complex I.tif]

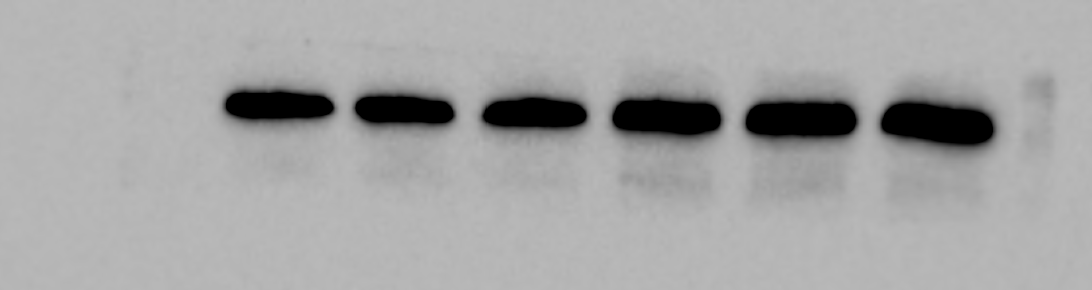

Supplement: Figure 2—source data 4. [file elife-90724-fig2-data4.zip › Complex II.tif]

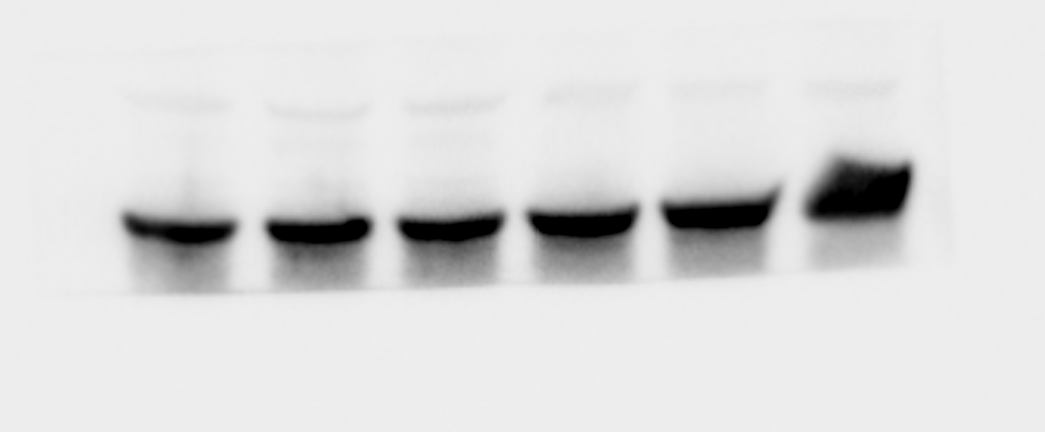

Supplement: Figure 2—source data 4. [file elife-90724-fig2-data4.zip › Complex III.tif]

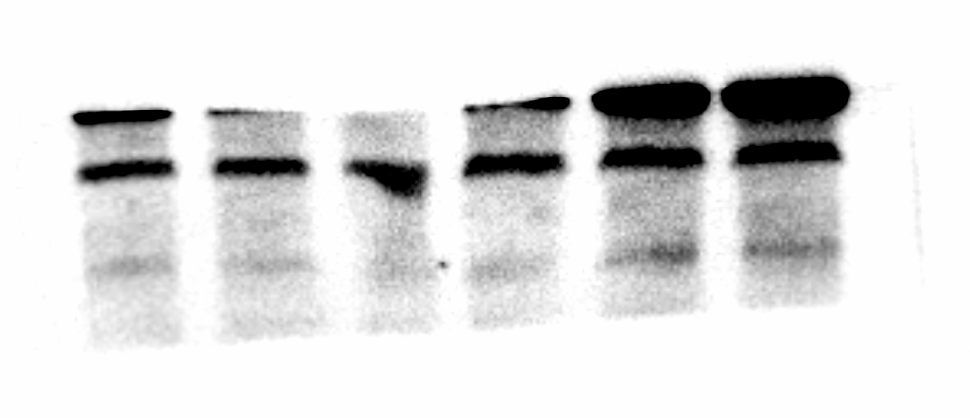

Supplement: Figure 2—source data 4. [file elife-90724-fig2-data4.zip › Complex IV.tif]

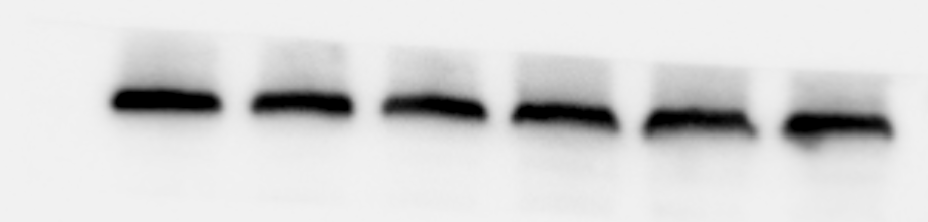

Supplement: Figure 2—source data 4. [file elife-90724-fig2-data4.zip › Complex V.tif]

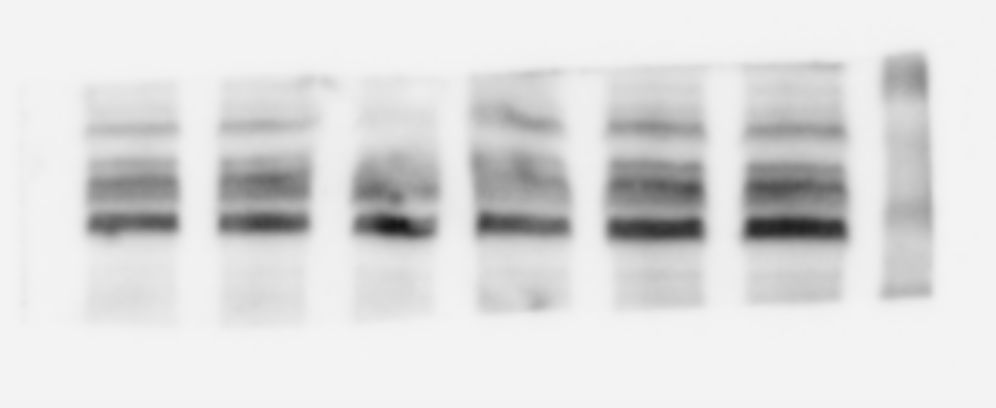

Supplement: Figure 2—source data 4. [file elife-90724-fig2-data4.zip › PGC−1α.tif]

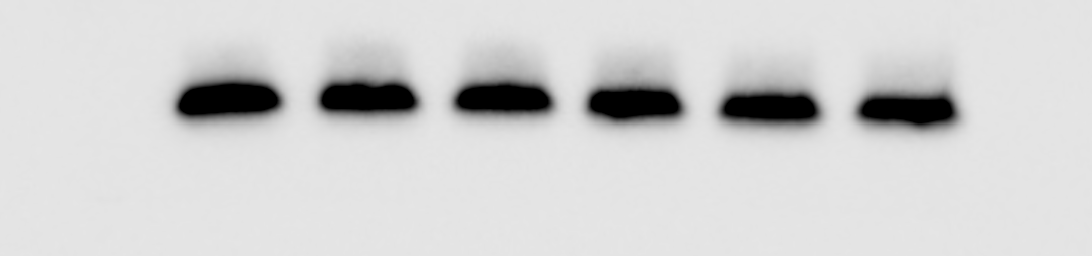

Supplement: Figure 2—source data 4. [file elife-90724-fig2-data4.zip › a┬-Actin.tif]

Figure 2D

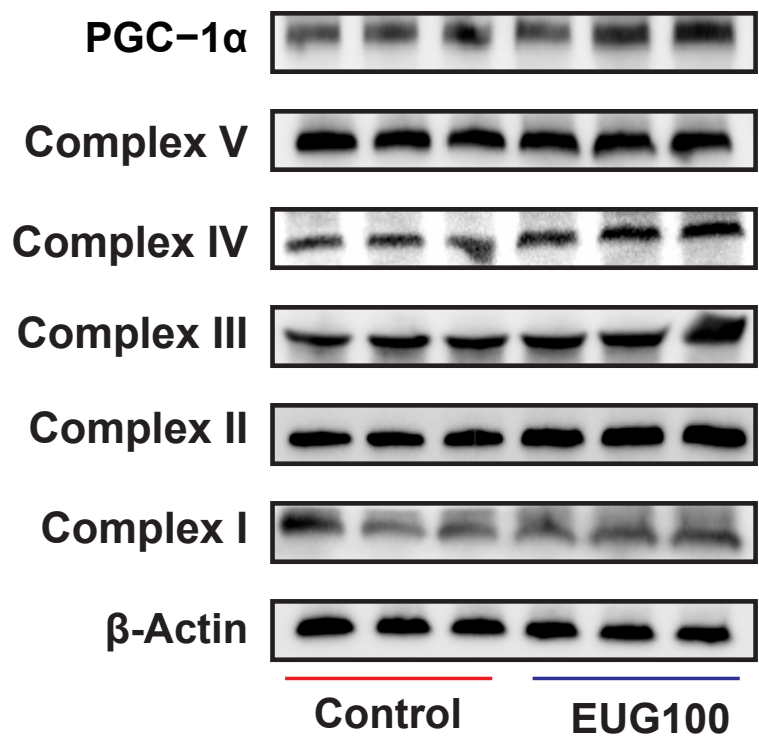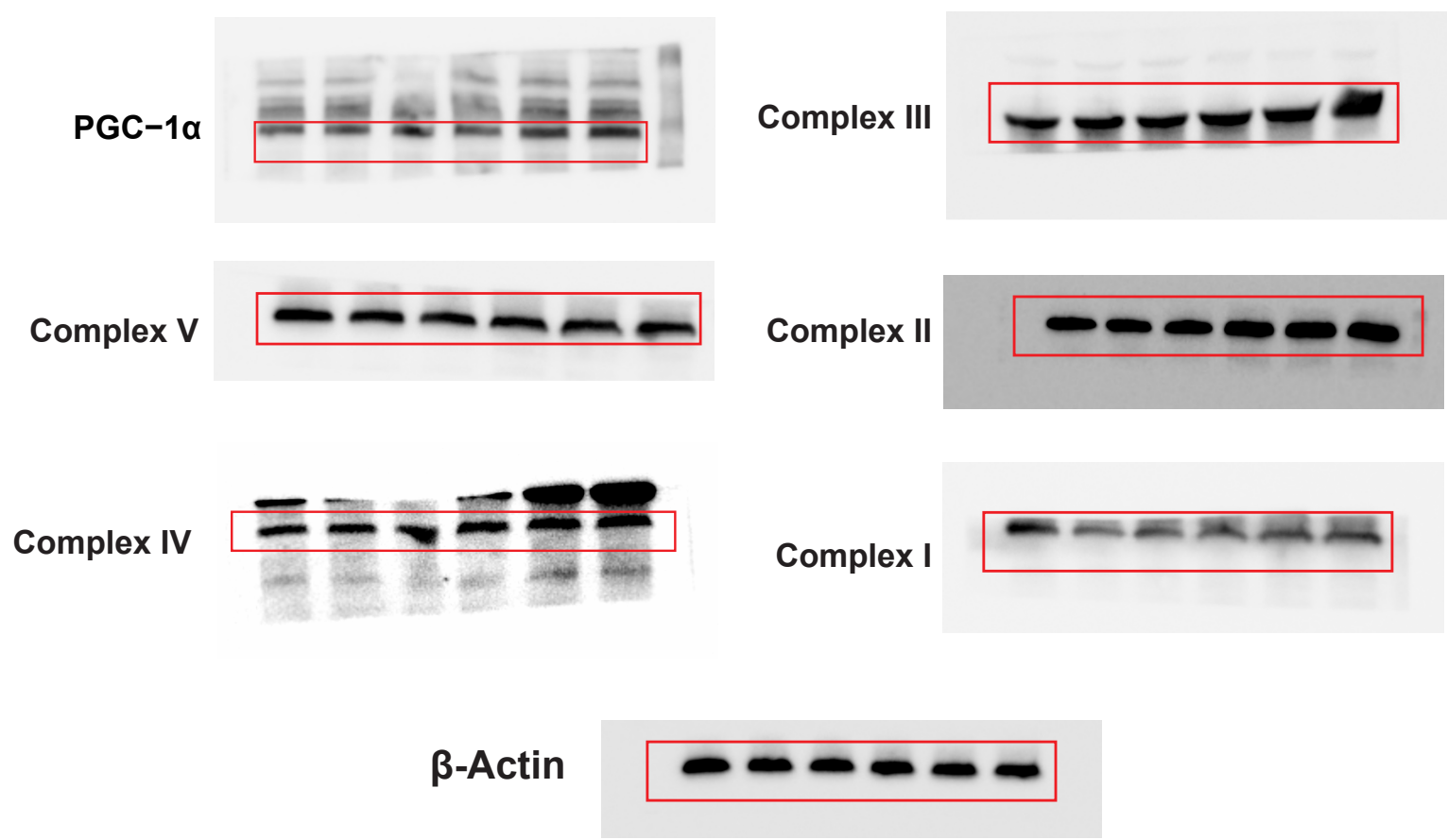

Supplement: Figure 2—source data 5. [file elife-90724-fig2-data5.pdf]

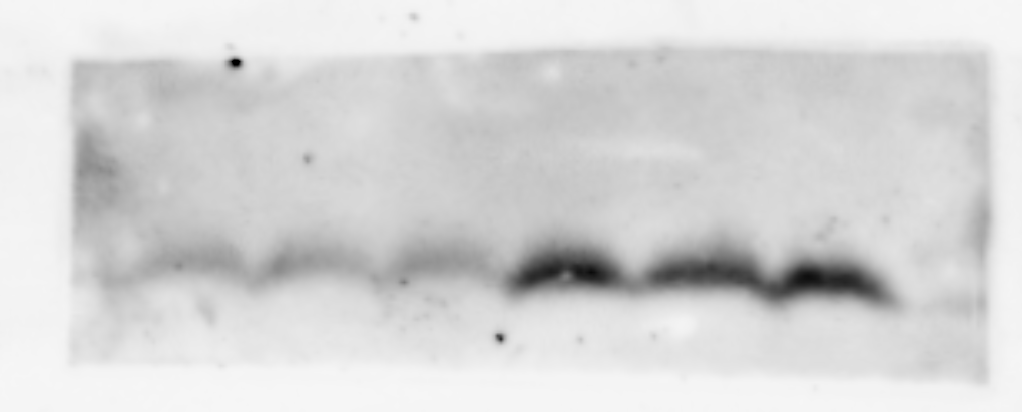

Supplement: Figure 3—source data 6. [file elife-90724-fig3-data6.zip › gWAT-FABP1.tif]

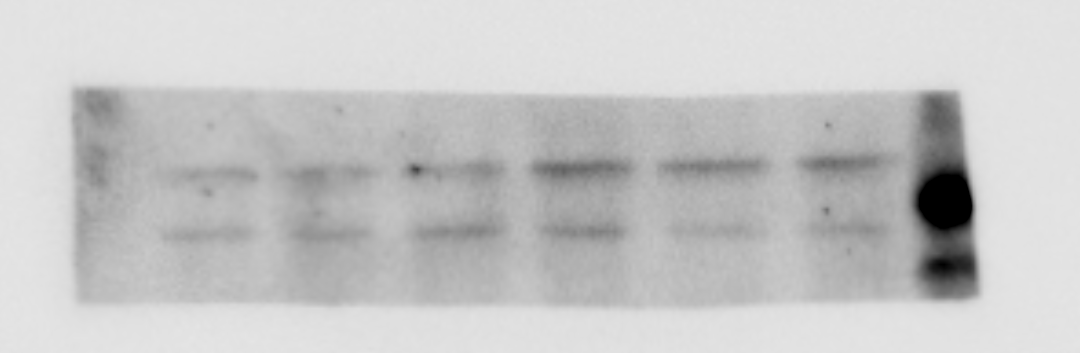

Supplement: Figure 3—source data 6. [file elife-90724-fig3-data6.zip › gWAT-UCP1.tif]

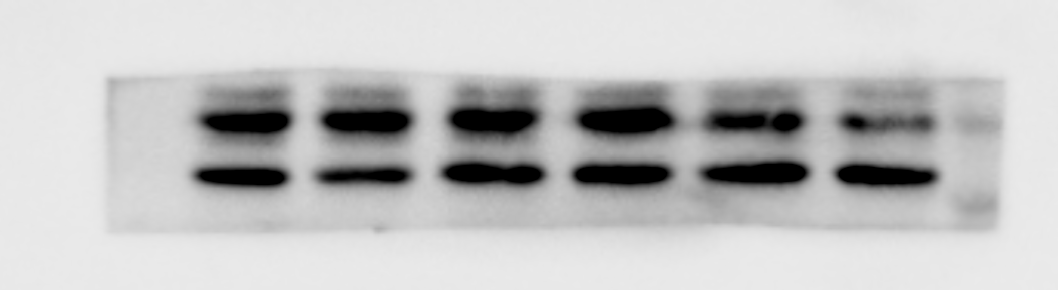

Supplement: Figure 3—source data 6. [file elife-90724-fig3-data6.zip › gWAT-a┬-Actin.tif]

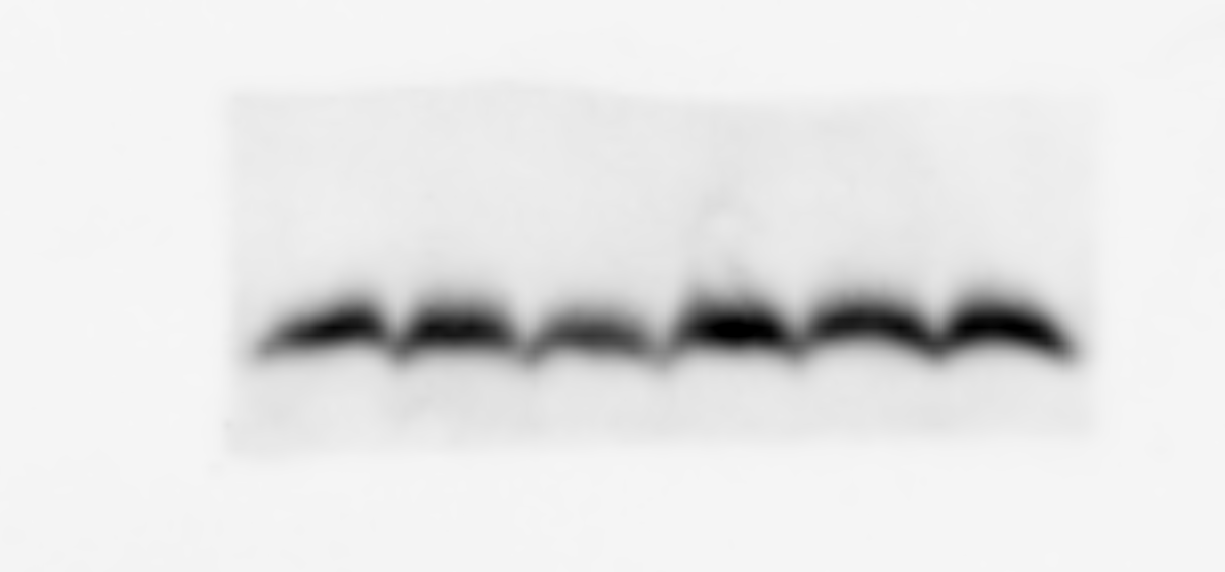

Supplement: Figure 3—source data 6. [file elife-90724-fig3-data6.zip › iWAT-FABP1.tif]

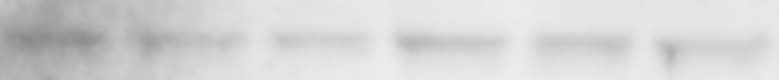

Supplement: Figure 3—source data 6. [file elife-90724-fig3-data6.zip › iWAT-UCP1.tif]

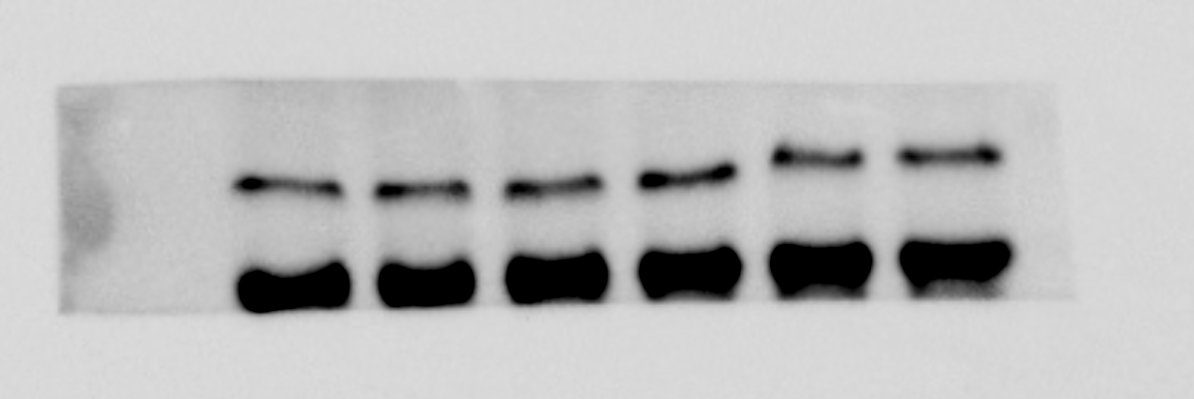

Supplement: Figure 3—source data 6. [file elife-90724-fig3-data6.zip › iWAT-a┬-Actin.tif]

Figure 3F

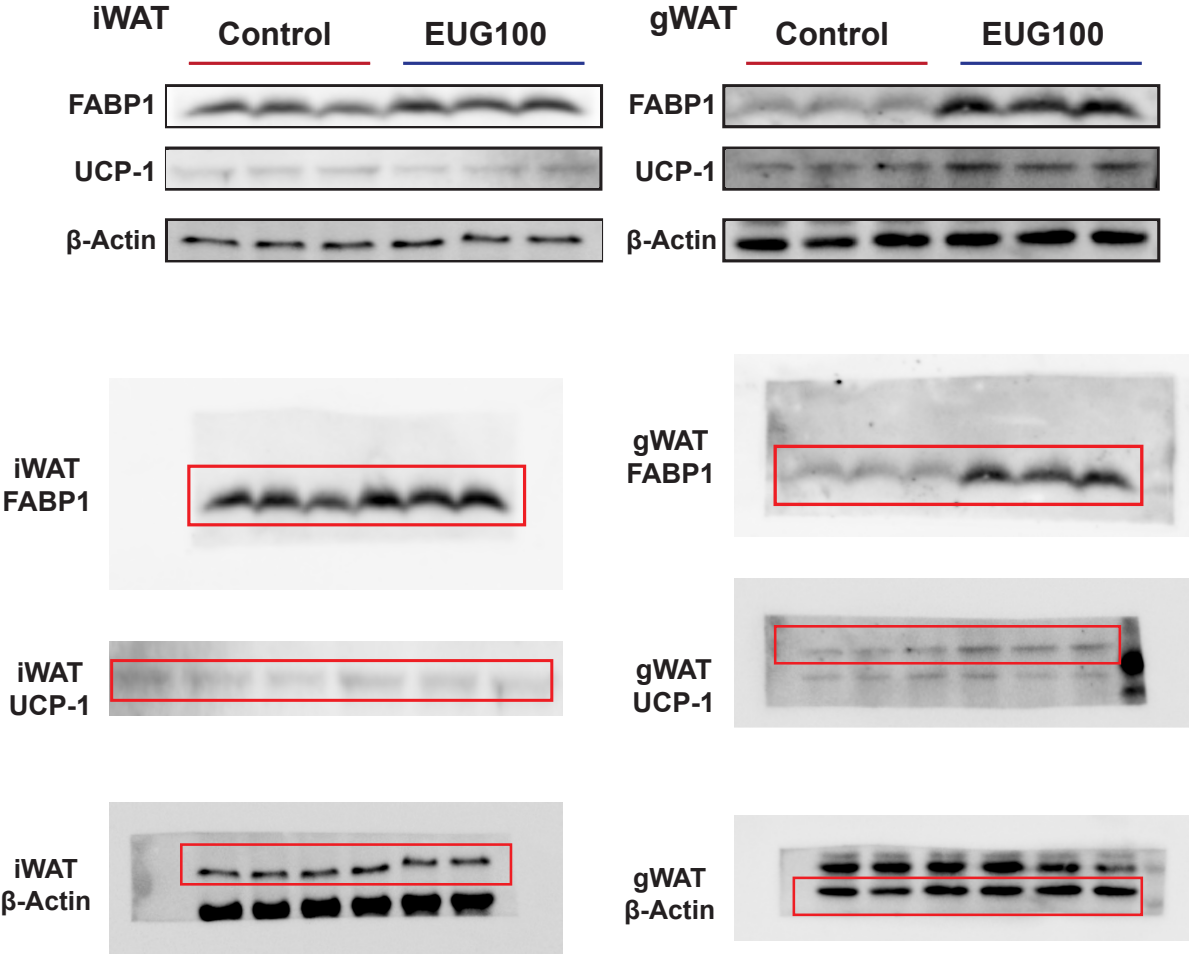

Supplement: Figure 3—source data 7. [file elife-90724-fig3-data7.pdf]

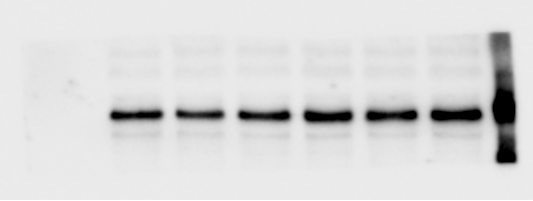

Supplement: Figure 3—source data 8. [file elife-90724-fig3-data8.zip › PGC-1a┴.tif]

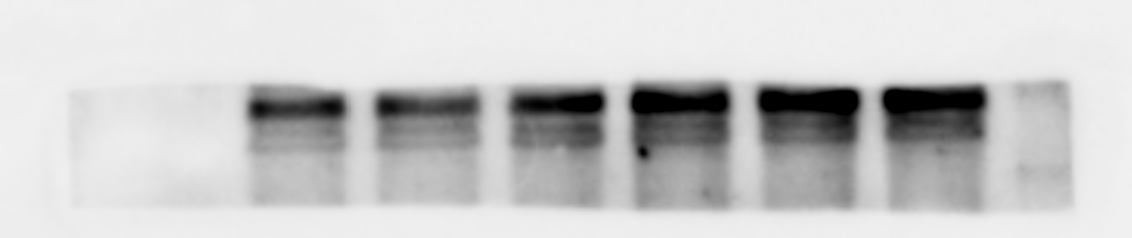

Supplement: Figure 3—source data 8. [file elife-90724-fig3-data8.zip › PRDM16.tif]

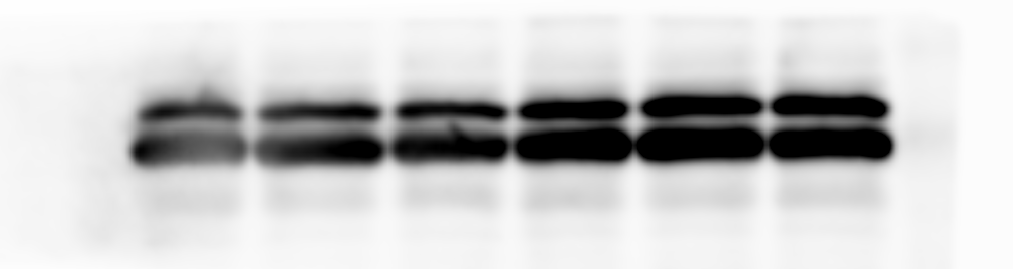

Supplement: Figure 3—source data 8. [file elife-90724-fig3-data8.zip › UCP-1.tif]

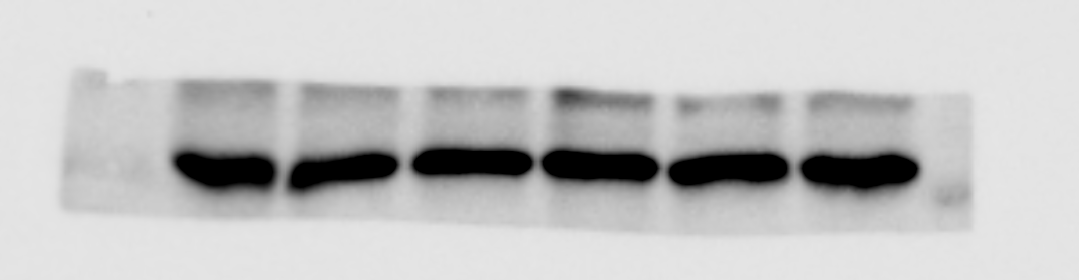

Supplement: Figure 3—source data 8. [file elife-90724-fig3-data8.zip › a┬-Actin.tif]

# Figure 3G

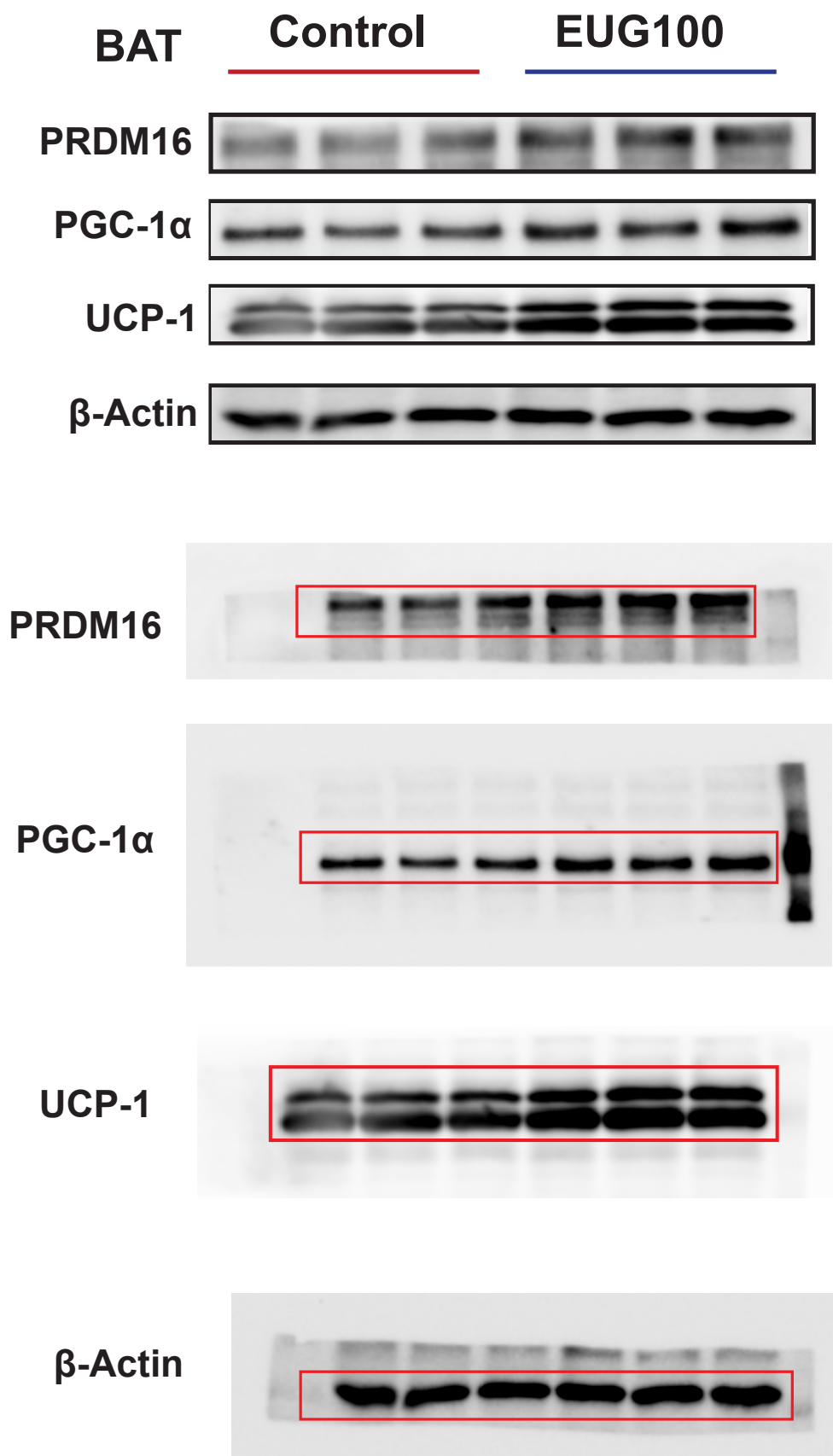

Supplement: Figure 3—source data 9. [file elife-90724-fig3-data9.pdf]

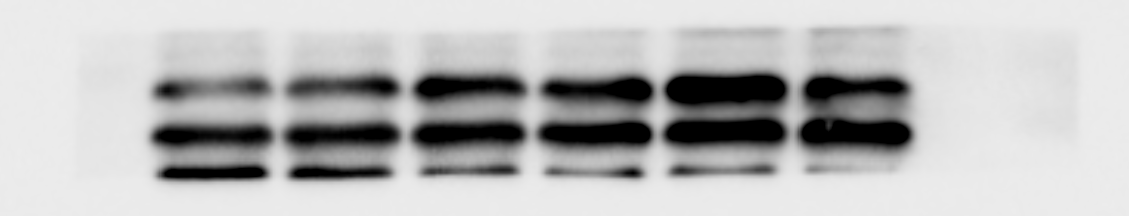

Supplement: Figure 3—source data 10. [file elife-90724-fig3-data10.zip › Complex I.tif]

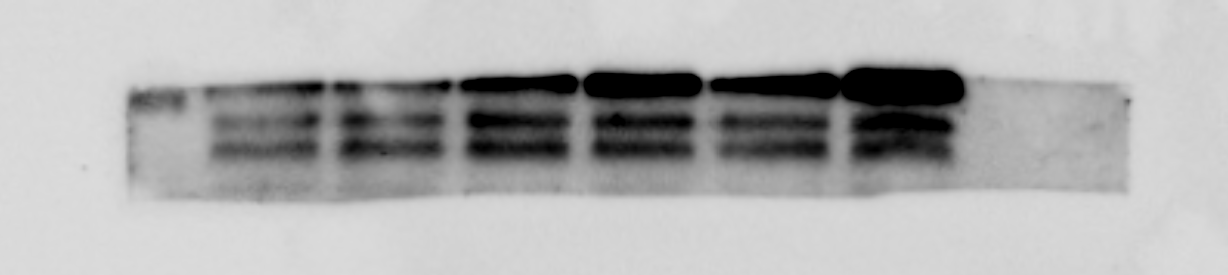

Supplement: Figure 3—source data 10. [file elife-90724-fig3-data10.zip › Complex II.tif]

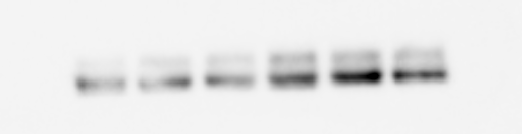

Supplement: Figure 3—source data 10. [file elife-90724-fig3-data10.zip › Complex III.tif]

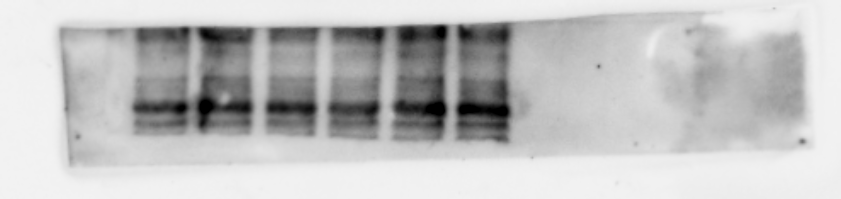

Supplement: Figure 3—source data 10. [file elife-90724-fig3-data10.zip › Complex IV.tif]

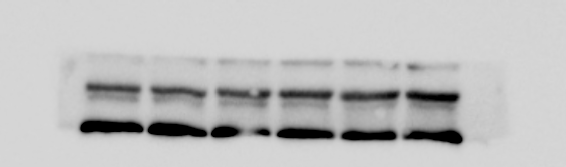

Supplement: Figure 3—source data 10. [file elife-90724-fig3-data10.zip › Complex V.tif]

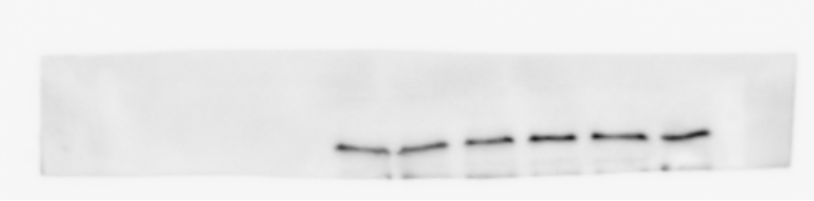

Supplement: Figure 3—source data 10. [file elife-90724-fig3-data10.zip › a┬-Actin.tif]

**Figure 3H**

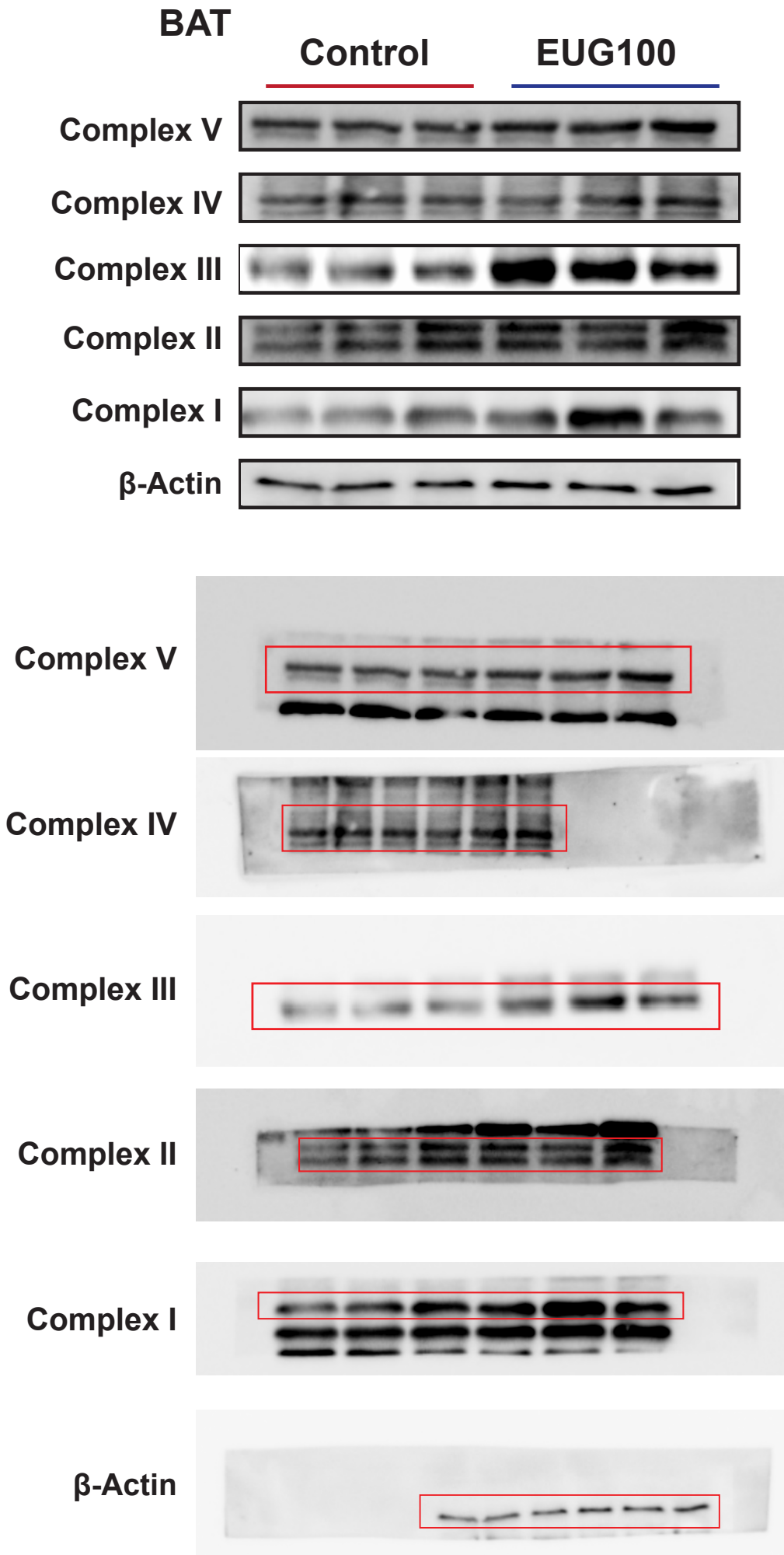

Supplement: Figure 3—source data 11. [file elife-90724-fig3-data11.pdf]

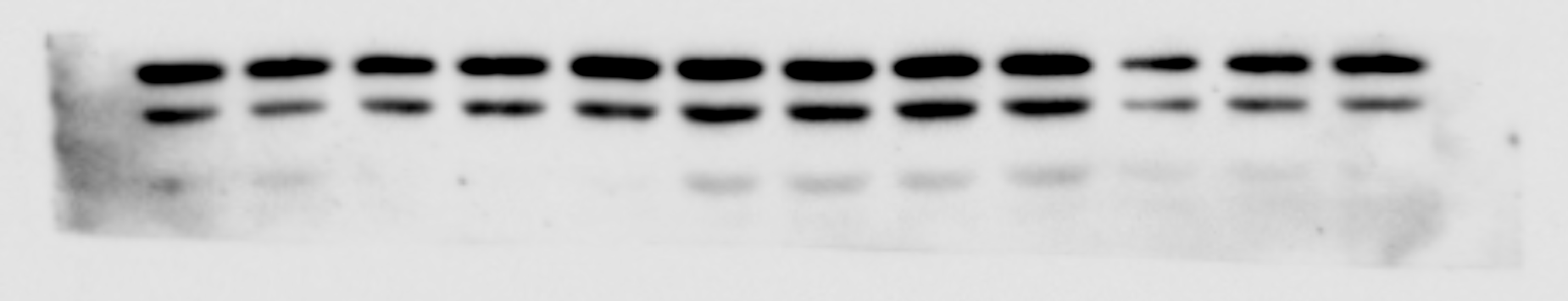

Supplement: Figure 4—source data 4. [file elife-90724-fig4-data4.zip › CnA.tif]

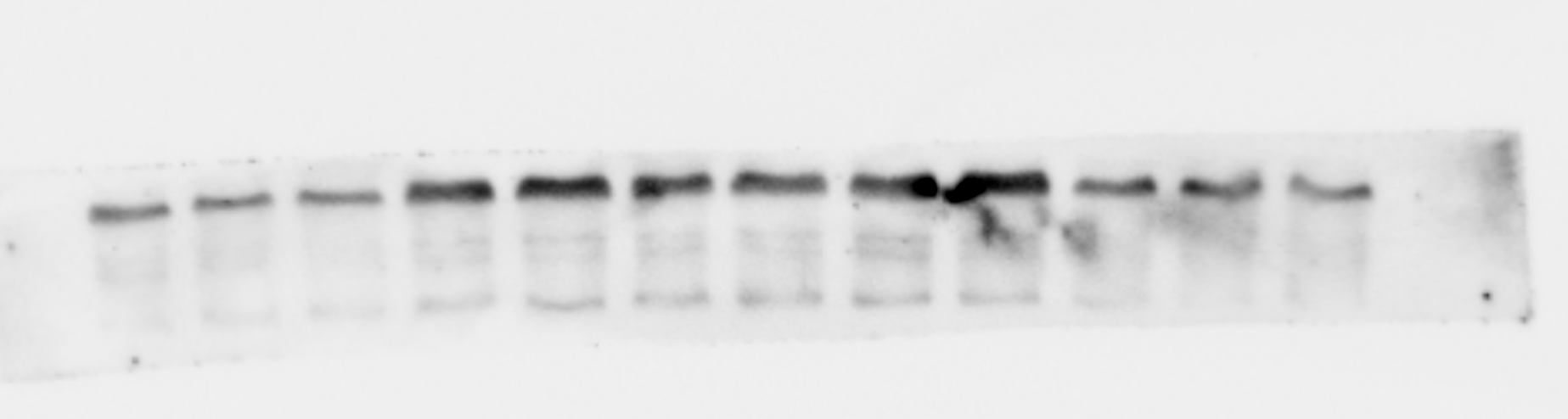

Supplement: Figure 4—source data 4. [file elife-90724-fig4-data4.zip › TRPV1.tif]

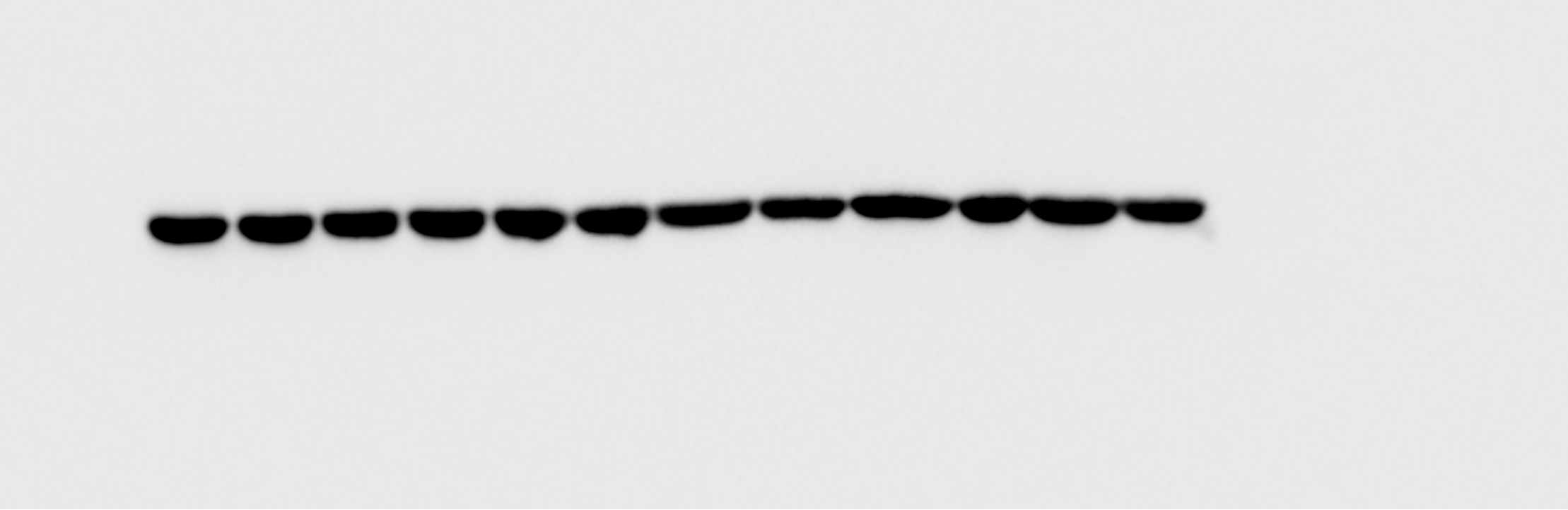

Supplement: Figure 4—source data 4. [file elife-90724-fig4-data4.zip › a┬-Actin.tif]

# Figure 4D

GAS muscle  
total protein

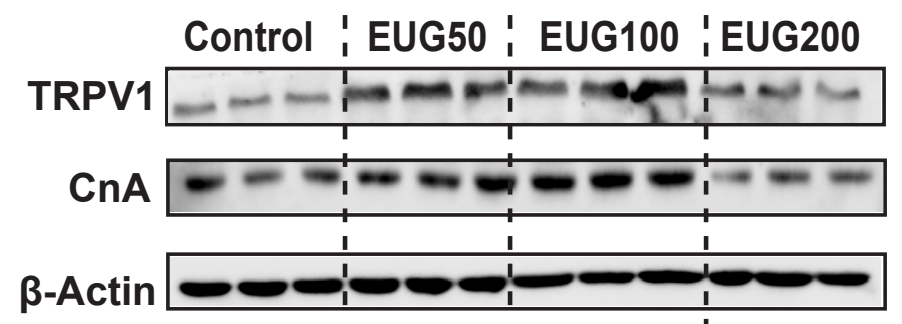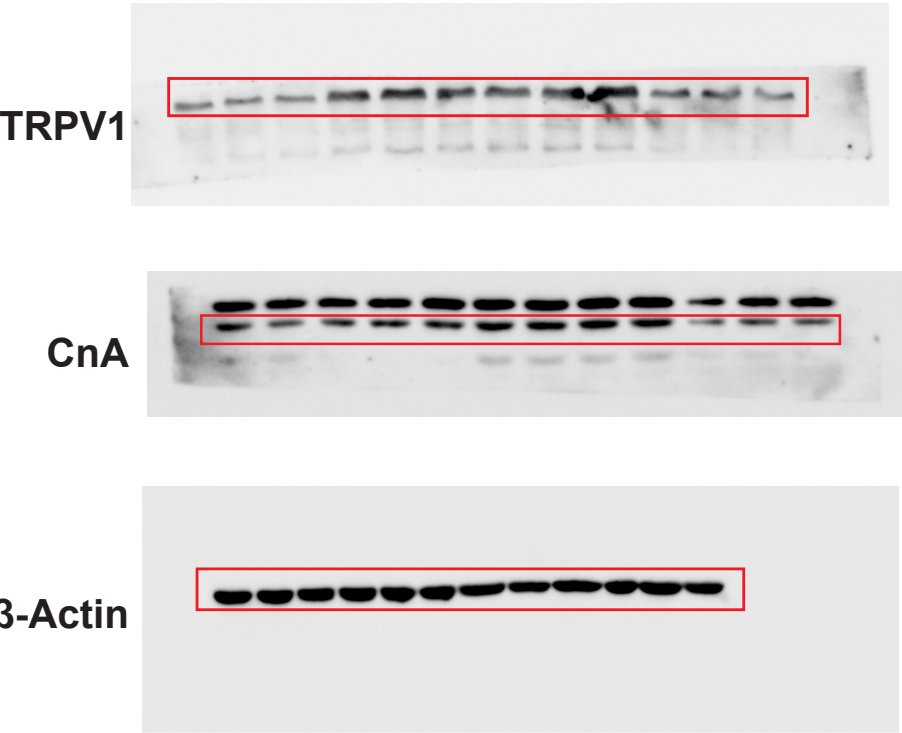

Supplement: Figure 4—source data 5. [file elife-90724-fig4-data5.pdf]

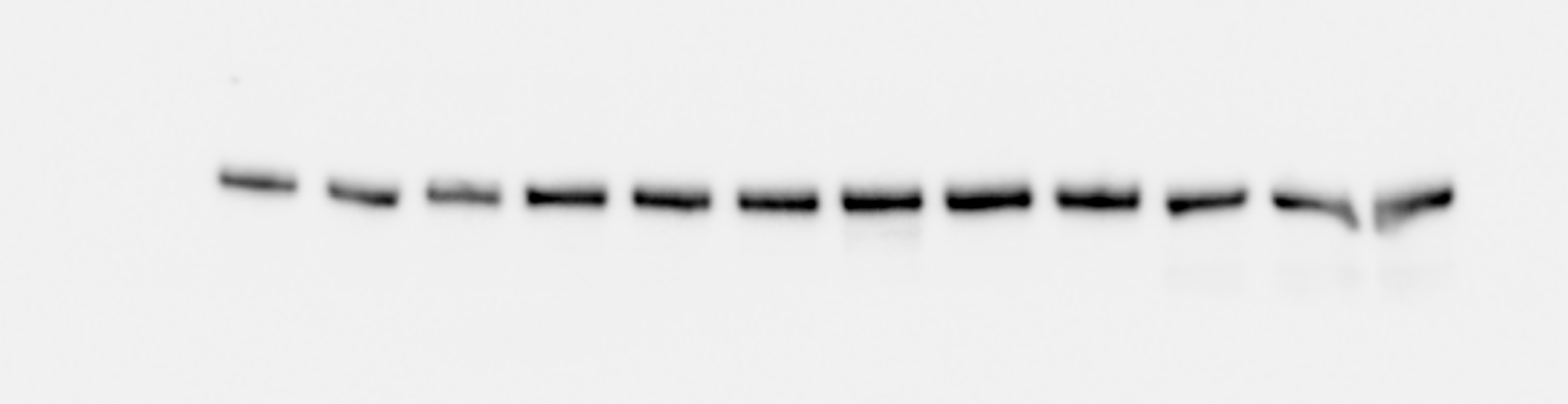

Supplement: Figure 4—source data 6. [file elife-90724-fig4-data6.zip › CnA.tif]

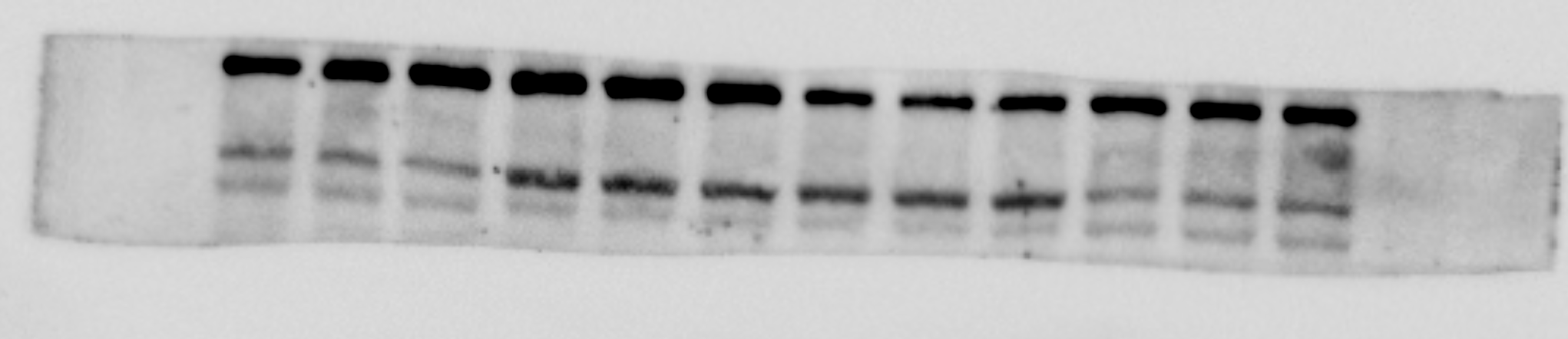

Supplement: Figure 4—source data 6. [file elife-90724-fig4-data6.zip › TRPV1.tif]

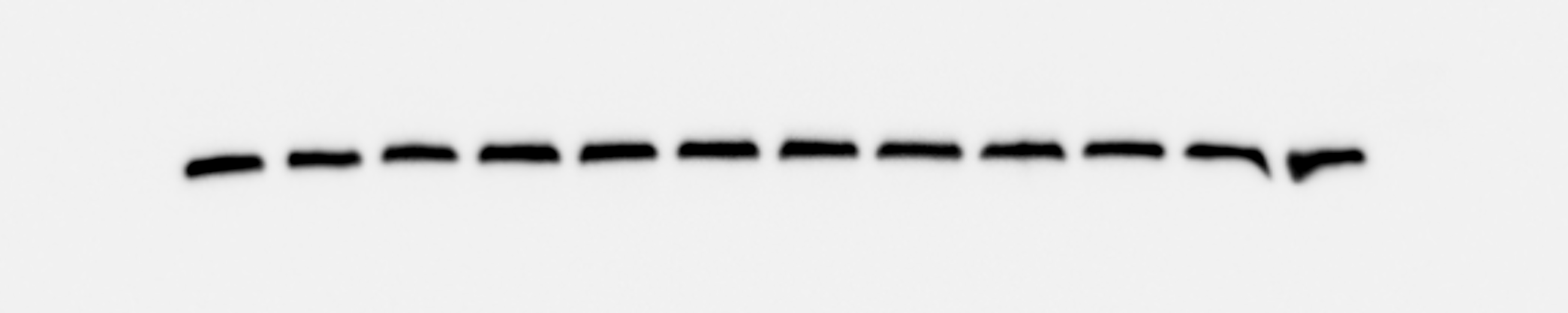

Supplement: Figure 4—source data 6. [file elife-90724-fig4-data6.zip › a┬-Actin.tif]

Figure 4E

TA muscle  
total protein

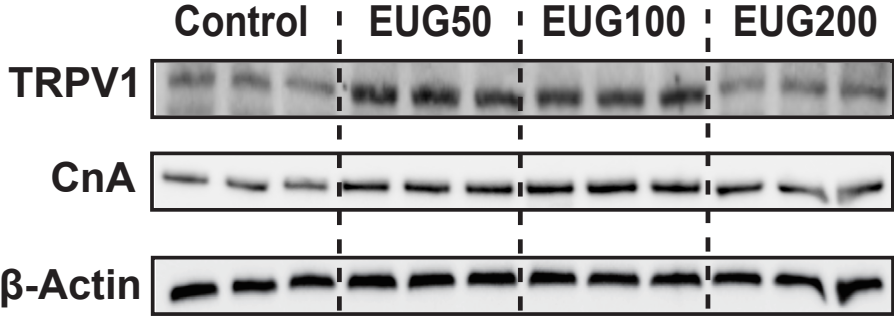

TRPV1

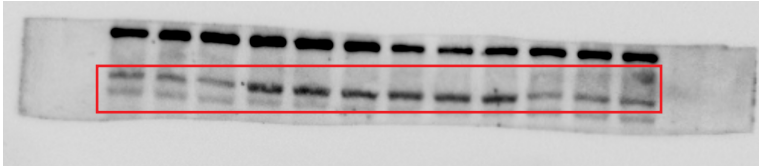

CnA

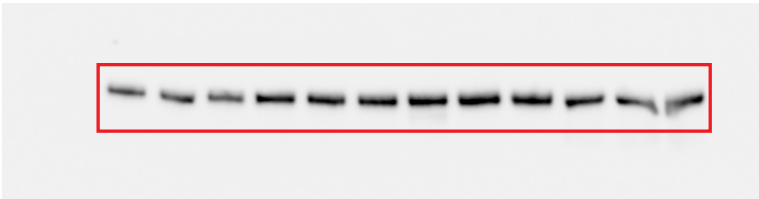

β-Actin

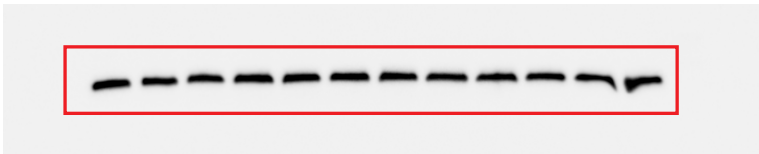

Supplement: Figure 4—source data 7. [file elife-90724-fig4-data7.pdf]

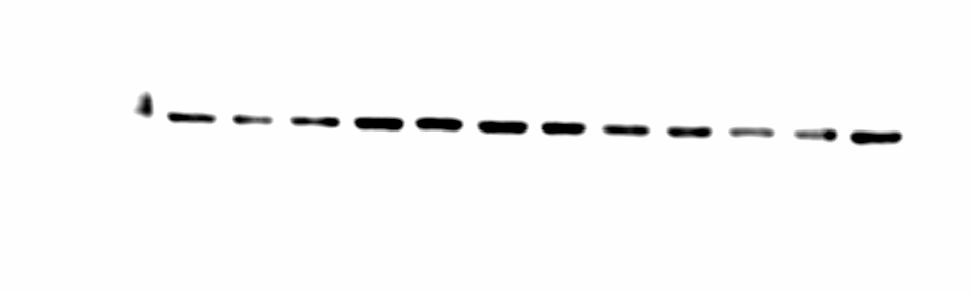

Supplement: Figure 4—source data 8. [file elife-90724-fig4-data8.zip › CnA.tif]

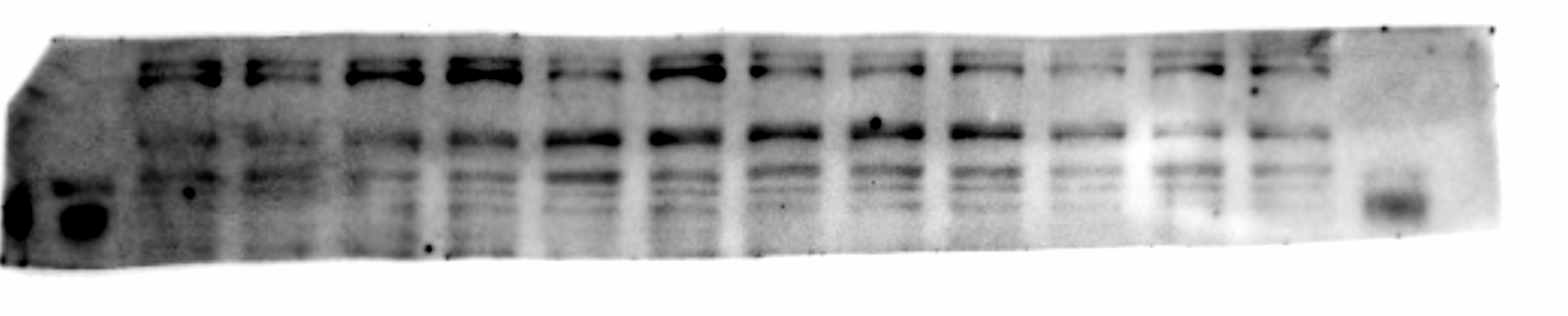

Supplement: Figure 4—source data 8. [file elife-90724-fig4-data8.zip › TRPV1.tif]

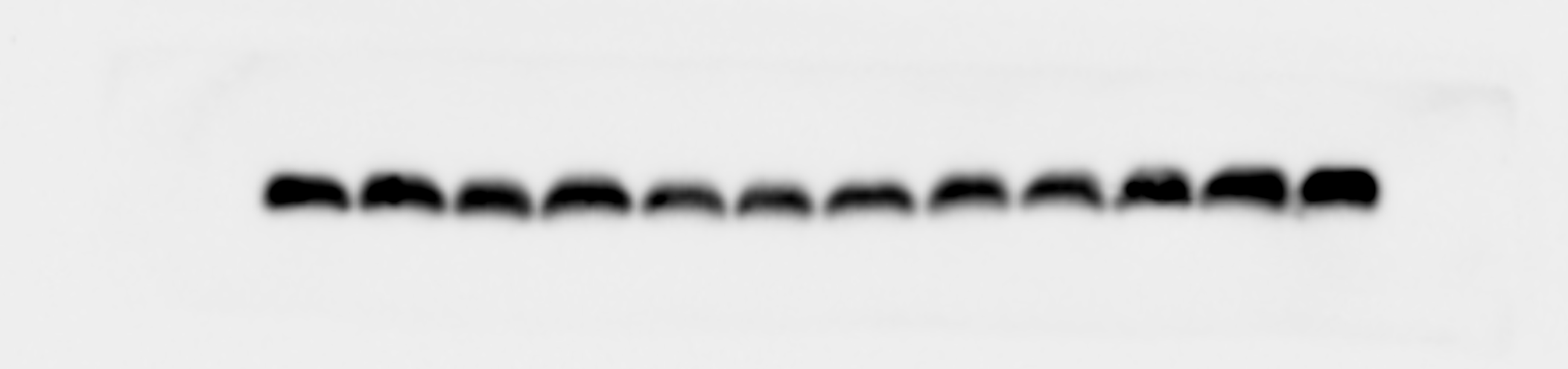

Supplement: Figure 4—source data 8. [file elife-90724-fig4-data8.zip › a┬-Actin.tif]

Figure 4F

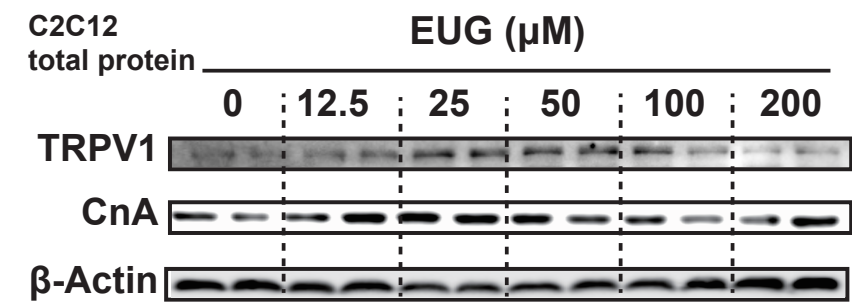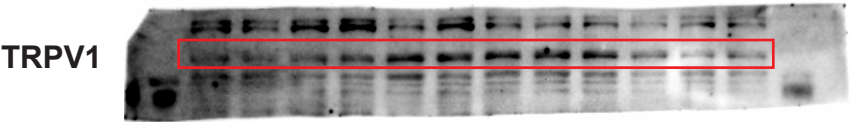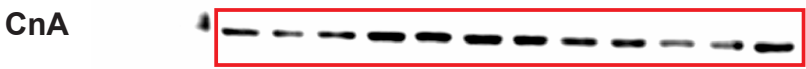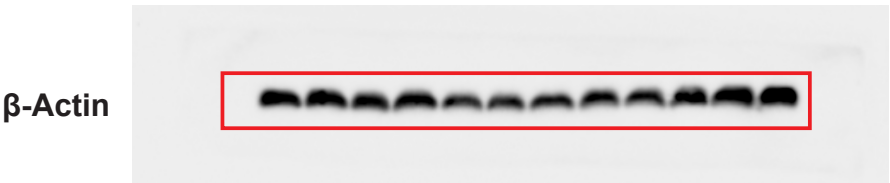

Supplement: Figure 4—source data 9. [file elife-90724-fig4-data9.pdf]

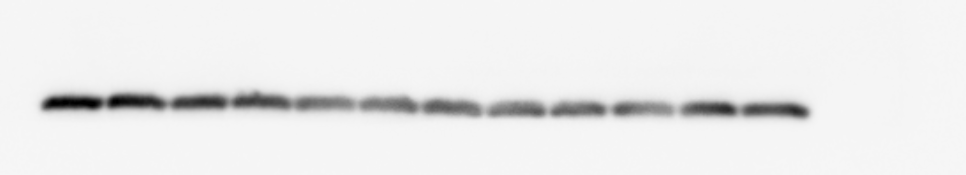

Supplement: Figure 4—source data 10. [file elife-90724-fig4-data10.zip › Histone H3.tif]

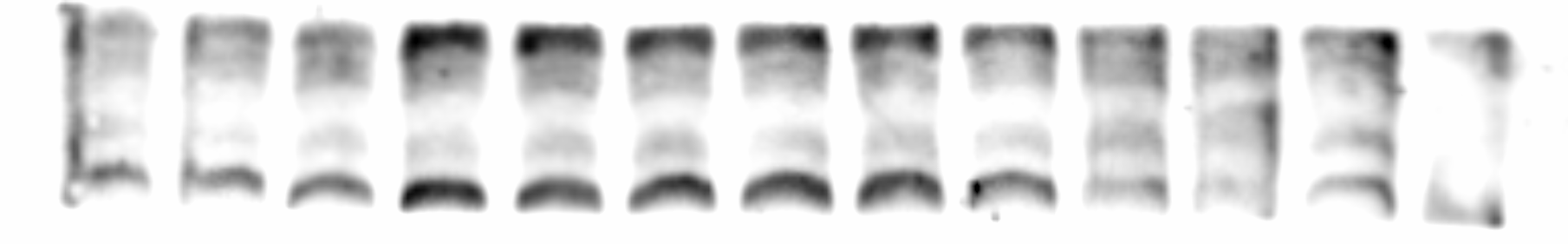

Supplement: Figure 4—source data 10. [file elife-90724-fig4-data10.zip › NFATc1.tif]

# Figure 4G

GAS muscle  
nuclear protein

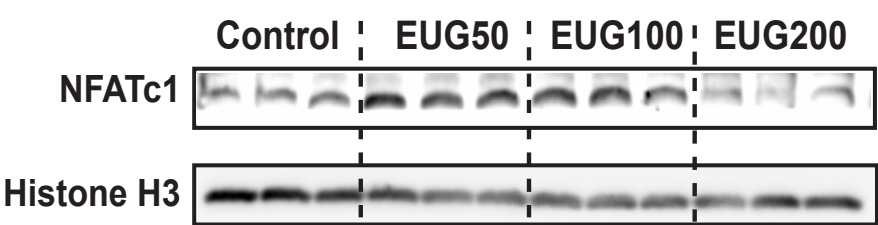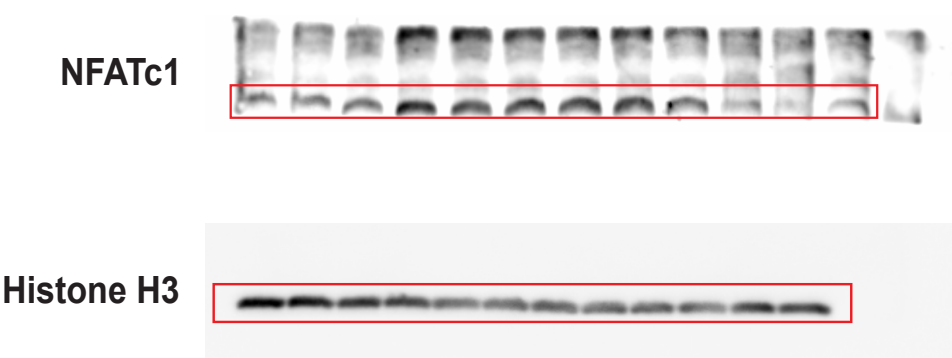

Supplement: Figure 4—source data 11. [file elife-90724-fig4-data11.pdf]

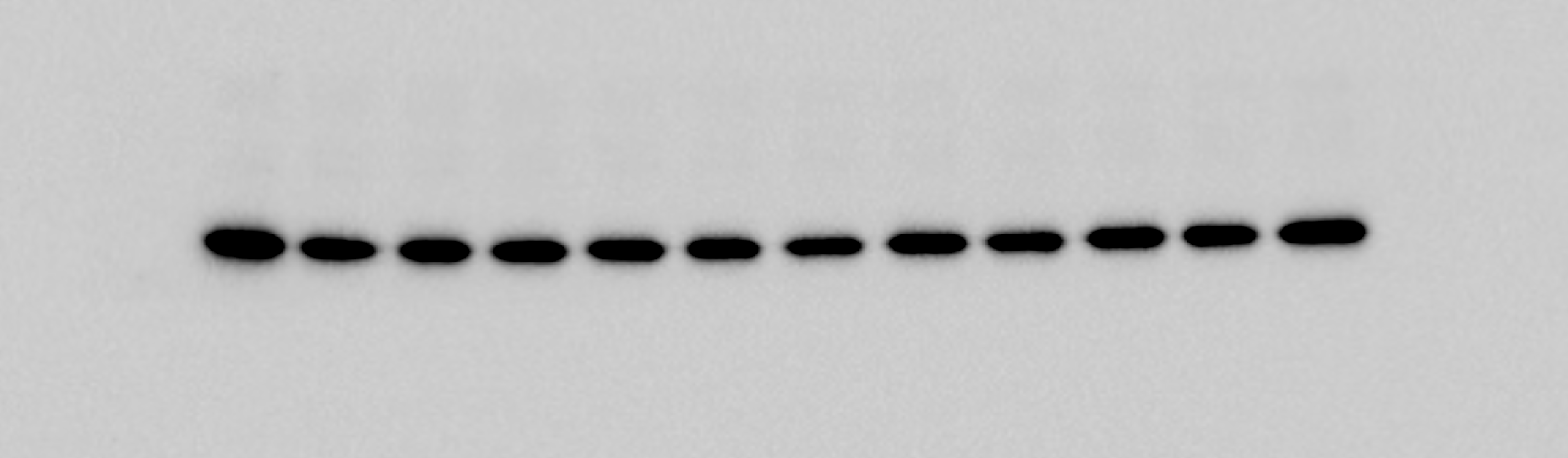

Supplement: Figure 4—source data 12. [file elife-90724-fig4-data12.zip › Histone H3.tif]

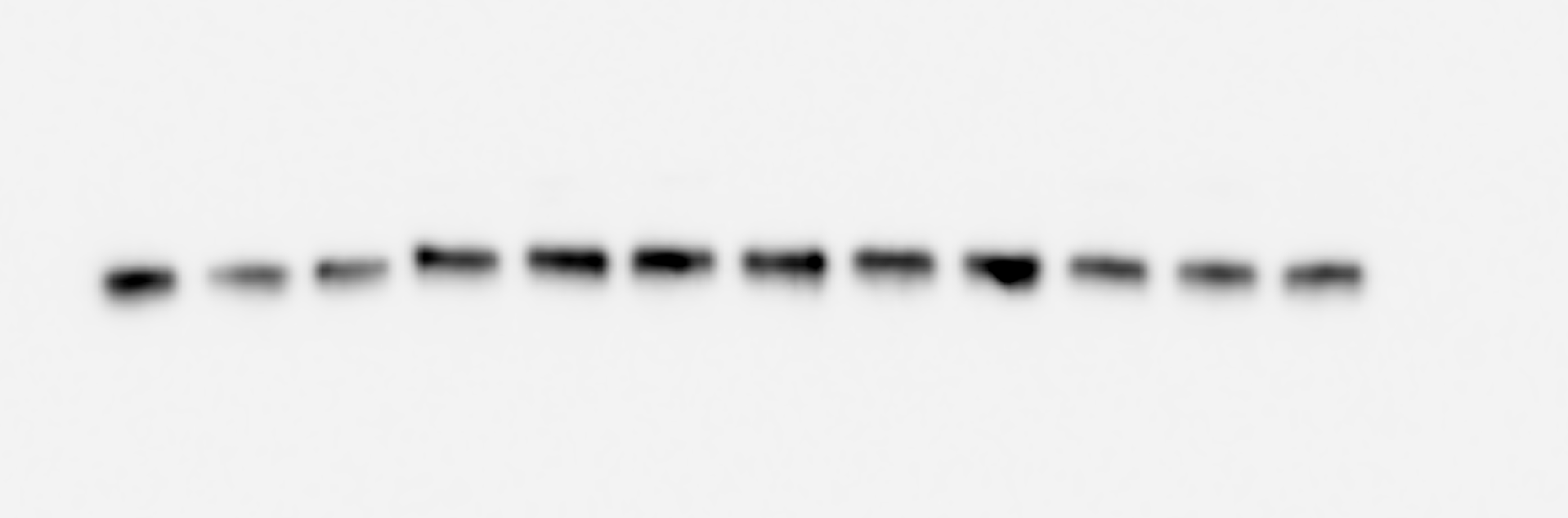

Supplement: Figure 4—source data 12. [file elife-90724-fig4-data12.zip › NFATc1.tif]

Figure 4H

TA muscle  
nuclear protein

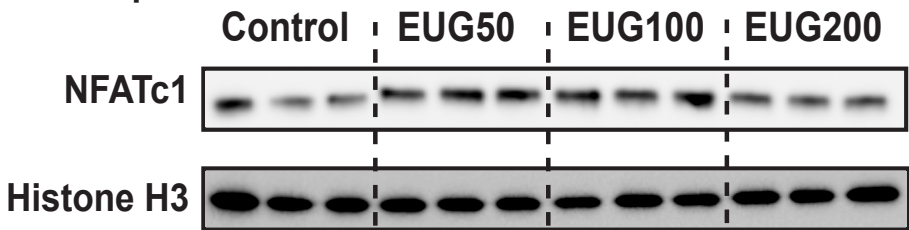

NFATc1

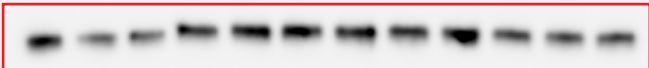

Histone H3

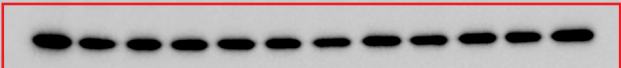

Supplement: Figure 4—source data 13. [file elife-90724-fig4-data13.pdf]

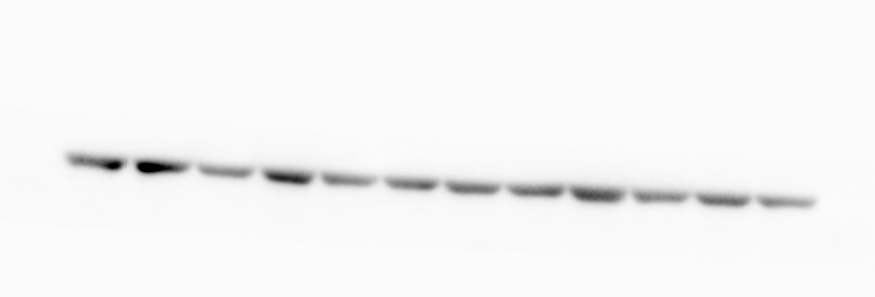

Supplement: Figure 4—source data 14. [file elife-90724-fig4-data14.zip › Histone H3.tif]

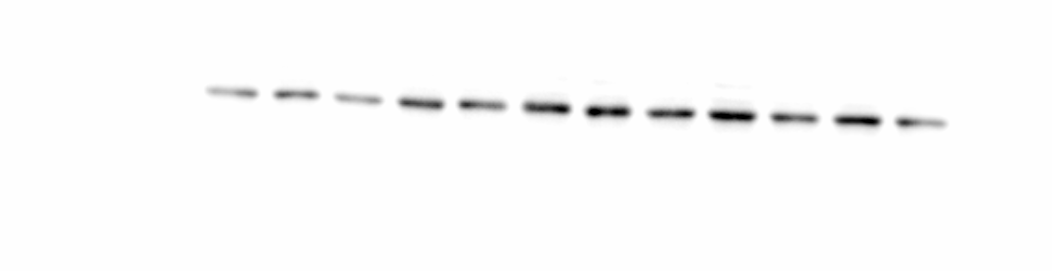

Supplement: Figure 4—source data 14. [file elife-90724-fig4-data14.zip › NFATc1.tif]

**Figure 4I**

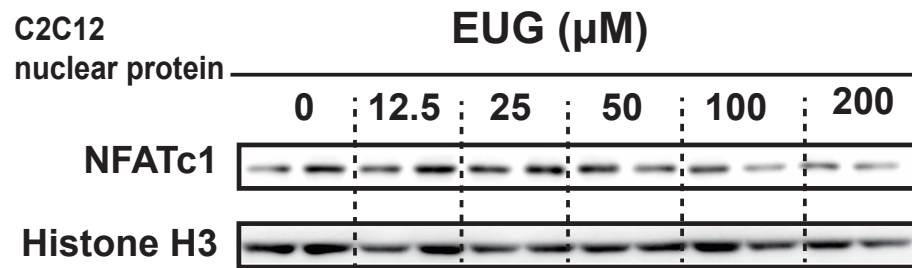

**NFATc1**

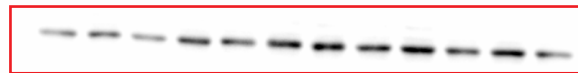

**Histone H3**

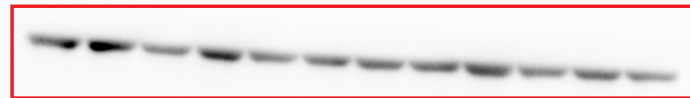

Supplement: Figure 4—source data 15. [file elife-90724-fig4-data15.pdf]

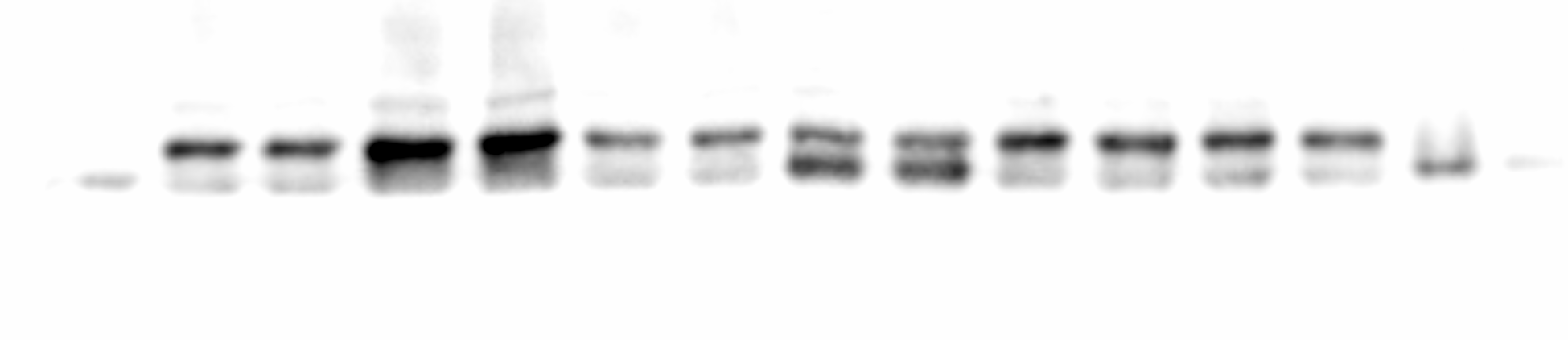

Supplement: Figure 5—source data 2. [file elife-90724-fig5-data2.zip › CnA.tif]

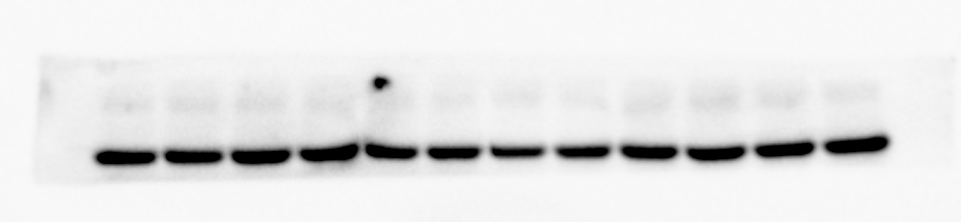

Supplement: Figure 5—source data 2. [file elife-90724-fig5-data2.zip › a┬-Actin.tif]

**Figure 5B**

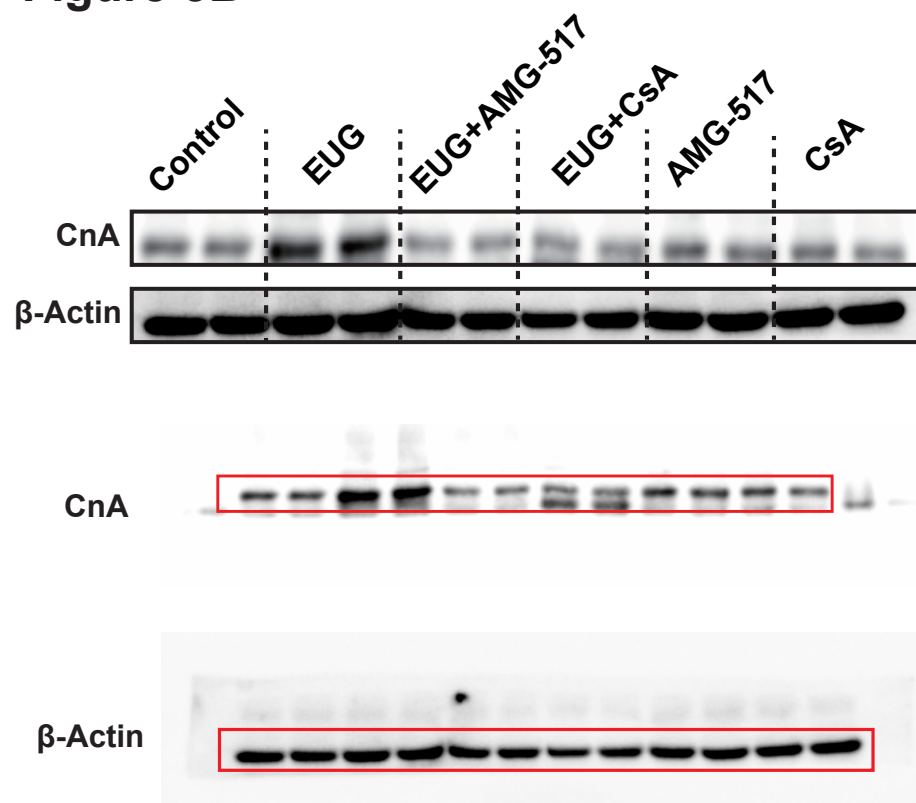

Supplement: Figure 5—source data 3. [file elife-90724-fig5-data3.pdf]

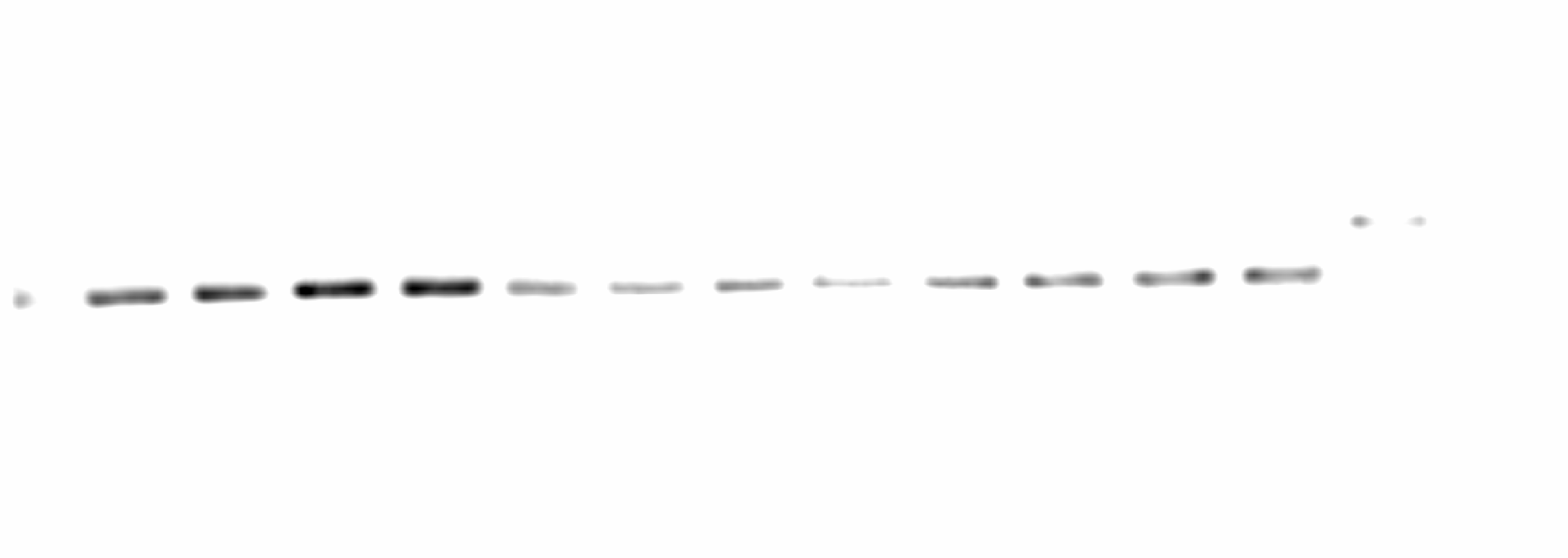

Supplement: Figure 5—source data 4. [file elife-90724-fig5-data4.zip › Complex I.tif]

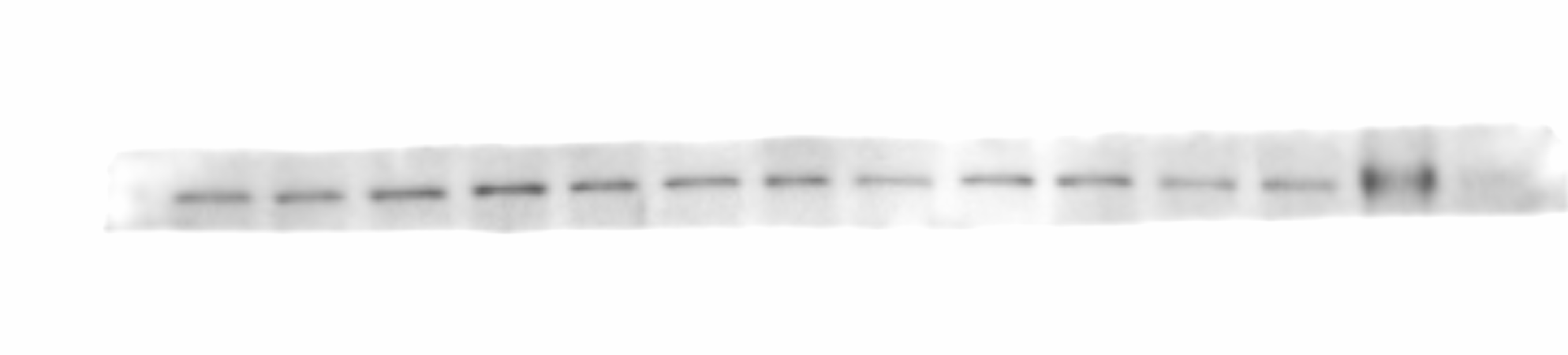

Supplement: Figure 5—source data 4. [file elife-90724-fig5-data4.zip › Complex II.tif]

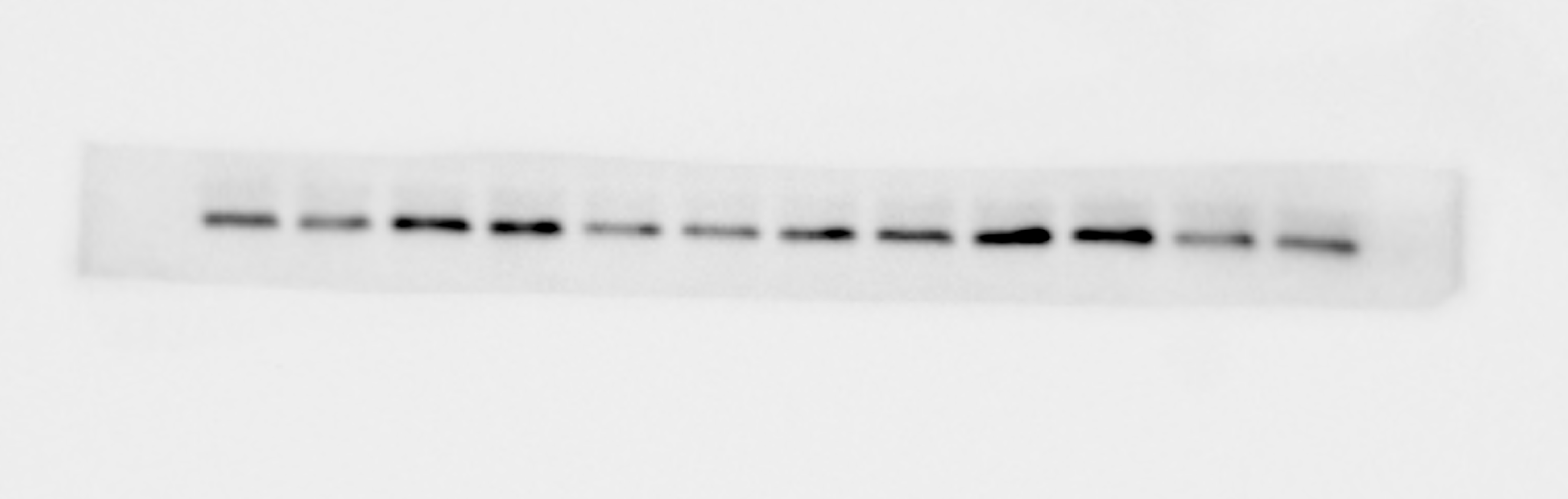

Supplement: Figure 5—source data 4. [file elife-90724-fig5-data4.zip › Complex III.tif]

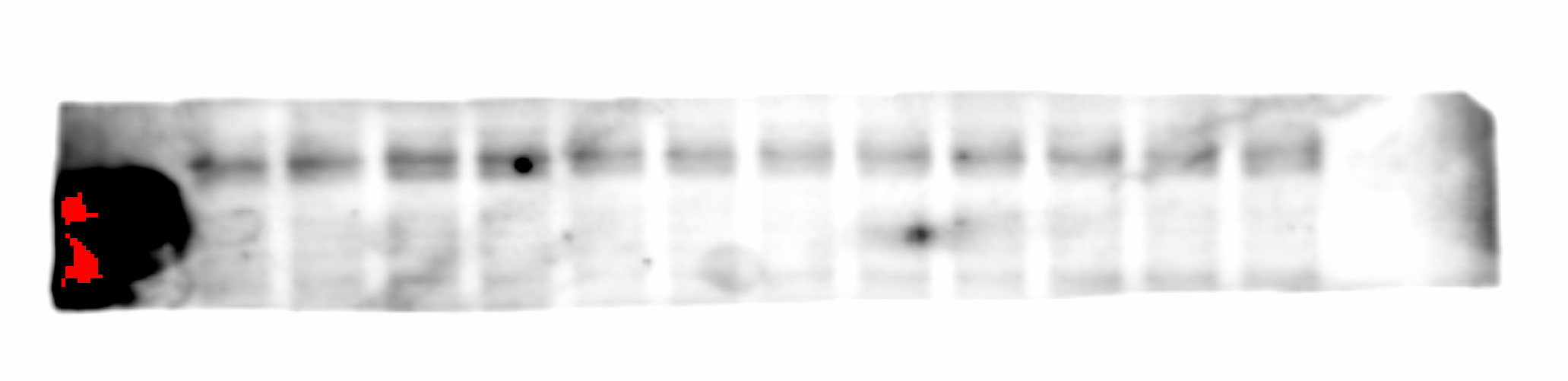

Supplement: Figure 5—source data 4. [file elife-90724-fig5-data4.zip › Complex IV.tif]

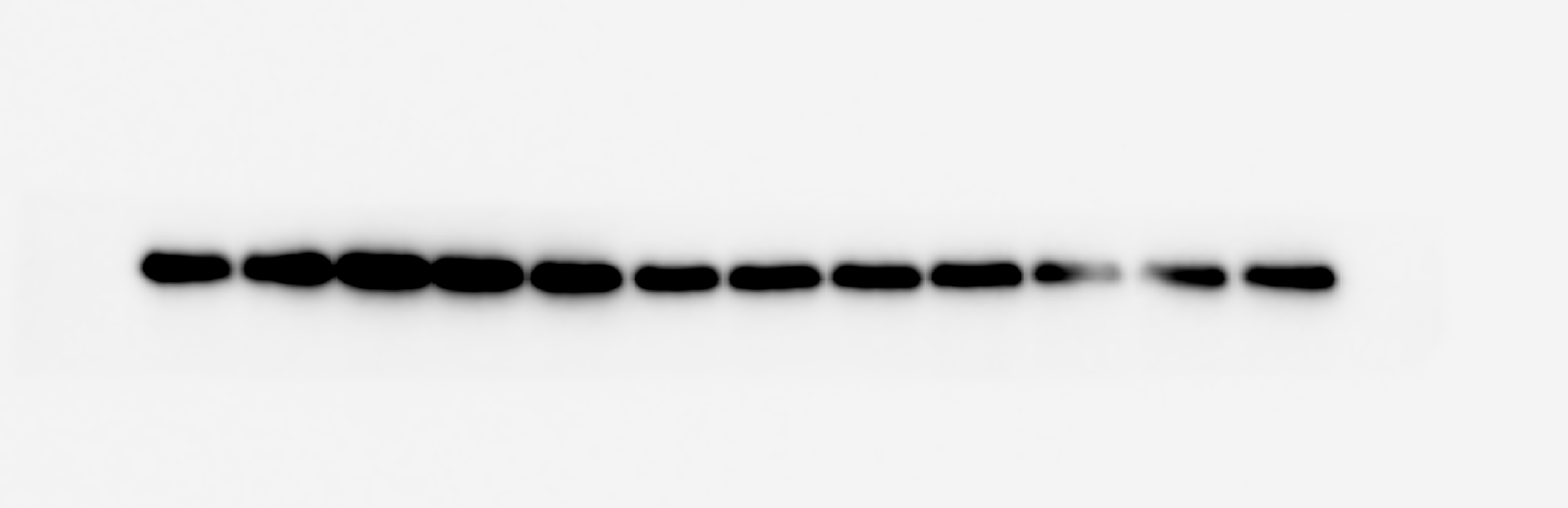

Supplement: Figure 5—source data 4. [file elife-90724-fig5-data4.zip › Complex V.tif]

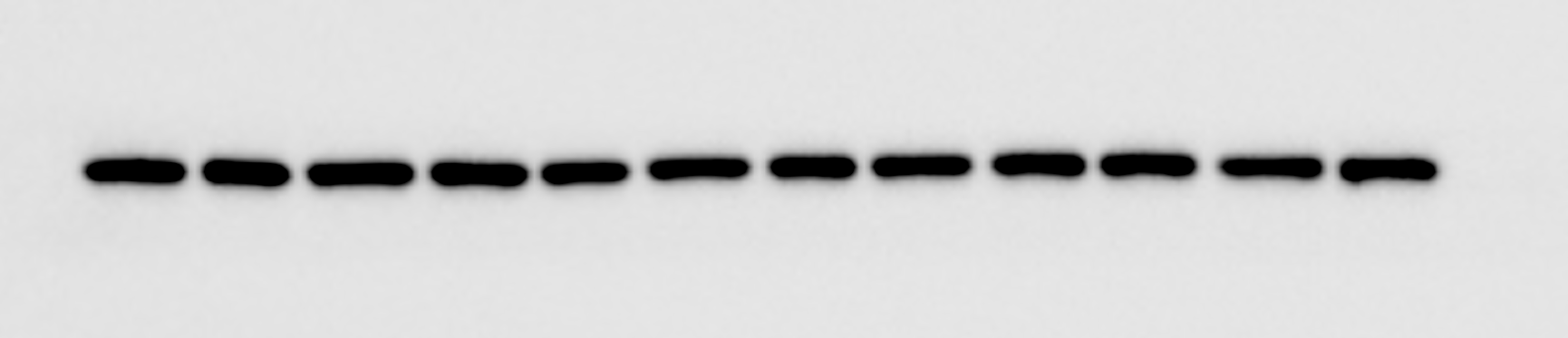

Supplement: Figure 5—source data 4. [file elife-90724-fig5-data4.zip › a┬-Actin.tif]

**Figure 5C**

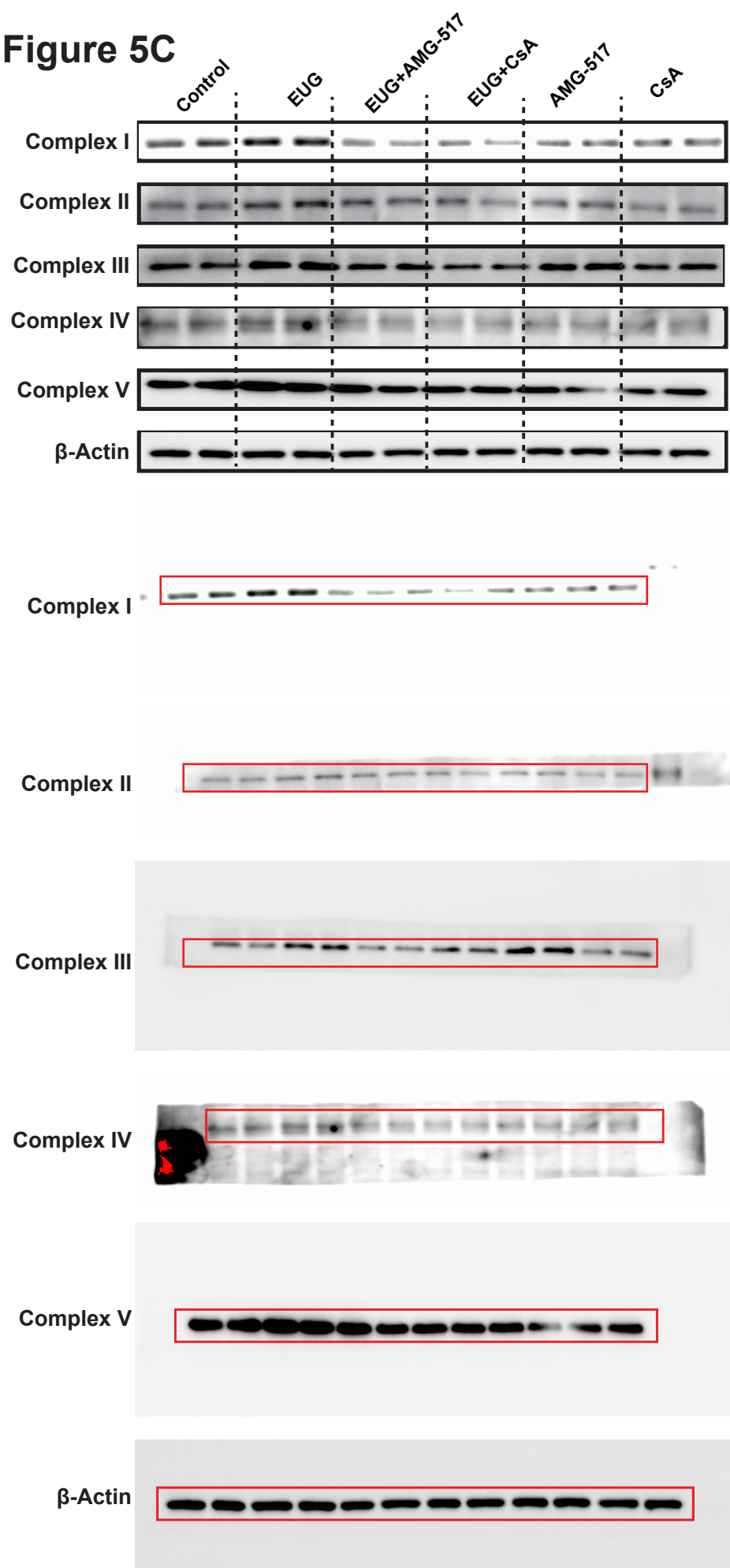

Supplement: Figure 5—source data 5. [file elife-90724-fig5-data5.pdf]

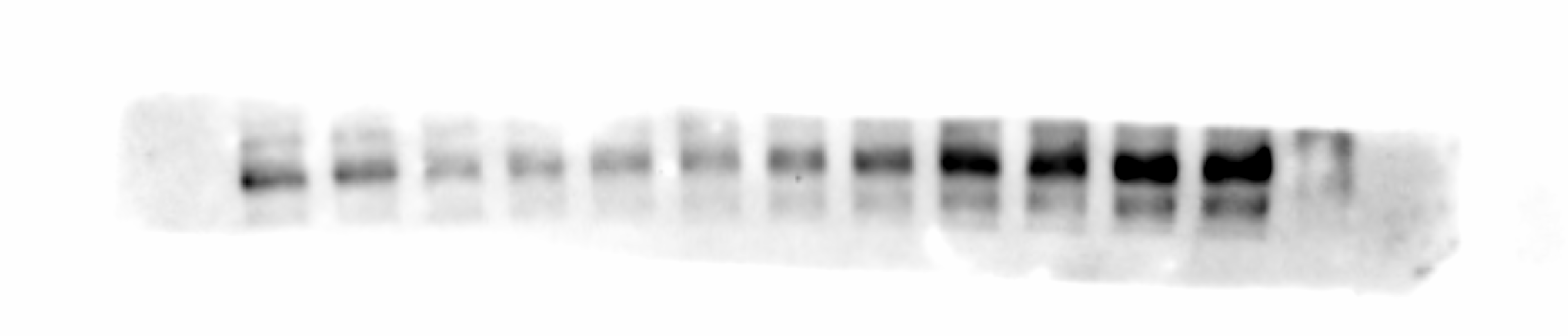

Supplement: Figure 5—source data 6. [file elife-90724-fig5-data6.zip › Fast MyHC .tif]

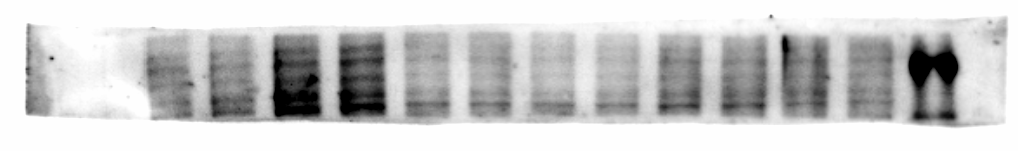

Supplement: Figure 5—source data 6. [file elife-90724-fig5-data6.zip › Slow MyHC.tif]

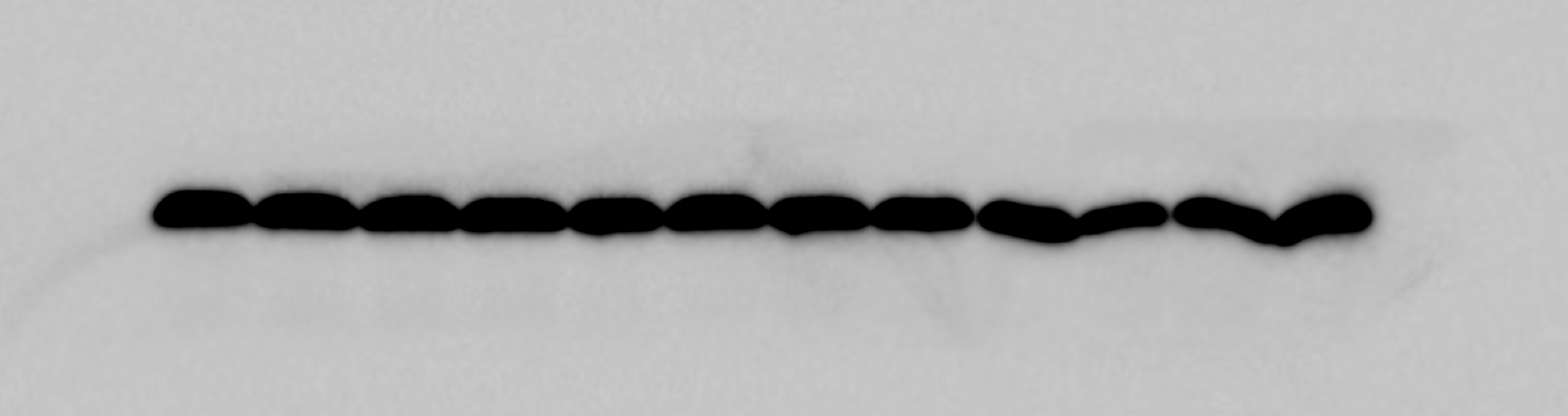

Supplement: Figure 5—source data 6. [file elife-90724-fig5-data6.zip › a┬-Actin.tif]

Figure 5D

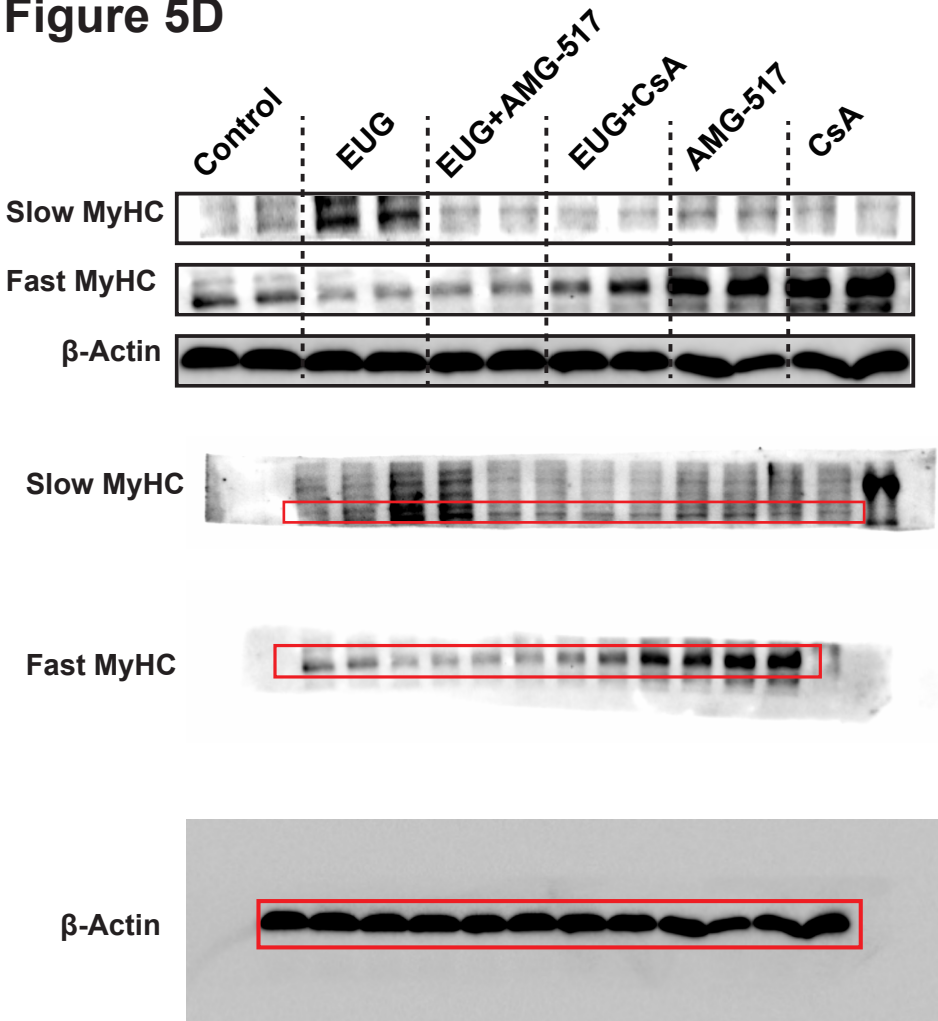

Supplement: Figure 5—source data 7. [file elife-90724-fig5-data7.pdf]

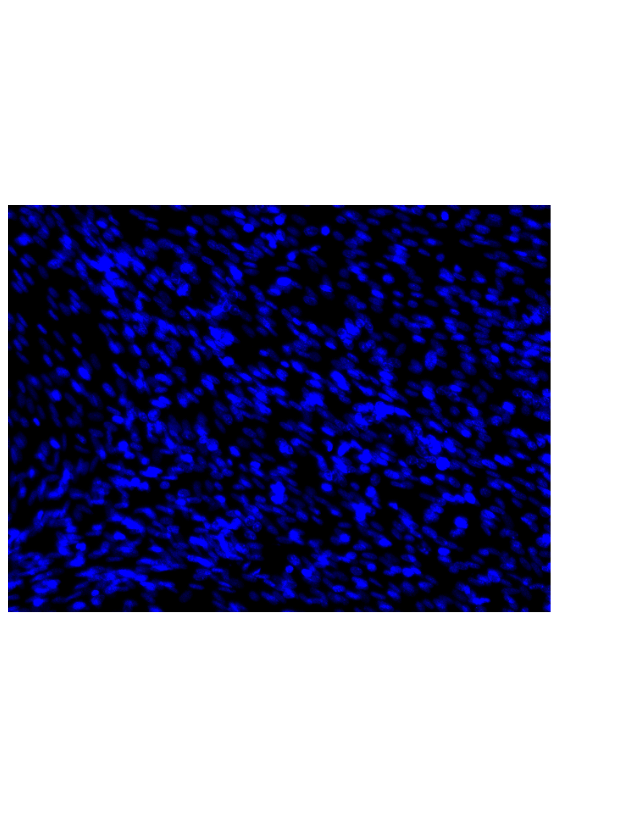

Supplement: Figure 5—source data 8. [file elife-90724-fig5-data8.zip › AMG-517/slow-AMG-dap.tif]

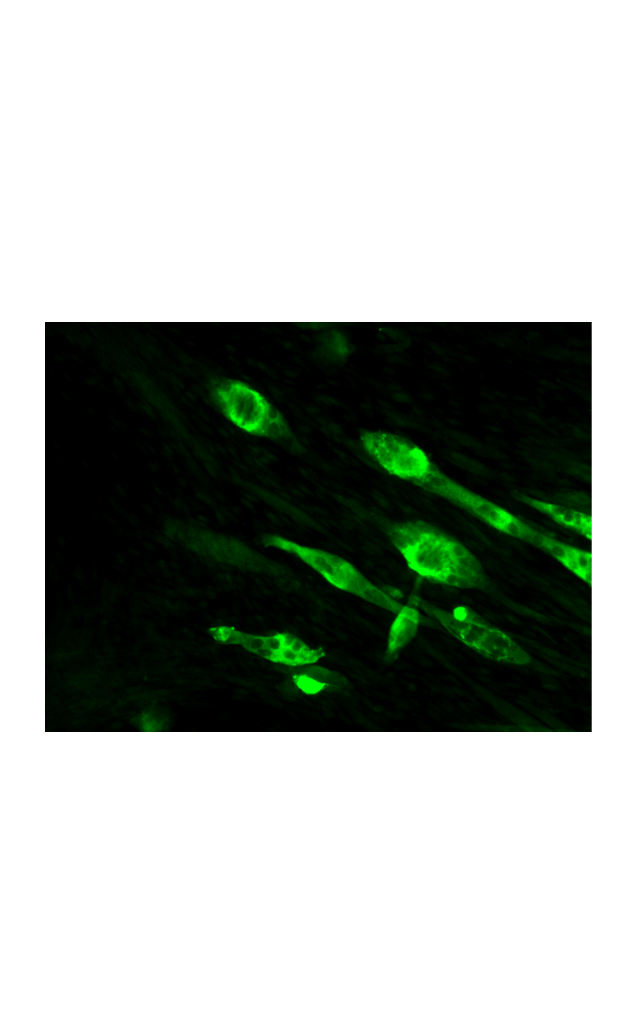

Supplement: Figure 5—source data 8. [file elife-90724-fig5-data8.zip › AMG-517/slow-AMG-l5.tif]

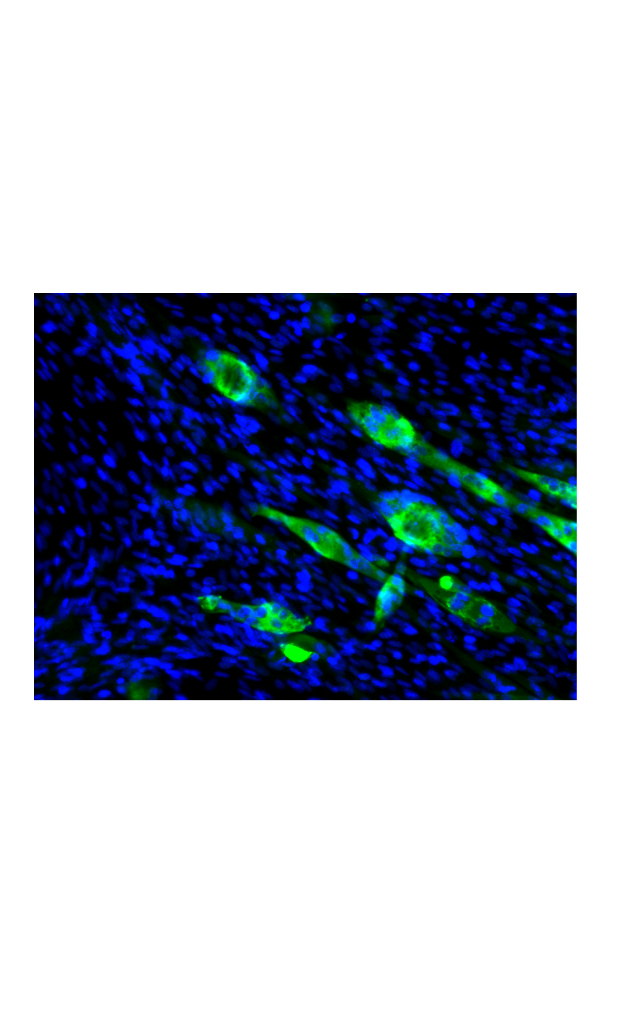

Supplement: Figure 5—source data 8. [file elife-90724-fig5-data8.zip › AMG-517/slow-AMG-║╧═╝.tif]

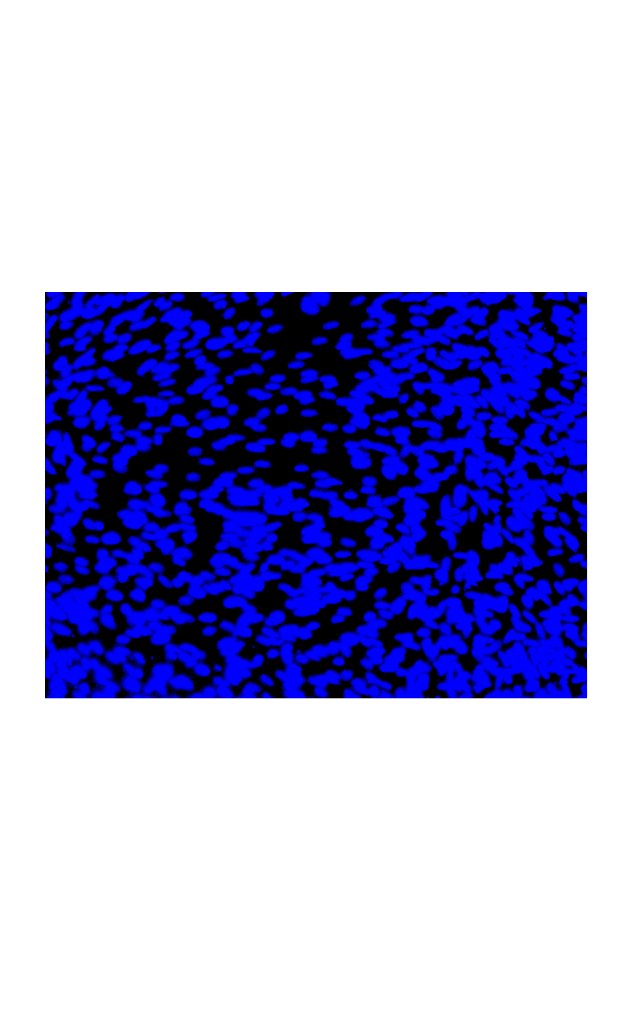

Supplement: Figure 5—source data 8. [file elife-90724-fig5-data8.zip › Control/DAPI.tif]

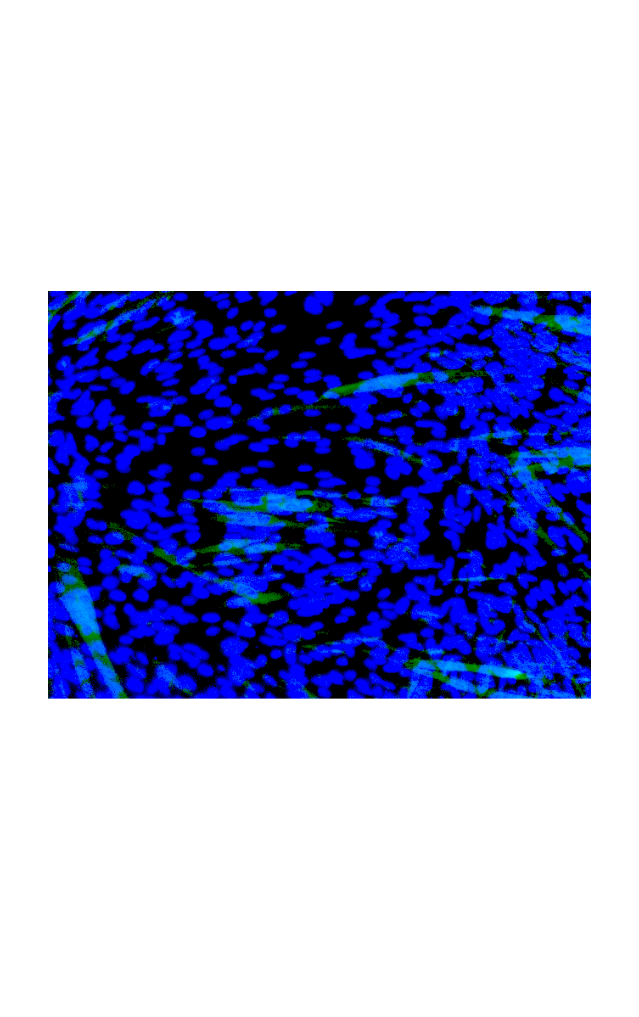

Supplement: Figure 5—source data 8. [file elife-90724-fig5-data8.zip › Control/Merge.tif]

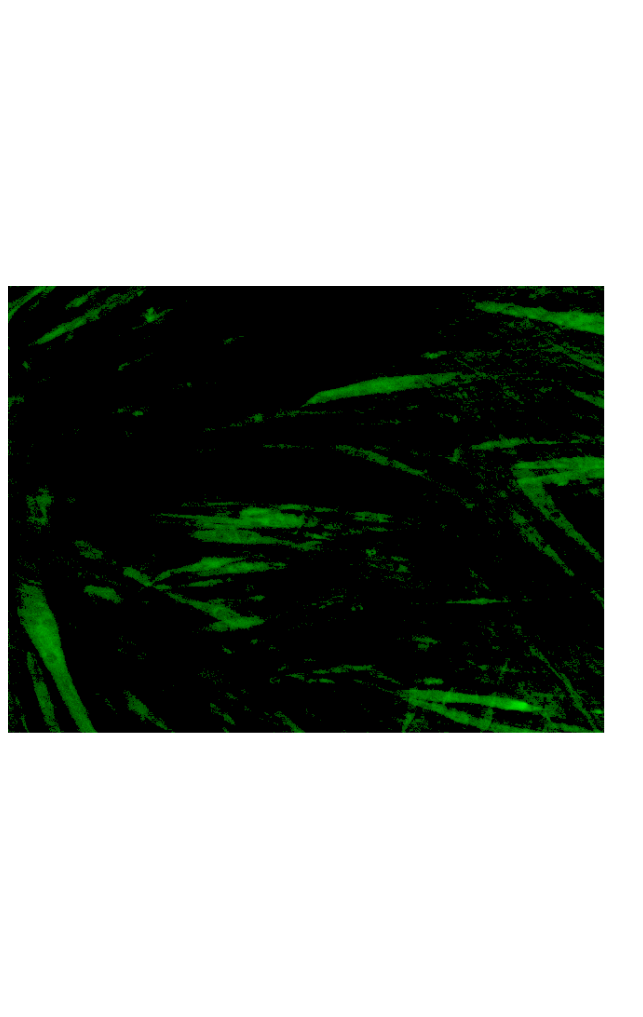

Supplement: Figure 5—source data 8. [file elife-90724-fig5-data8.zip › Control/Slow MyHC.tif]

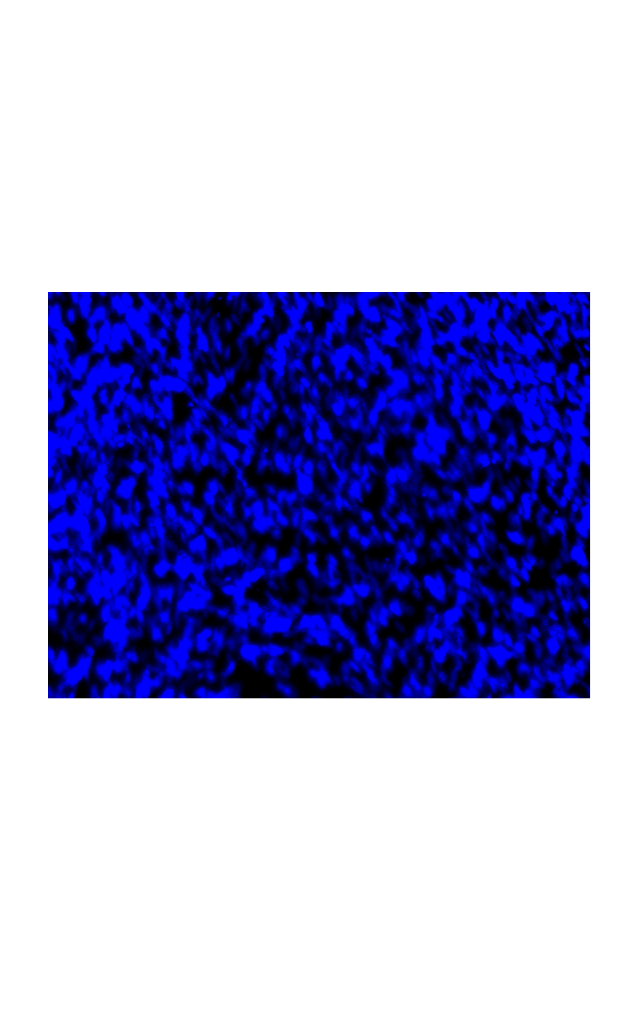

Supplement: Figure 5—source data 8. [file elife-90724-fig5-data8.zip › CsA/DAPI.tif]

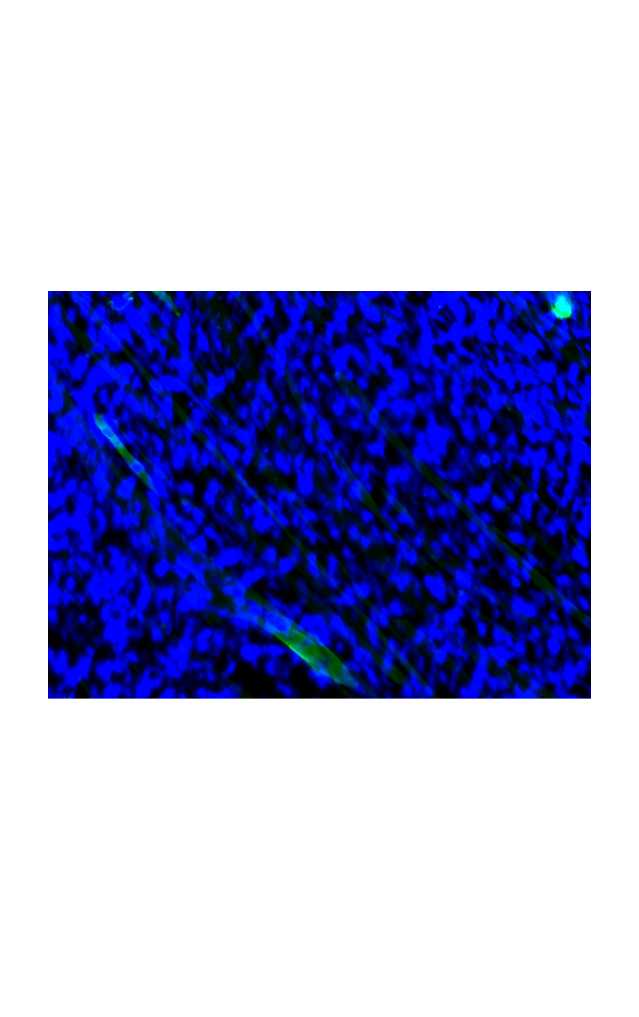

Supplement: Figure 5—source data 8. [file elife-90724-fig5-data8.zip › CsA/Merge.tif]

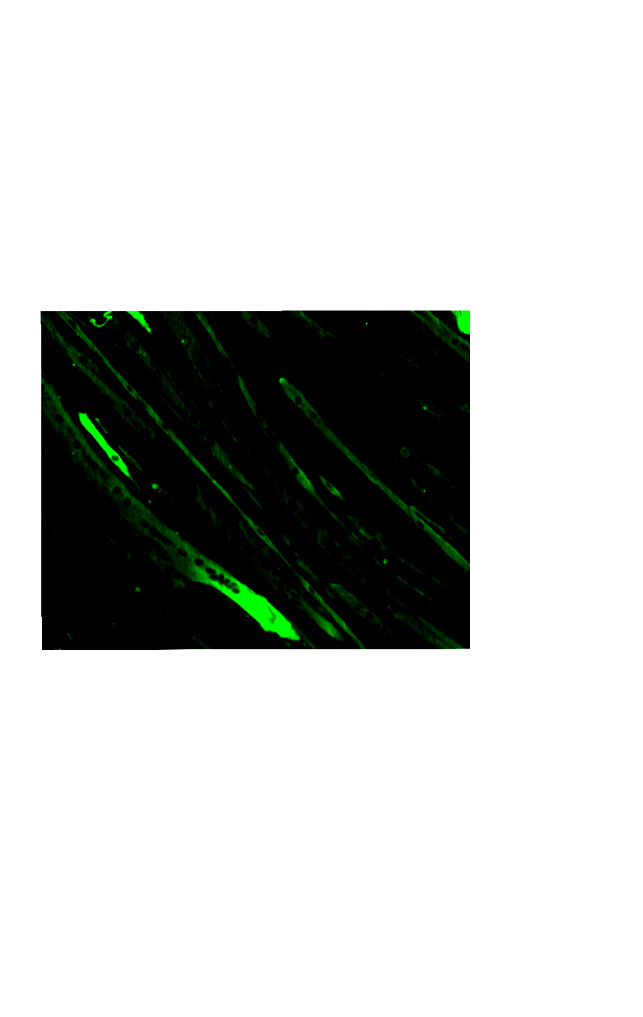

Supplement: Figure 5—source data 8. [file elife-90724-fig5-data8.zip › CsA/Slow MyHC.tif]

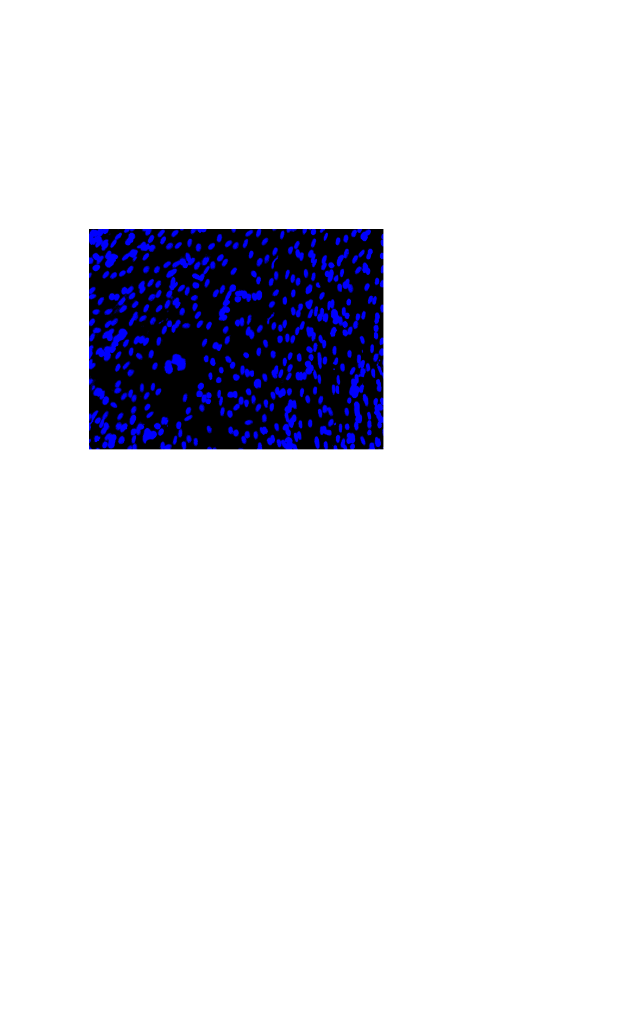

Supplement: Figure 5—source data 8. [file elife-90724-fig5-data8.zip › EUG+AMG-517/DAPI.tif]

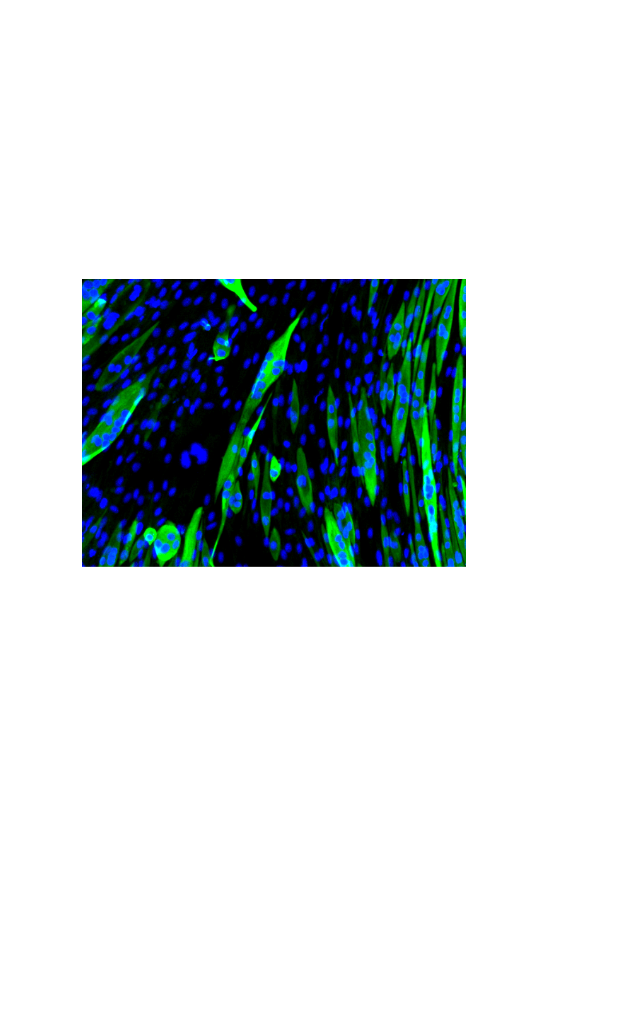

Supplement: Figure 5—source data 8. [file elife-90724-fig5-data8.zip › EUG+AMG-517/Merge.tif]

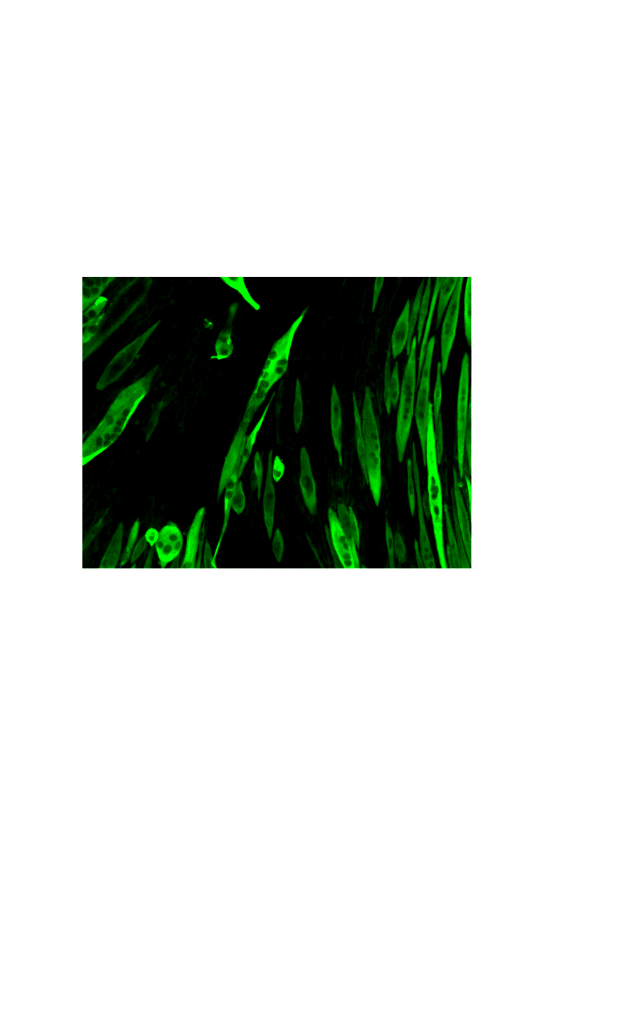

Supplement: Figure 5—source data 8. [file elife-90724-fig5-data8.zip › EUG+AMG-517/Slow MyHC.tif]

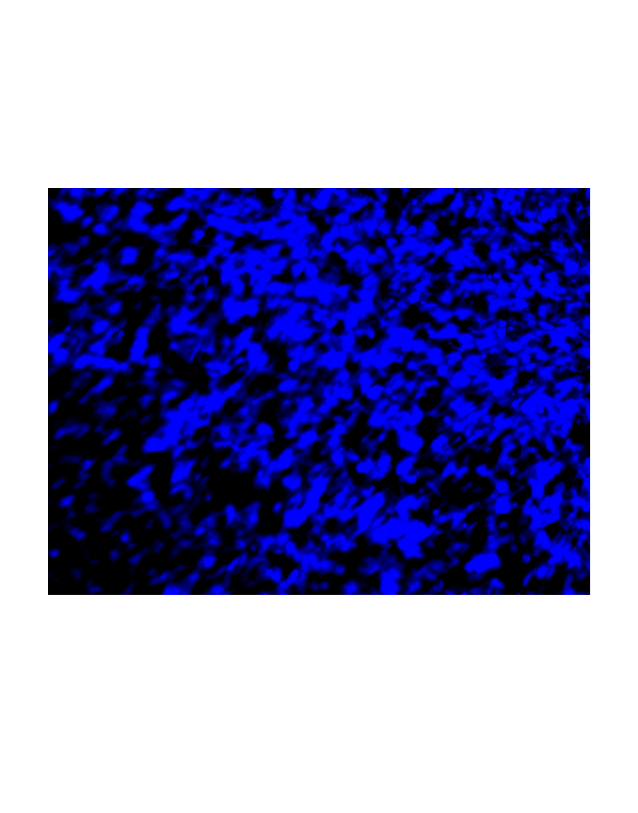

Supplement: Figure 5—source data 8. [file elife-90724-fig5-data8.zip › EUG+CsA/DAPI.tif]

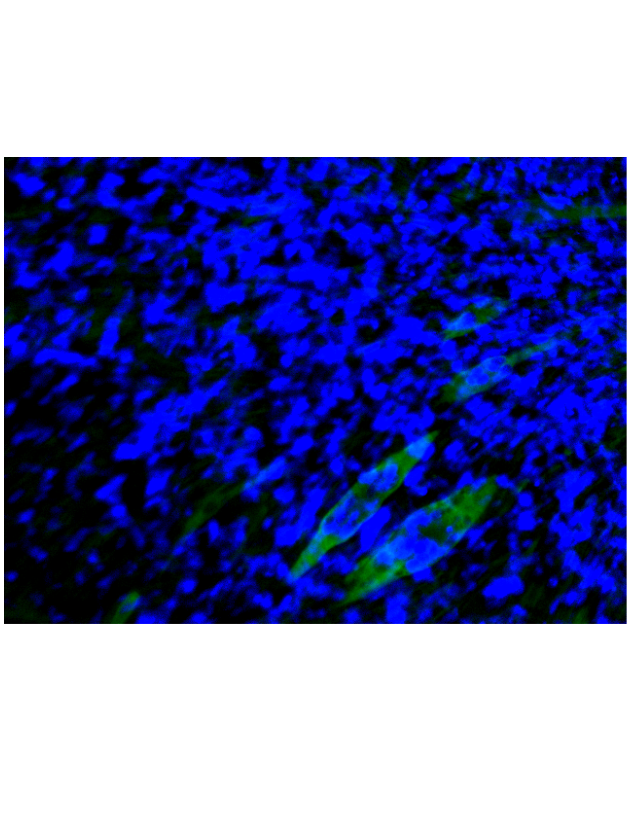

Supplement: Figure 5—source data 8. [file elife-90724-fig5-data8.zip › EUG+CsA/Merge.tif]

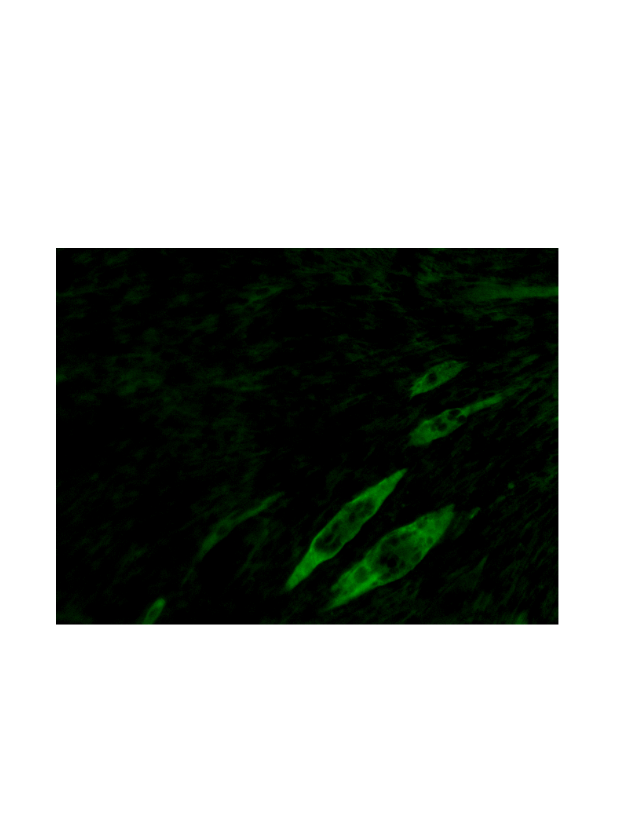

Supplement: Figure 5—source data 8. [file elife-90724-fig5-data8.zip › EUG+CsA/Slow MyHC.tif]

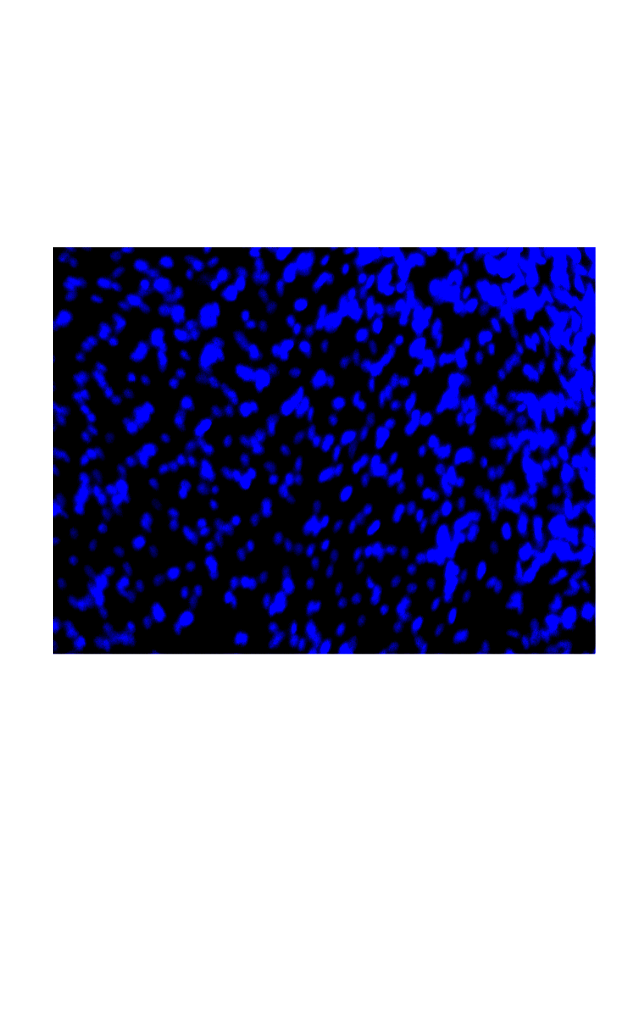

Supplement: Figure 5—source data 8. [file elife-90724-fig5-data8.zip › EUG/DAPI.tif]

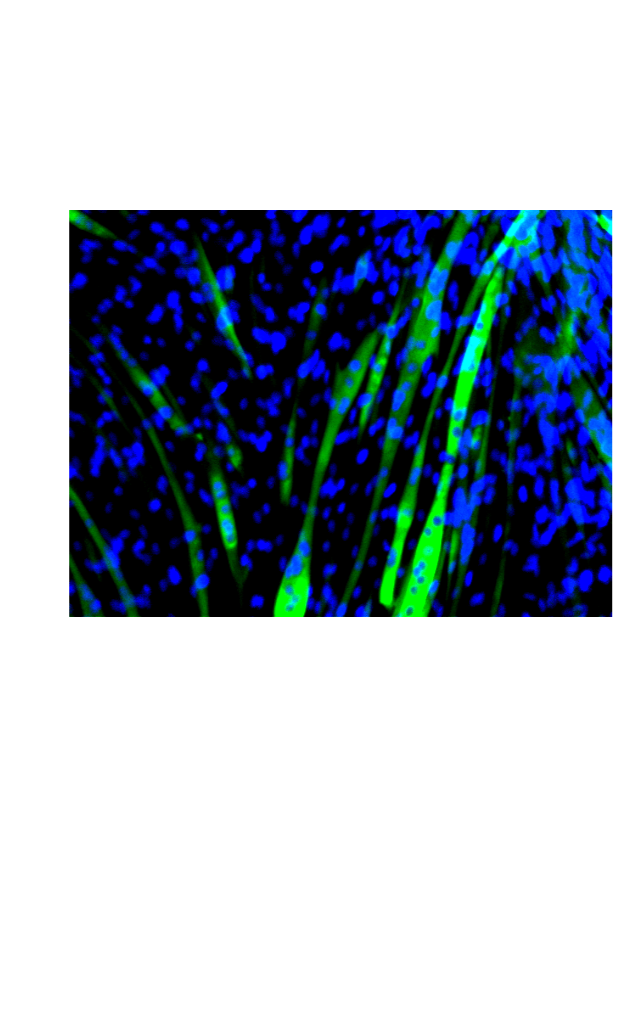

Supplement: Figure 5—source data 8. [file elife-90724-fig5-data8.zip › EUG/Merge.tif]

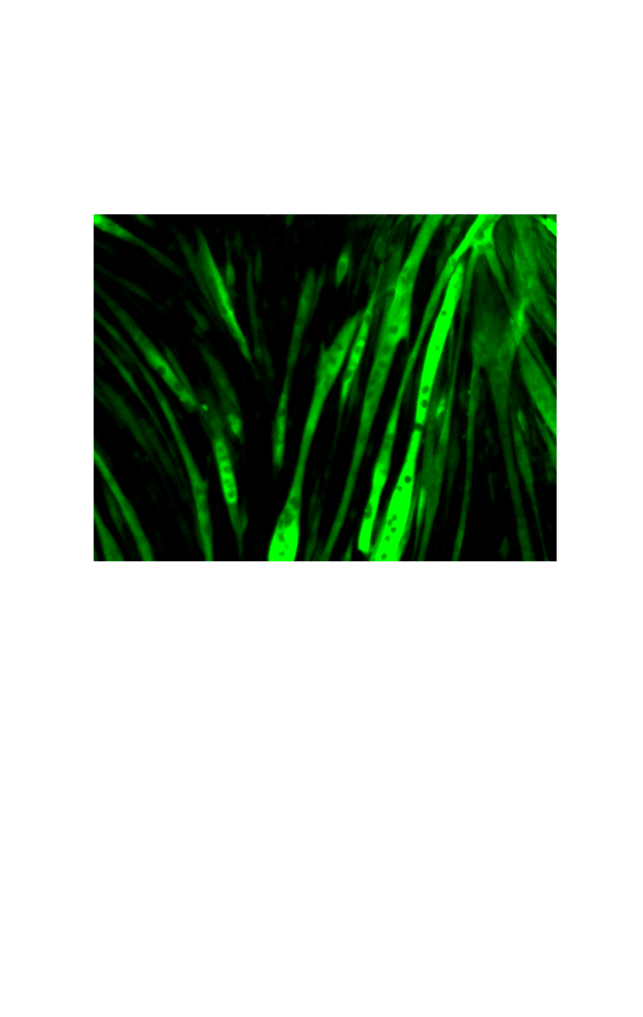

Supplement: Figure 5—source data 8. [file elife-90724-fig5-data8.zip › EUG/Slow MyHC.tif]

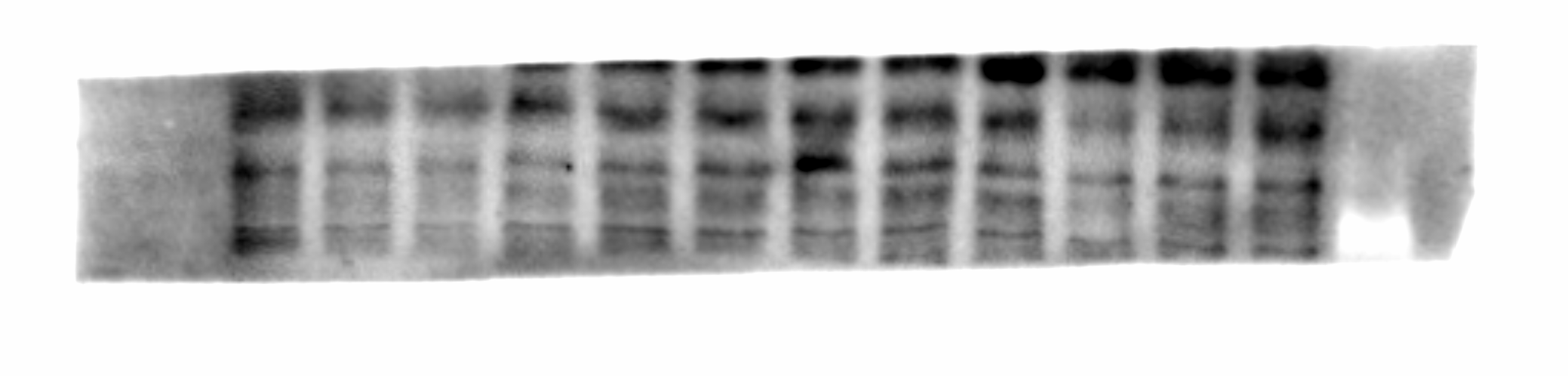

Supplement: Figure 6—source data 2. [file elife-90724-fig6-data2.zip › CnA.tif]

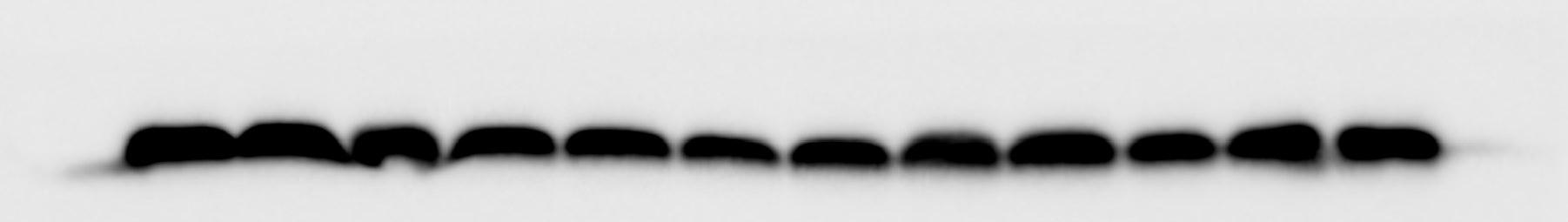

Supplement: Figure 6—source data 2. [file elife-90724-fig6-data2.zip › a┬-Actin.tif]

**Figure 6B**

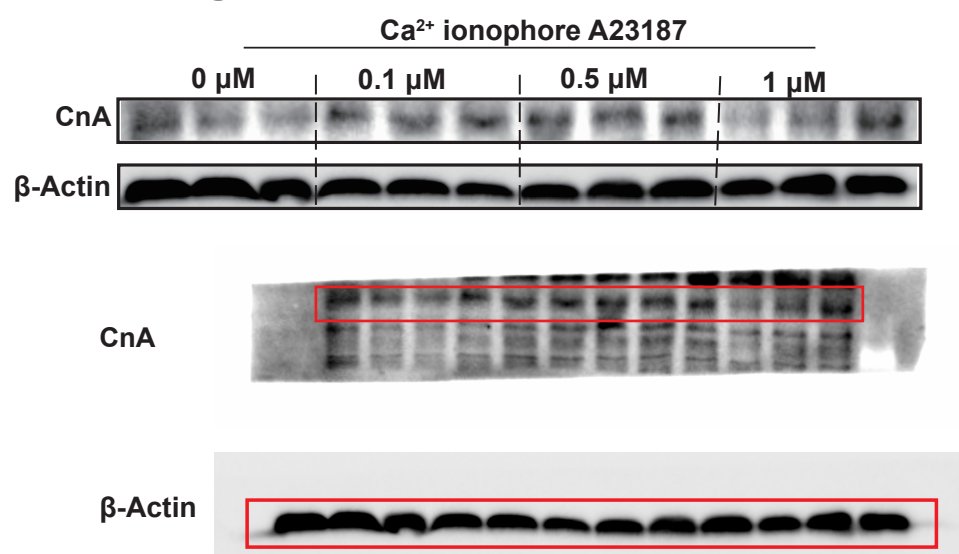

Supplement: Figure 6—source data 3. [file elife-90724-fig6-data3.pdf]

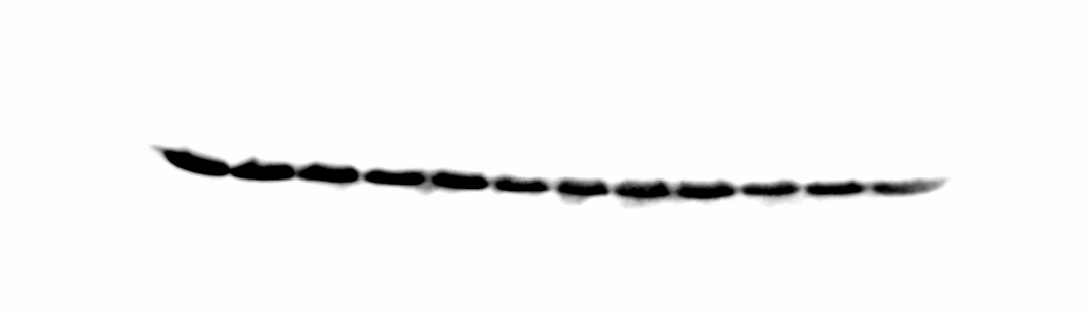

Supplement: Figure 7—source data 1. [file elife-90724-fig7-data1.zip › Actin.tif]

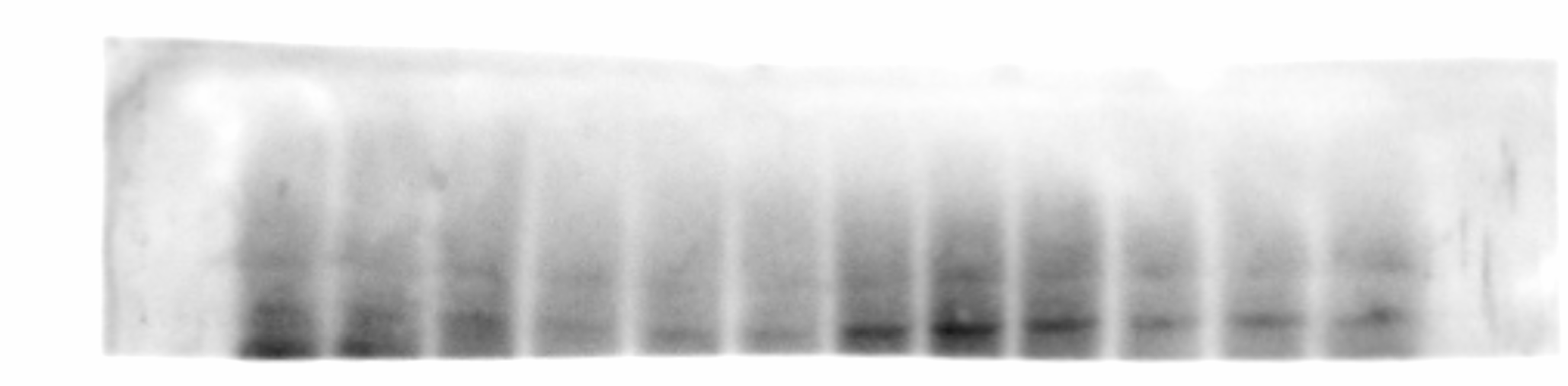

Supplement: Figure 7—source data 1. [file elife-90724-fig7-data1.zip › IL-15.tif]

Figure 7A

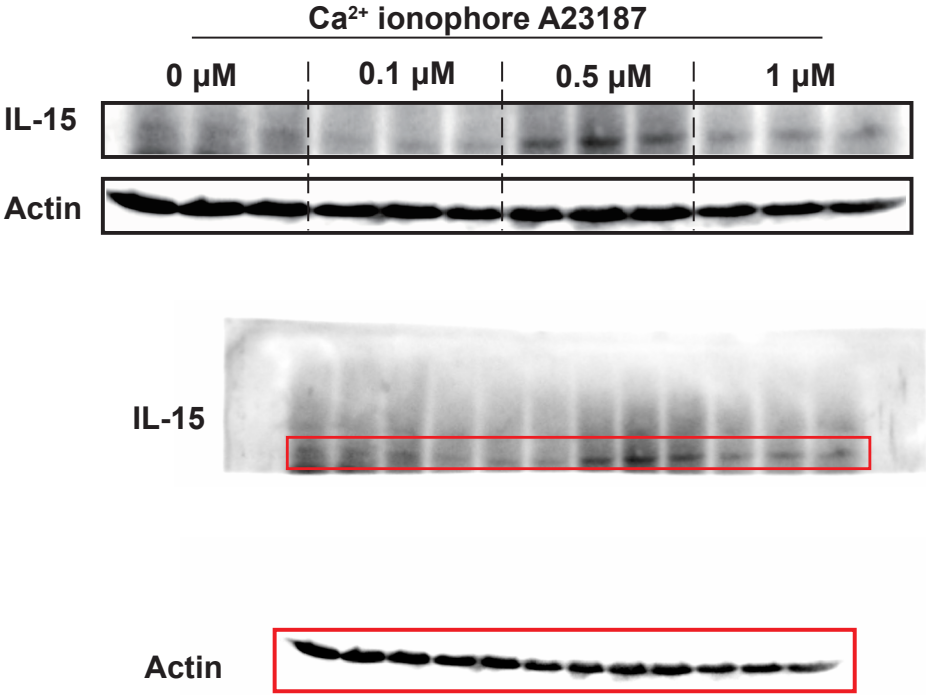

Supplement: Figure 7—source data 2. [file elife-90724-fig7-data2.pdf]

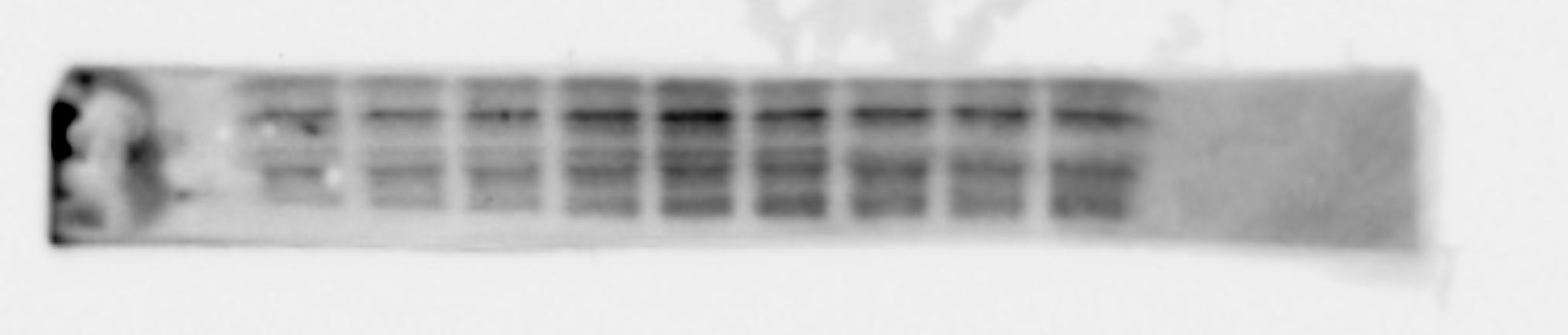

Supplement: Figure 7—source data 3. [file elife-90724-fig7-data3.zip › CnA.tif]
