# Supplementary figures and images for: Eugenol mimics exercise to promote skeletal muscle fiber remodeling and myokine IL-15 expression by activating TRPV1 channel (part 2 of 2)
Source: eLife. 2024 Jun 24;12:RP90724. doi: 10.7554/eLife.90724 (PMC11196110; doi:10.7554/eLife.90724)

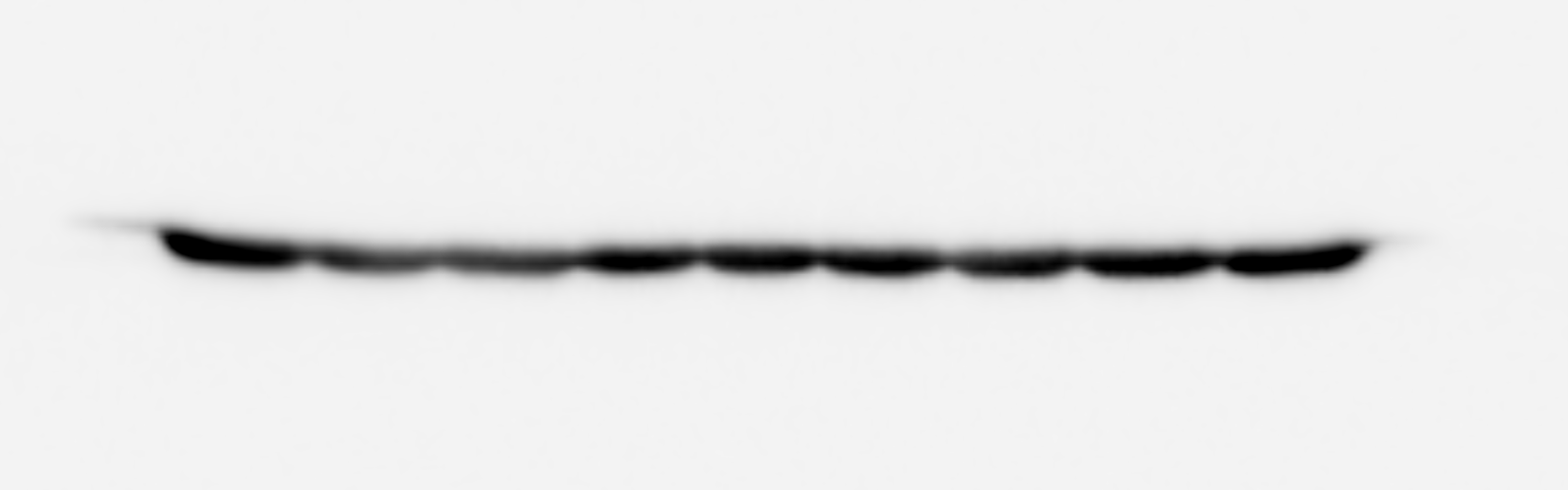

Supplement: Figure 7—source data 3. [file elife-90724-fig7-data3.zip › a┬-Actin.tif]

**Figure 7B**

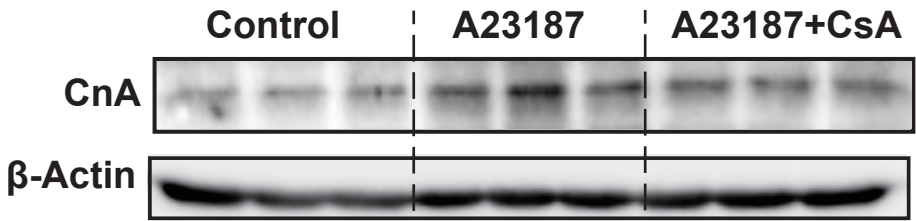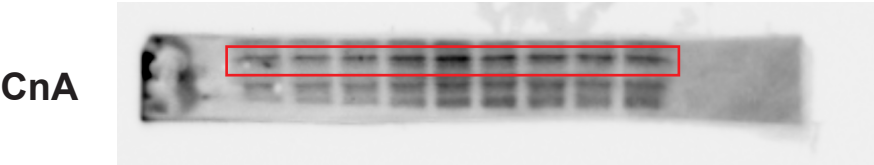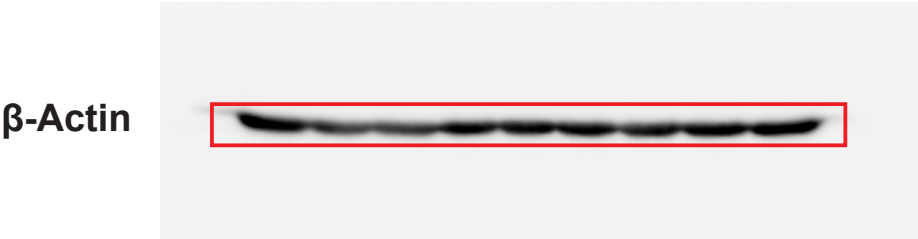

Supplement: Figure 7—source data 4. [file elife-90724-fig7-data4.pdf]

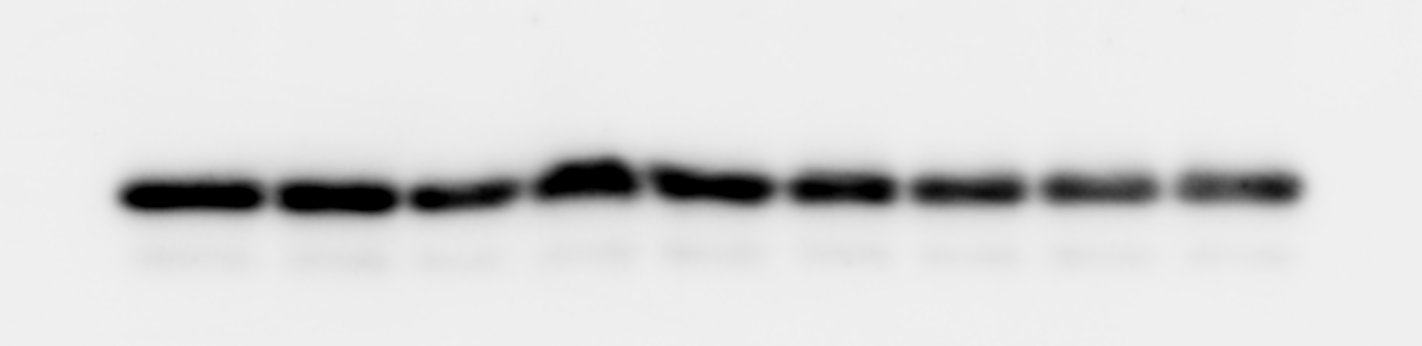

Supplement: Figure 7—source data 5. [file elife-90724-fig7-data5.zip › Histone H3.tif]

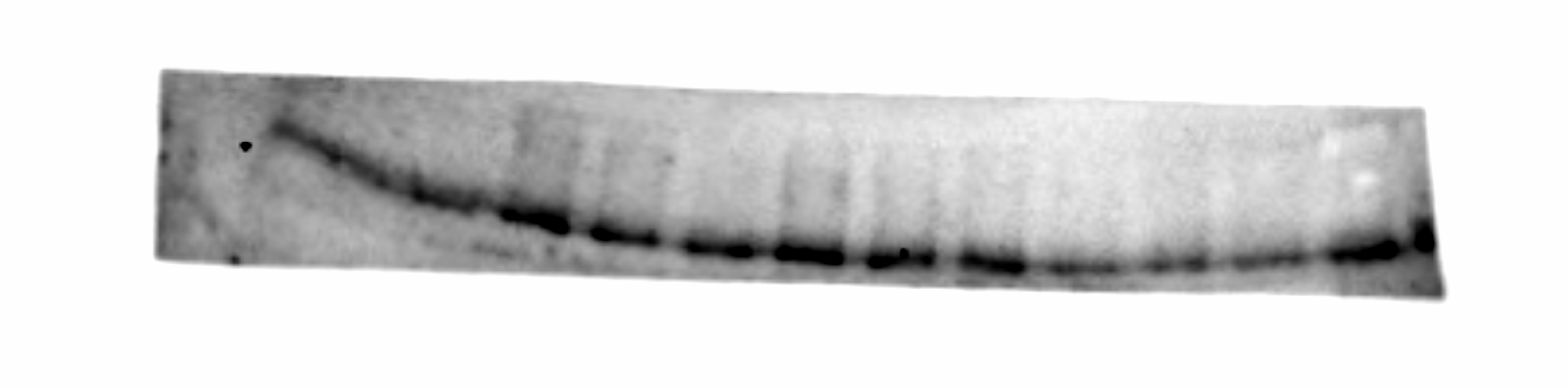

Supplement: Figure 7—source data 5. [file elife-90724-fig7-data5.zip › NFATc1.tif]

**Figure 7C**

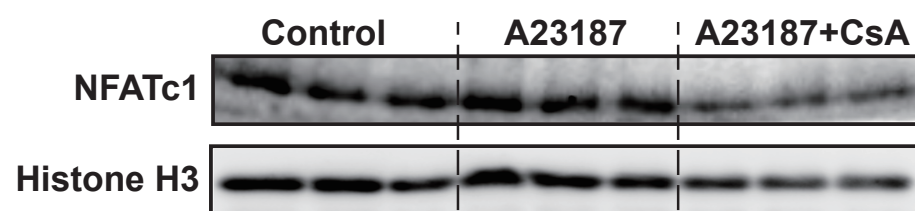

NFATc1

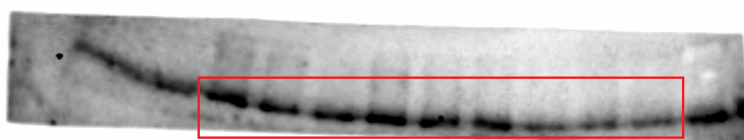

Histone H3

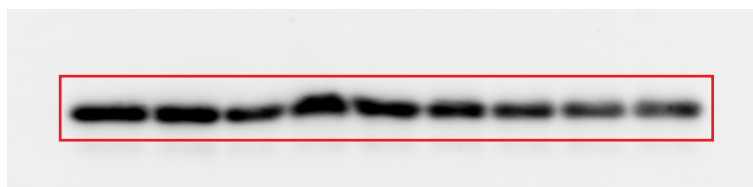

Supplement: Figure 7—source data 6. [file elife-90724-fig7-data6.pdf]

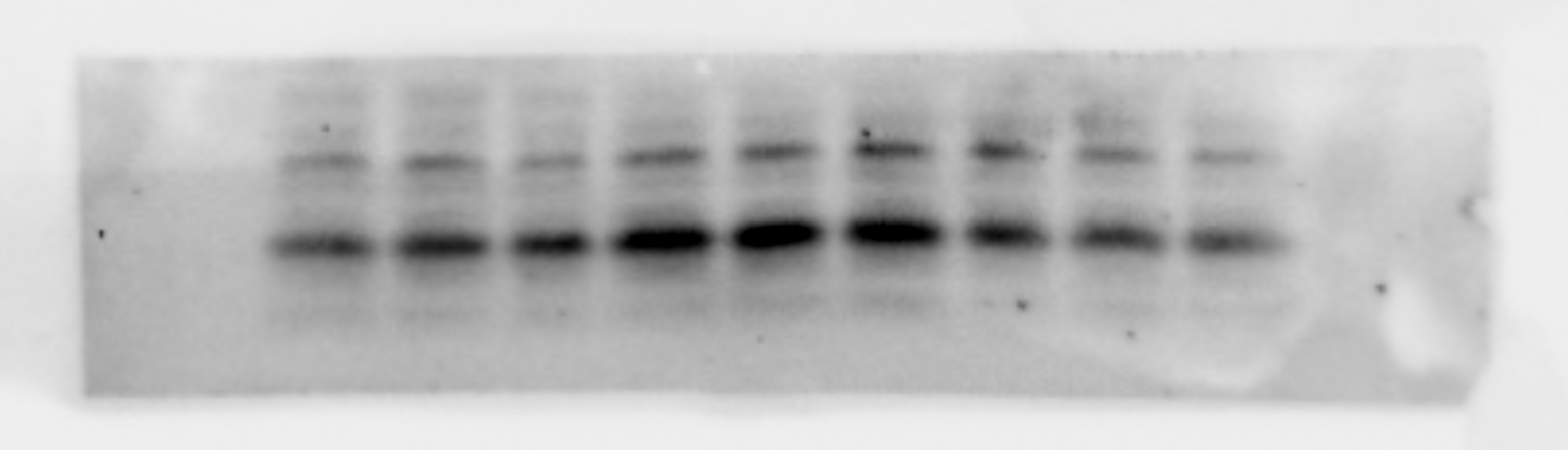

Supplement: Figure 7—source data 7. [file elife-90724-fig7-data7.zip › IL-15.tif]

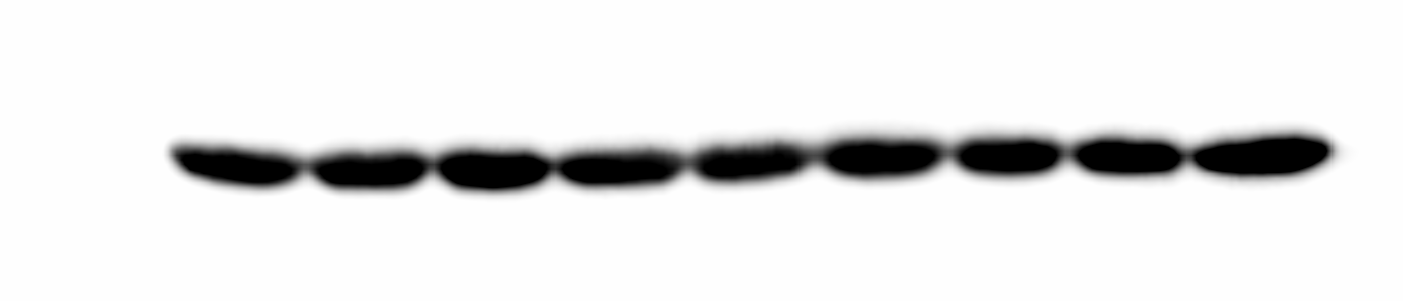

Supplement: Figure 7—source data 7. [file elife-90724-fig7-data7.zip › a┬-Actin.tif]

Figure 7D

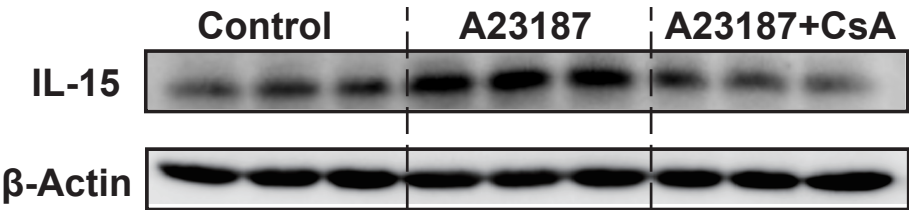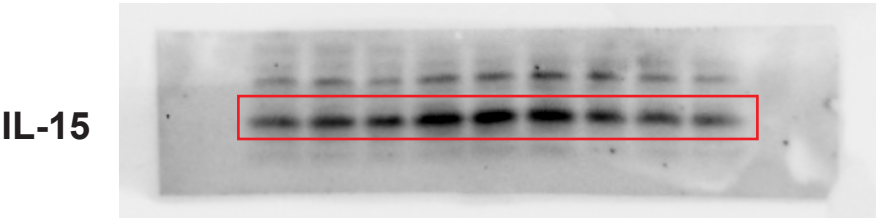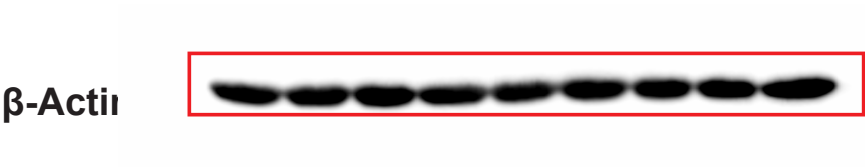

Supplement: Figure 7—source data 8. [file elife-90724-fig7-data8.pdf]

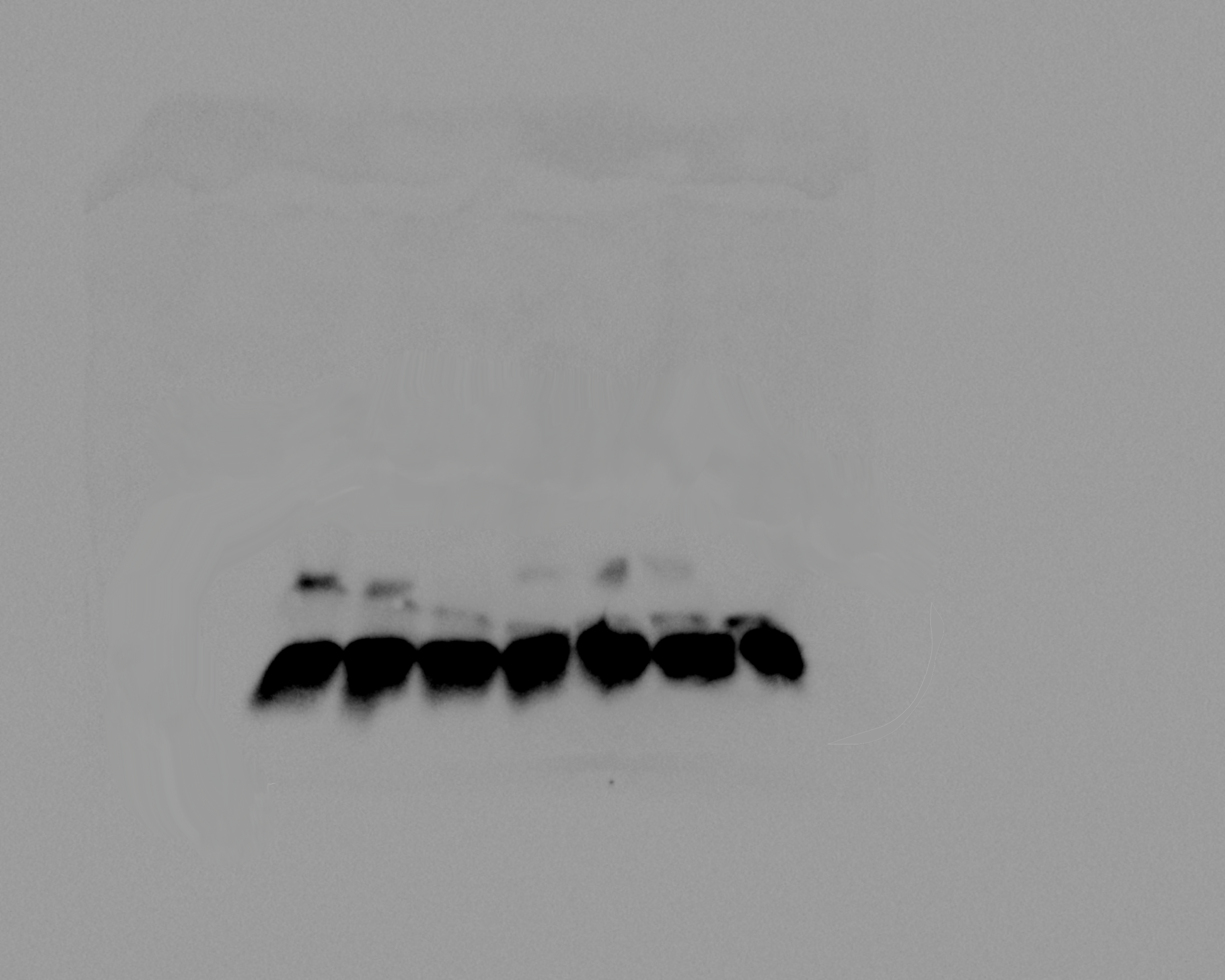

Supplement: Figure 7—source data 9. [file elife-90724-fig7-data9.zip › EMSA.tif]

Figure 7H

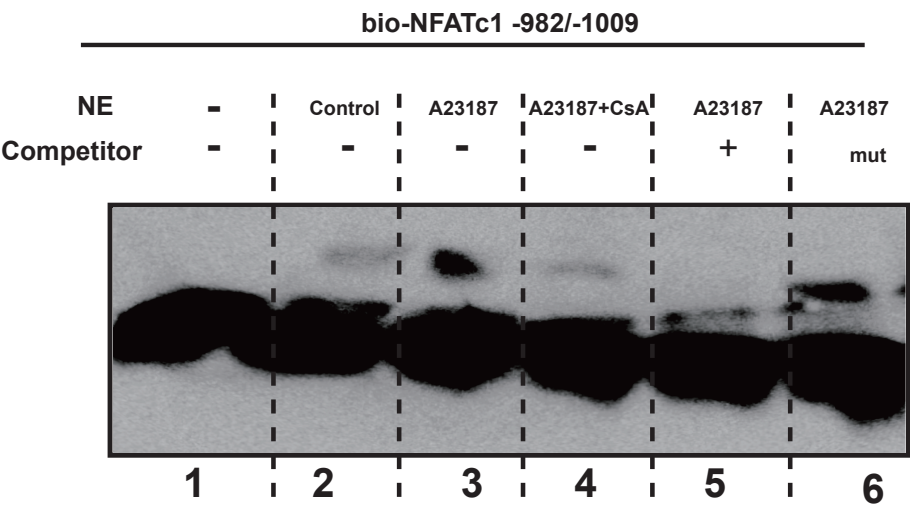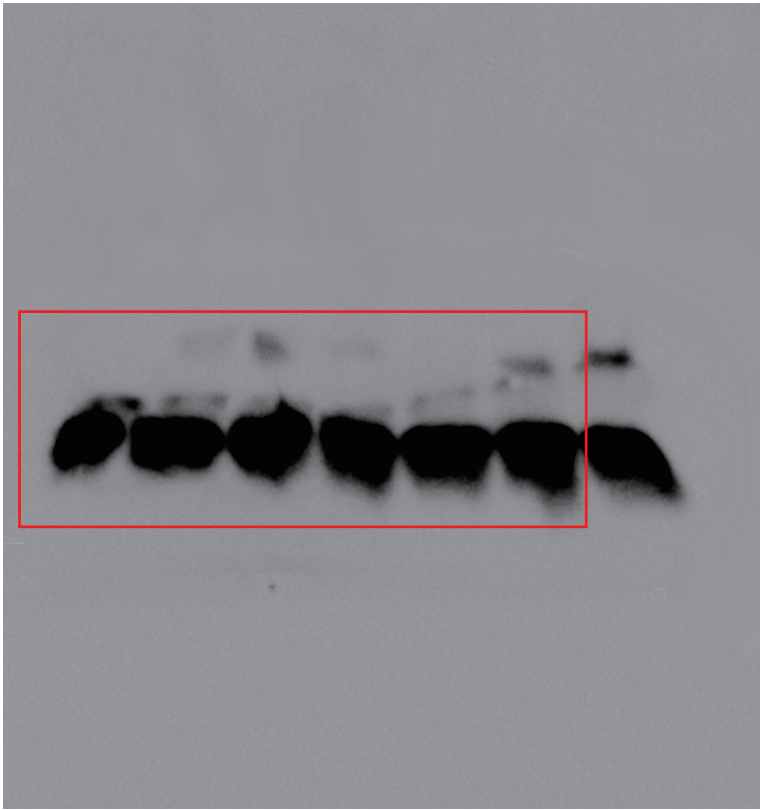

Supplement: Figure 7—source data 10. [file elife-90724-fig7-data10.pdf]

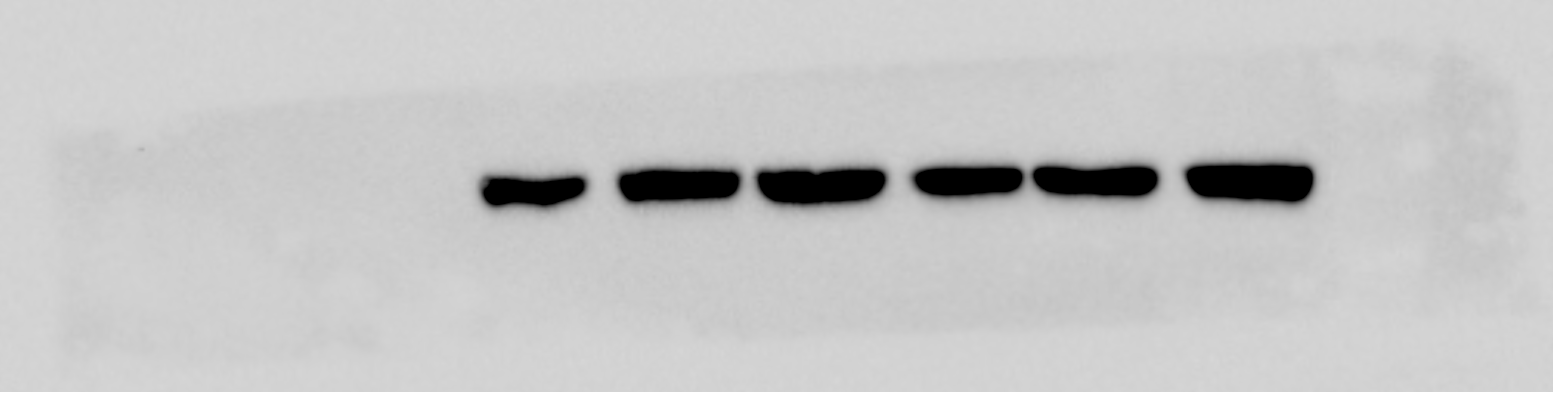

Supplement: Figure 7—source data 11. [file elife-90724-fig7-data11.zip › NFATc1.tif]

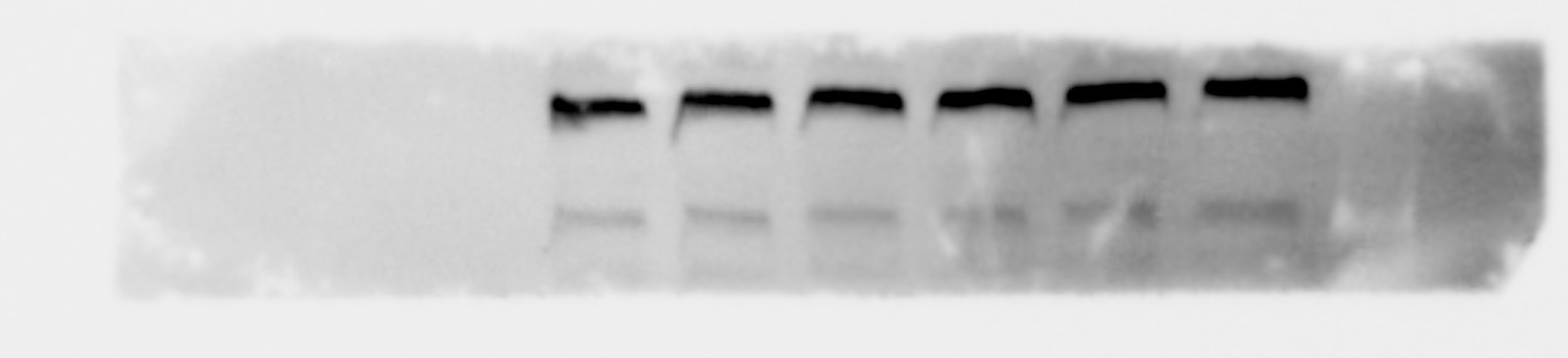

Supplement: Figure 7—source data 11. [file elife-90724-fig7-data11.zip › a┬-Actin.tif]

**Figure 7I**

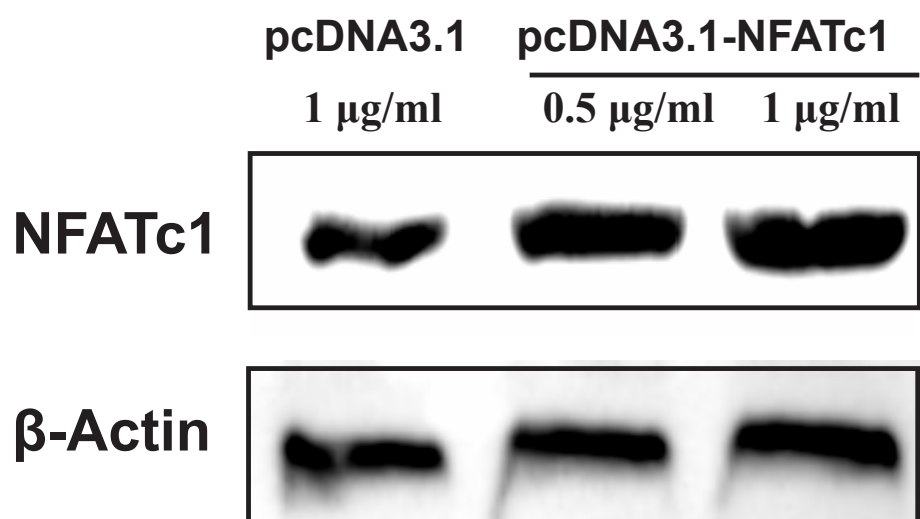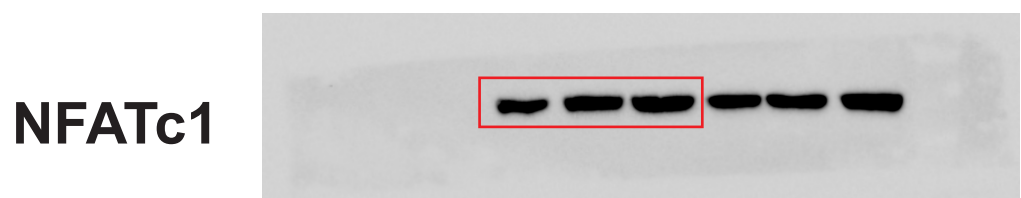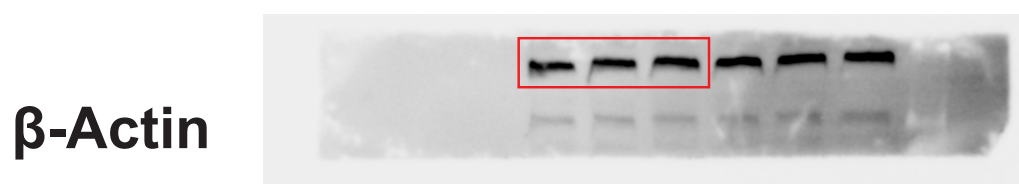

Supplement: Figure 7—source data 12. [file elife-90724-fig7-data12.pdf]

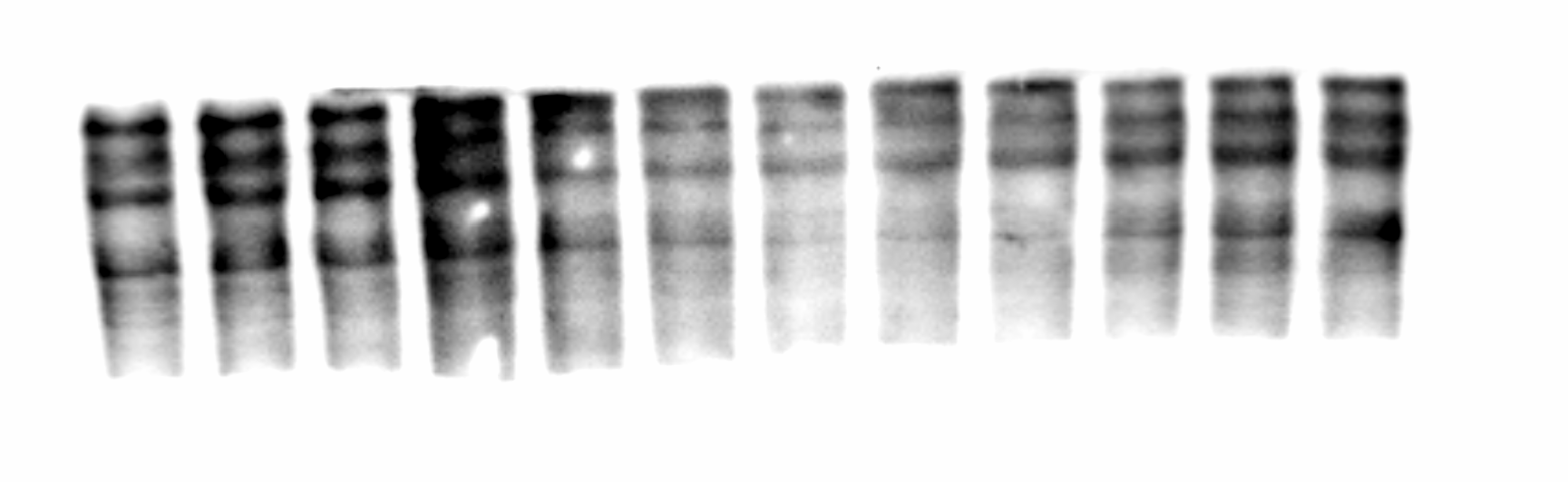

Supplement: Figure 7—figure supplement 1—source data 1. [file elife-90724-fig7-figsupp1-data1.zip › Fast MyHC.tif]

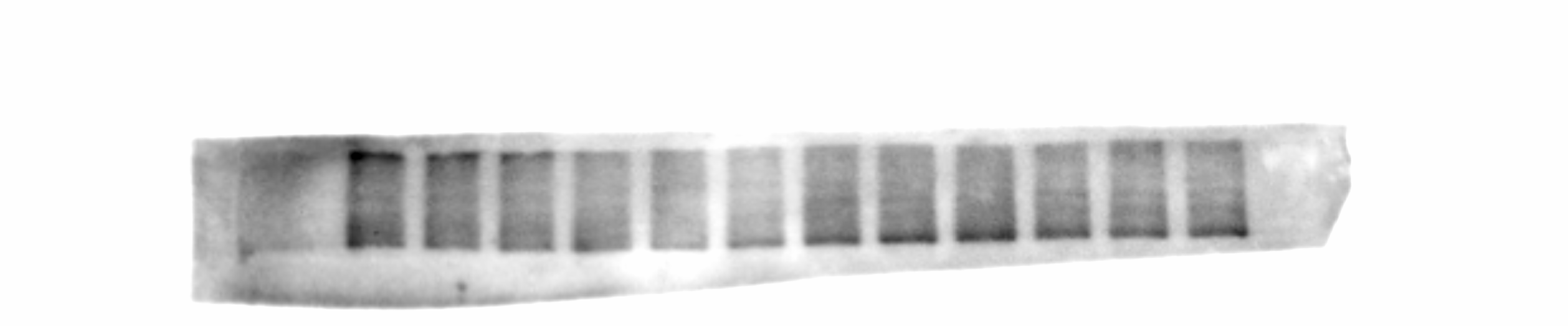

Supplement: Figure 7—figure supplement 1—source data 1. [file elife-90724-fig7-figsupp1-data1.zip › Slow MyHC.tif]

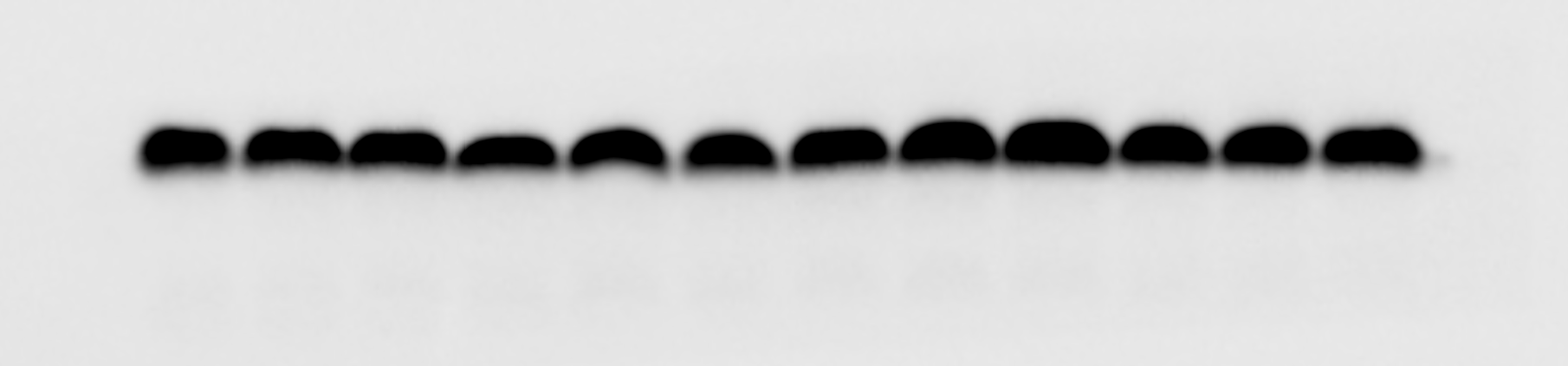

Supplement: Figure 7—figure supplement 1—source data 1. [file elife-90724-fig7-figsupp1-data1.zip › a┬-Actin.tif]

**Figure 7-figure supplement 1A**

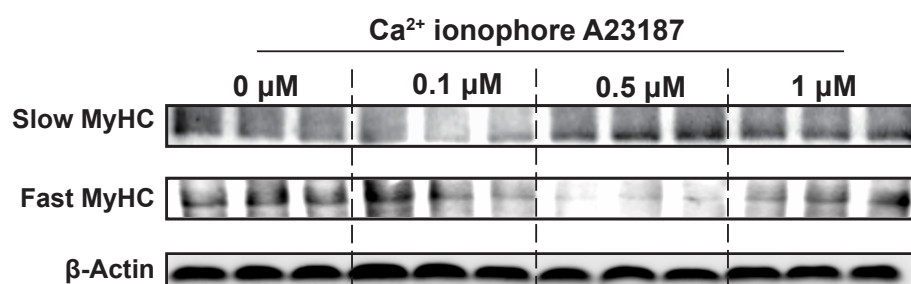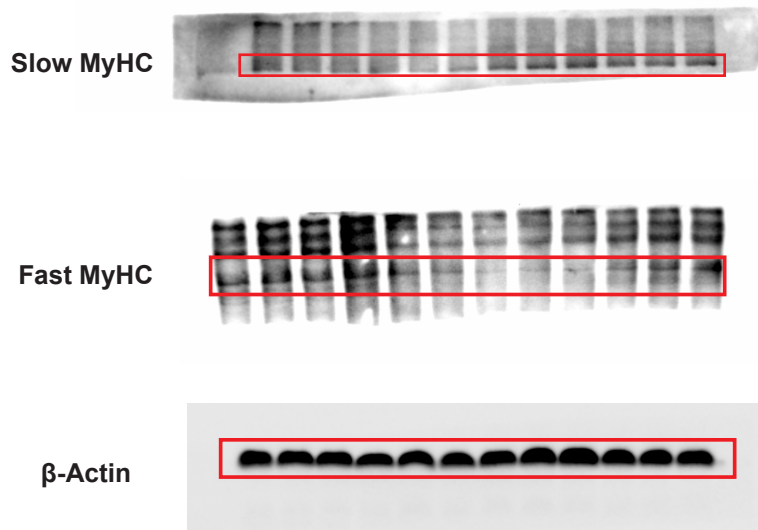

Supplement: Figure 7—figure supplement 1—source data 2. [file elife-90724-fig7-figsupp1-data2.pdf]

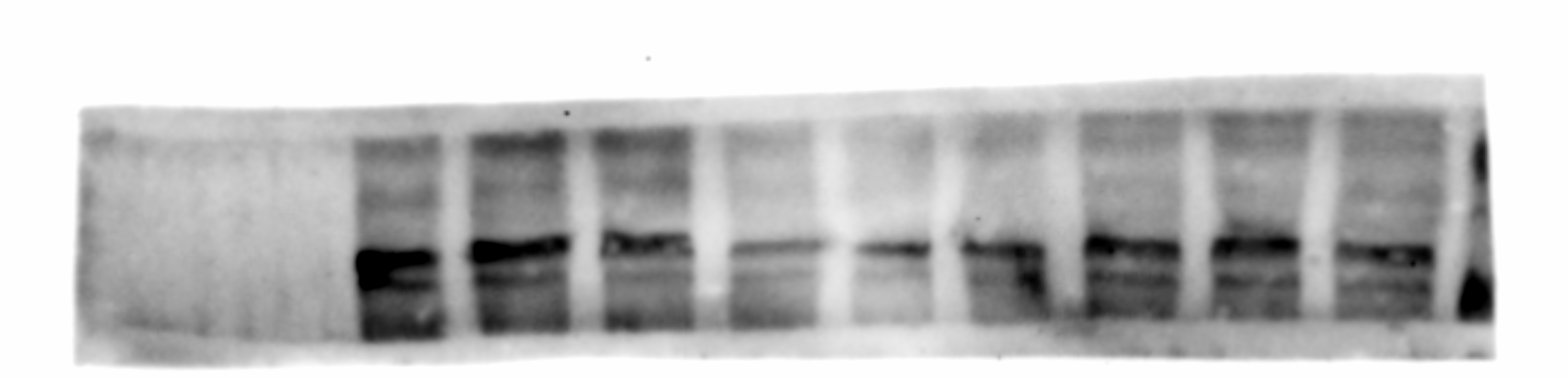

Supplement: Figure 7—figure supplement 1—source data 4. [file elife-90724-fig7-figsupp1-data4.zip › Fast MyHC.tif]

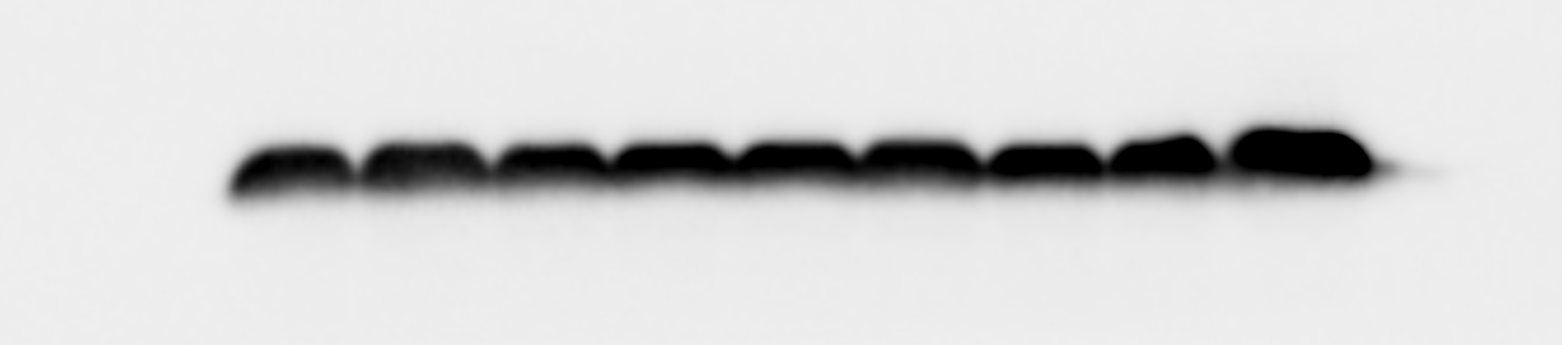

Supplement: Figure 7—figure supplement 1—source data 4. [file elife-90724-fig7-figsupp1-data4.zip › htt 2021-05-02 actin-slow 15 20 15.tif]

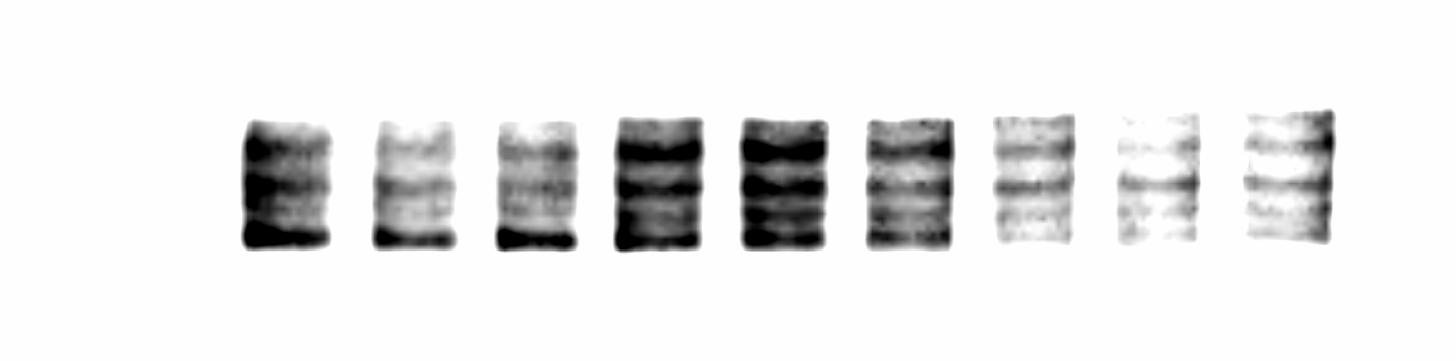

Supplement: Figure 7—figure supplement 1—source data 4. [file elife-90724-fig7-figsupp1-data4.zip › Slow MyHC.tif]

**Figure 7-figure supplement 1C**

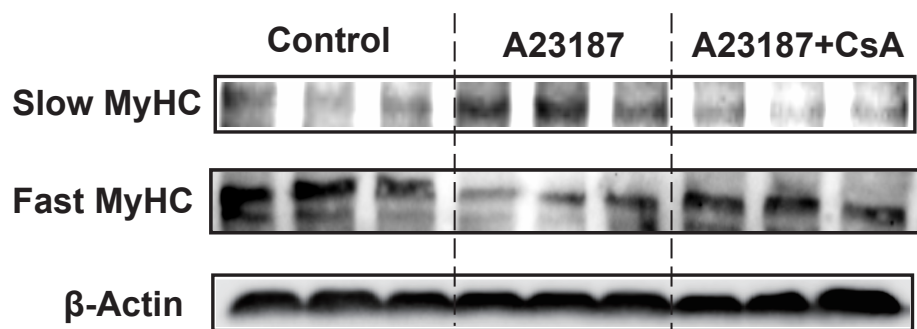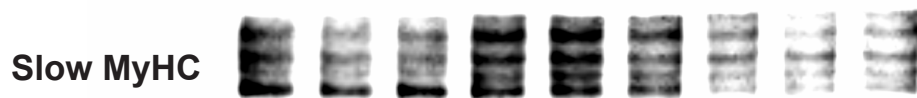

Fast MyHC

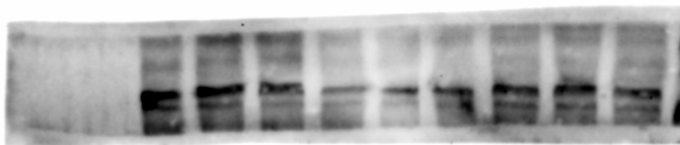

$\beta$ -Actin

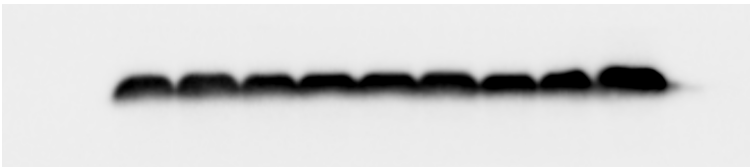

Supplement: Figure 7—figure supplement 1—source data 5. [file elife-90724-fig7-figsupp1-data5.pdf]

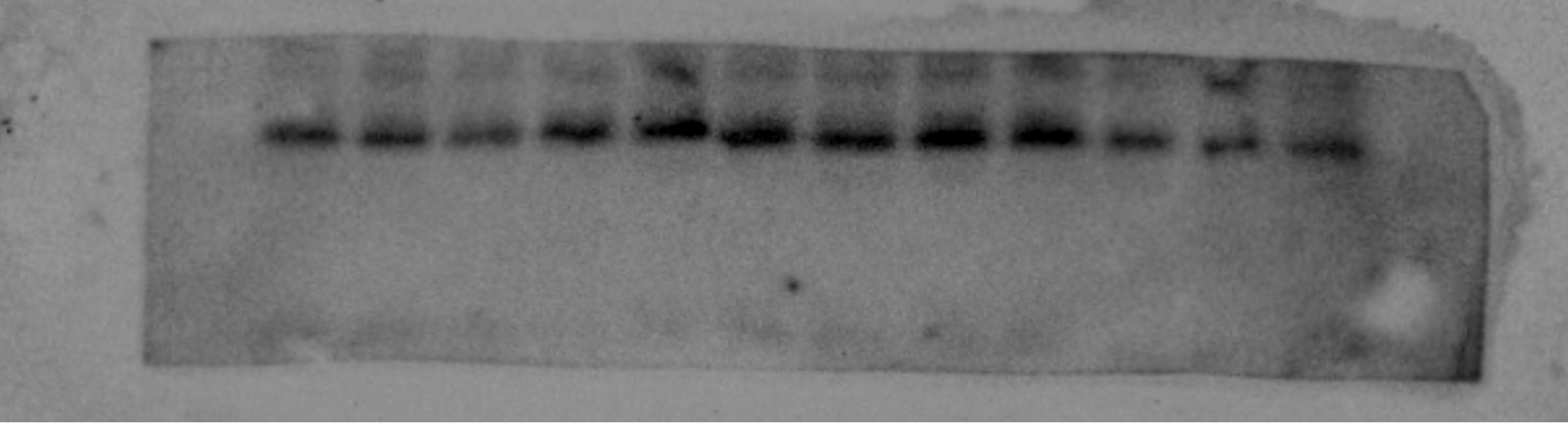

Supplement: Figure 8—source data 2. [file elife-90724-fig8-data2.zip › IL-15.tif]

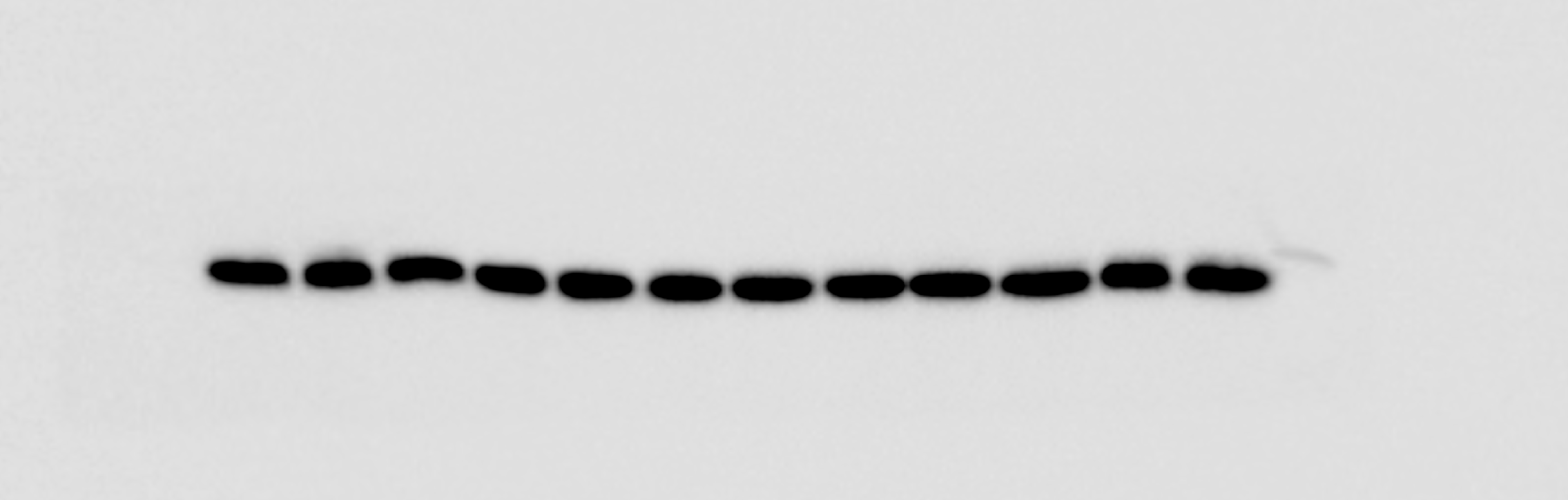

Supplement: Figure 8—source data 2. [file elife-90724-fig8-data2.zip › a┬-Actin.tif]

Figure 8B

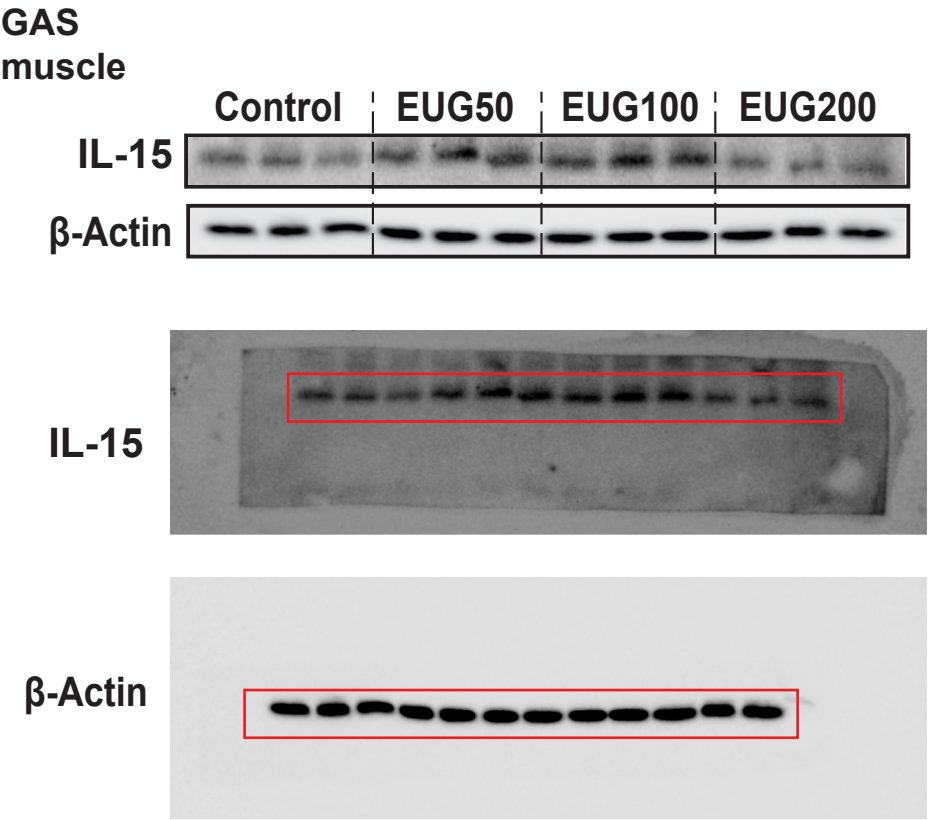

Supplement: Figure 8—source data 3. [file elife-90724-fig8-data3.pdf]

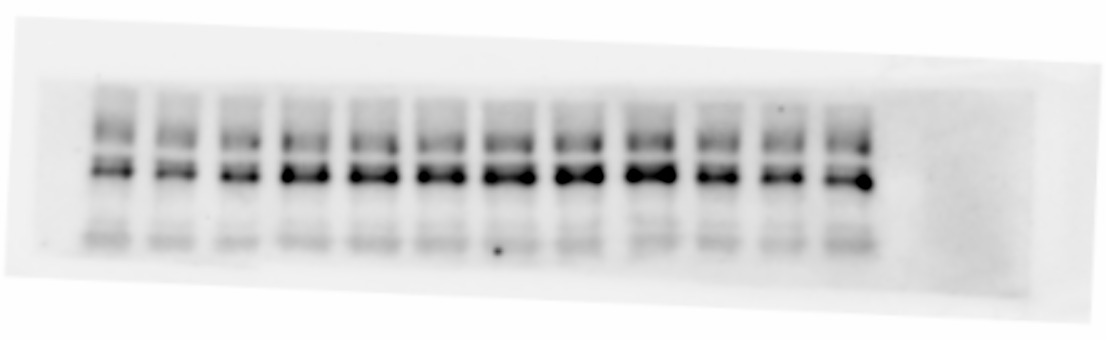

Supplement: Figure 8—source data 4. [file elife-90724-fig8-data4.zip › IL-15.tif]

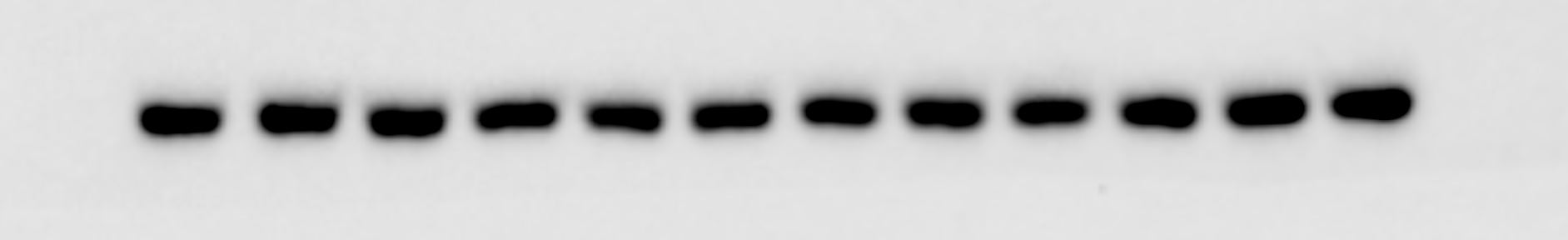

Supplement: Figure 8—source data 4. [file elife-90724-fig8-data4.zip › a┬-Actin.tif]

Figure 8C

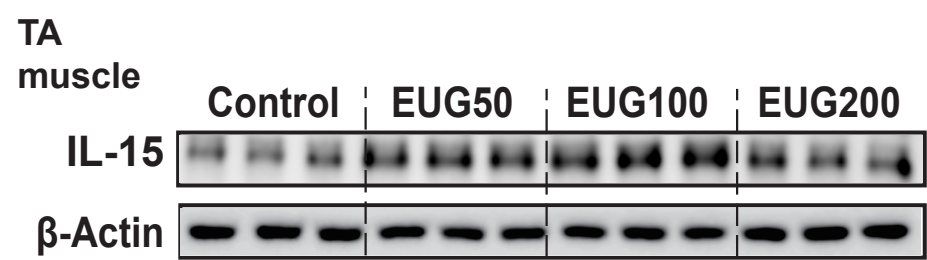

IL-15

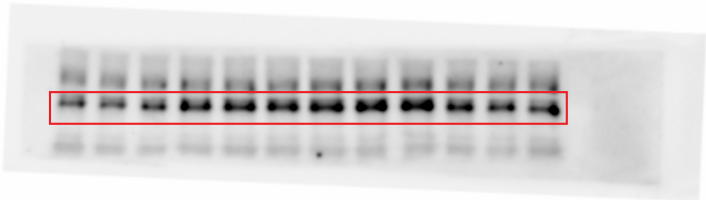

$\beta$ -Actin

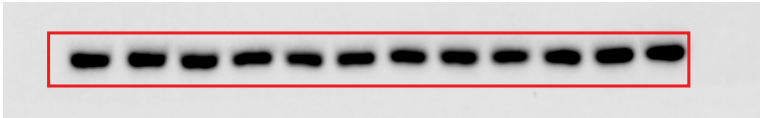

Supplement: Figure 8—source data 5. [file elife-90724-fig8-data5.pdf]

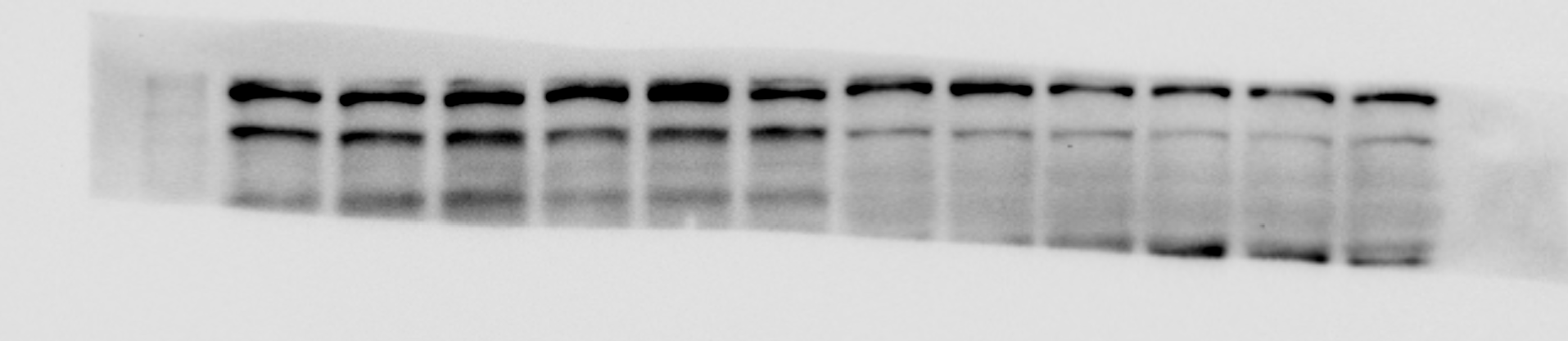

Supplement: Figure 8—source data 7. [file elife-90724-fig8-data7.zip › Fast MyHC.tif]

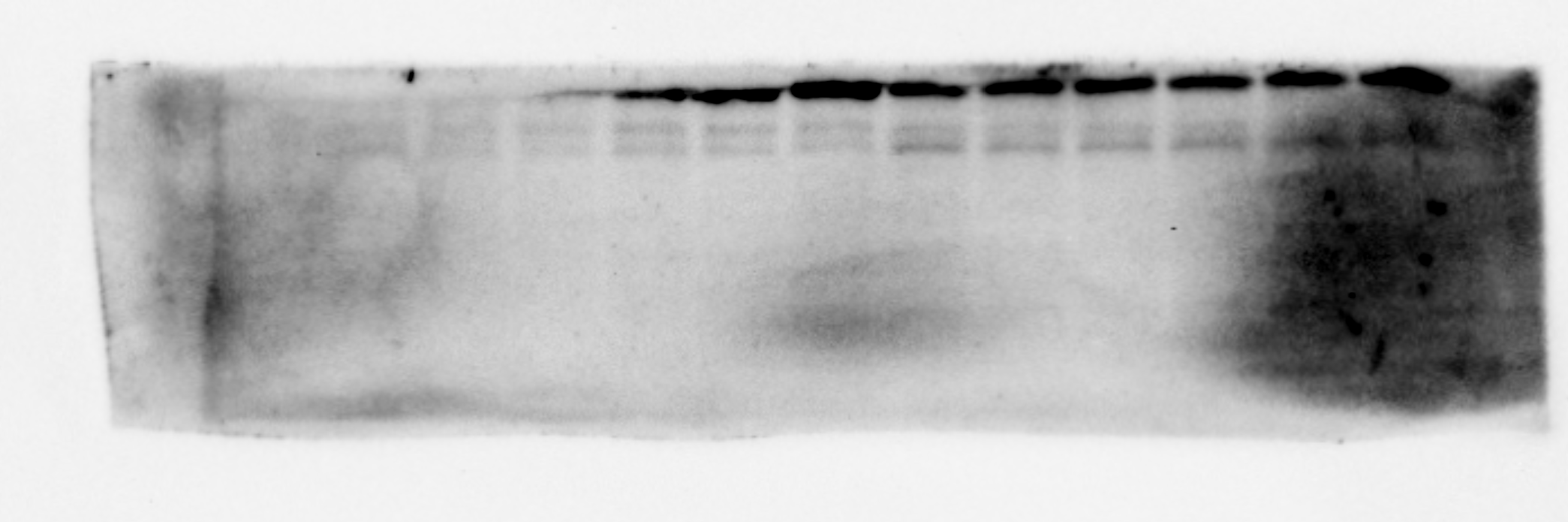

Supplement: Figure 8—source data 7. [file elife-90724-fig8-data7.zip › IL-15.tif]

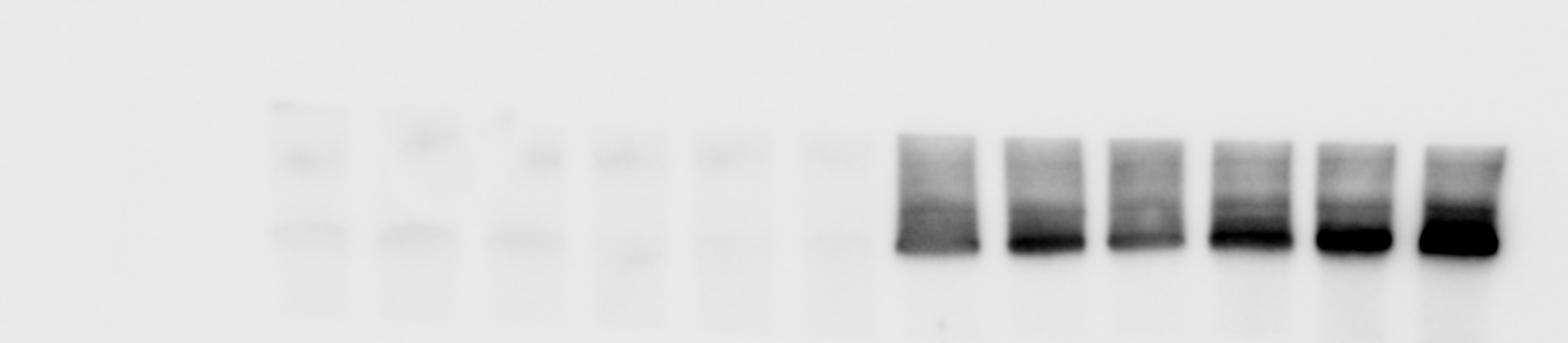

Supplement: Figure 8—source data 7. [file elife-90724-fig8-data7.zip › Slow MyHC.tif]

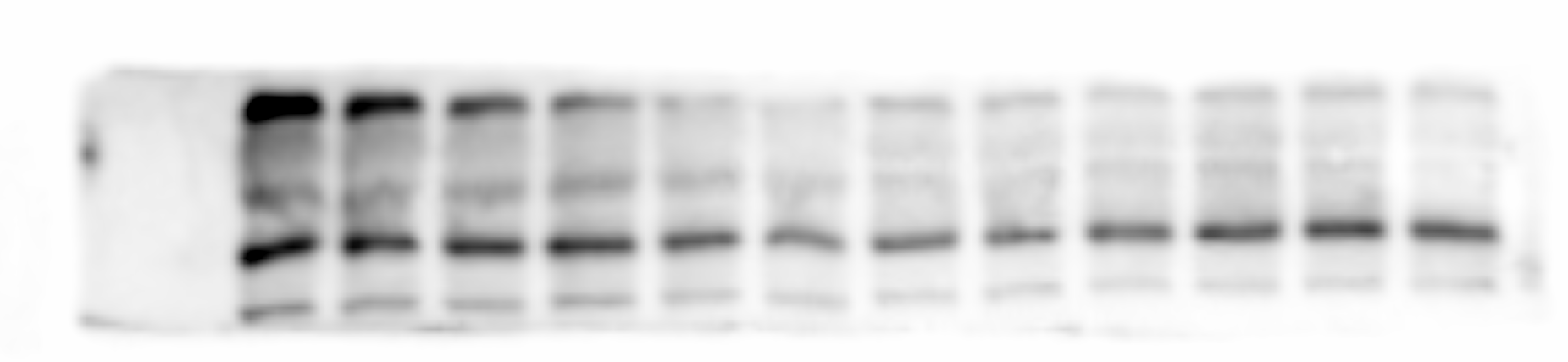

Supplement: Figure 8—source data 7. [file elife-90724-fig8-data7.zip › a┬-Actin.tif]

**Figure 8E**

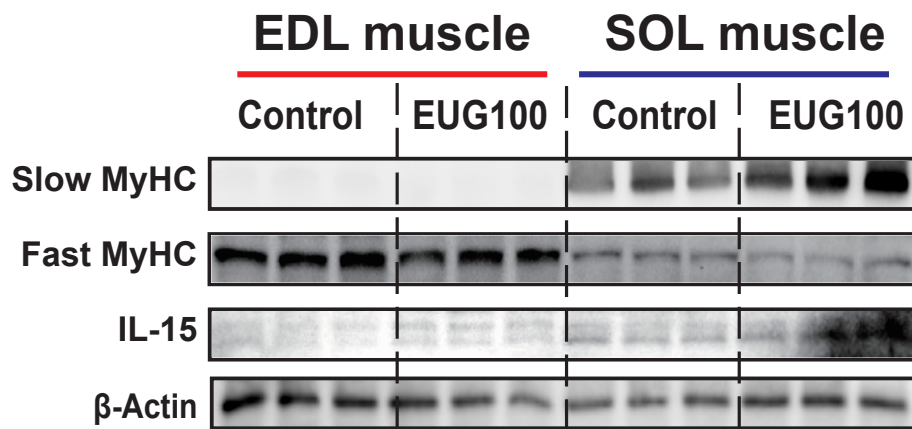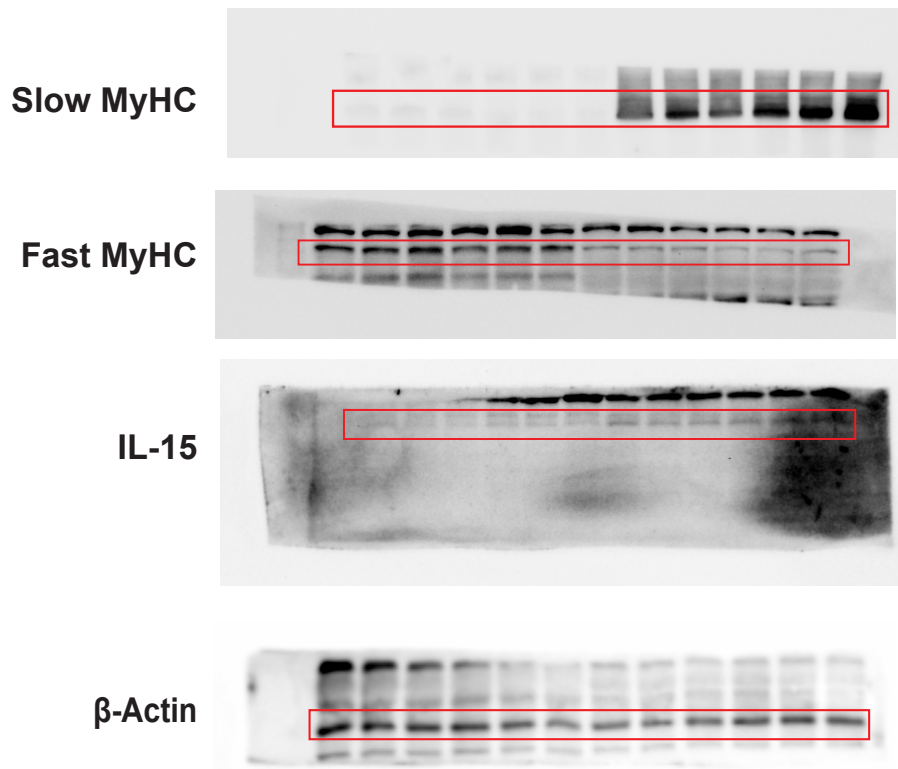

Supplement: Figure 8—source data 8. [file elife-90724-fig8-data8.pdf]

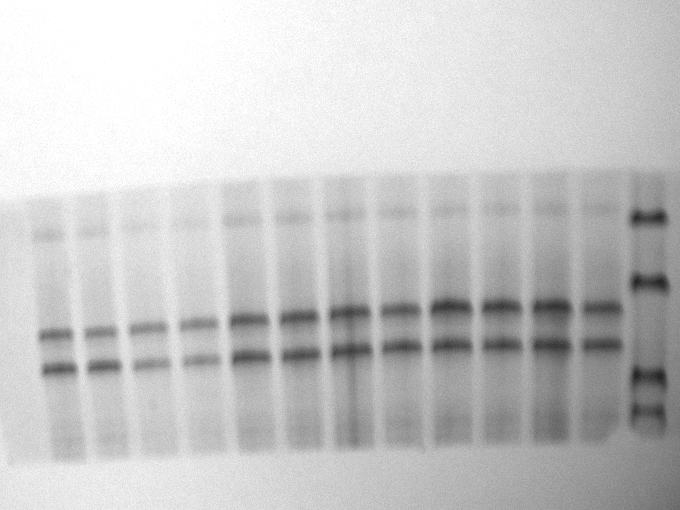

Supplement: Figure 9—source data 2. [file elife-90724-fig9-data2.zip › Coomassie staining-Blot.jpg]

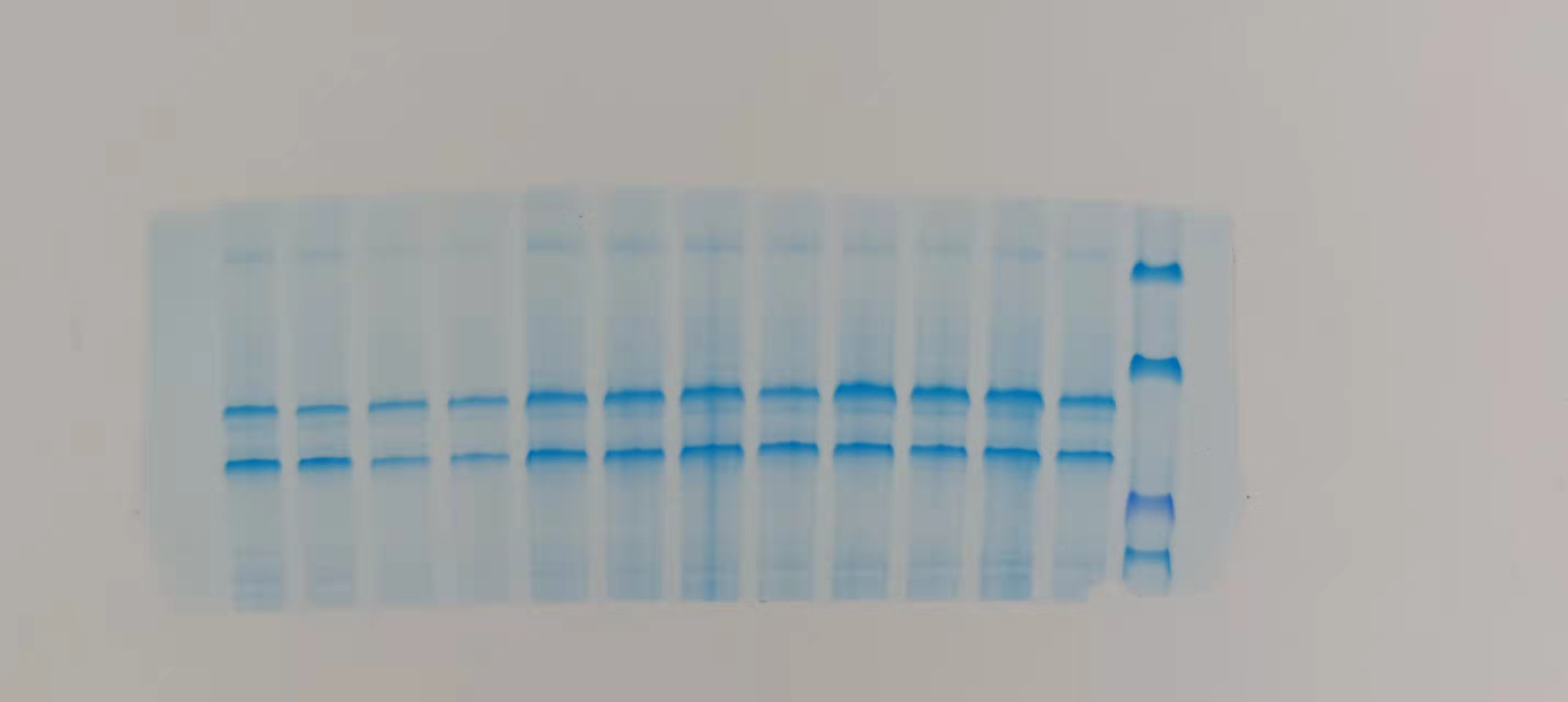

Supplement: Figure 9—source data 2. [file elife-90724-fig9-data2.zip › Coomassie staining.jpg]

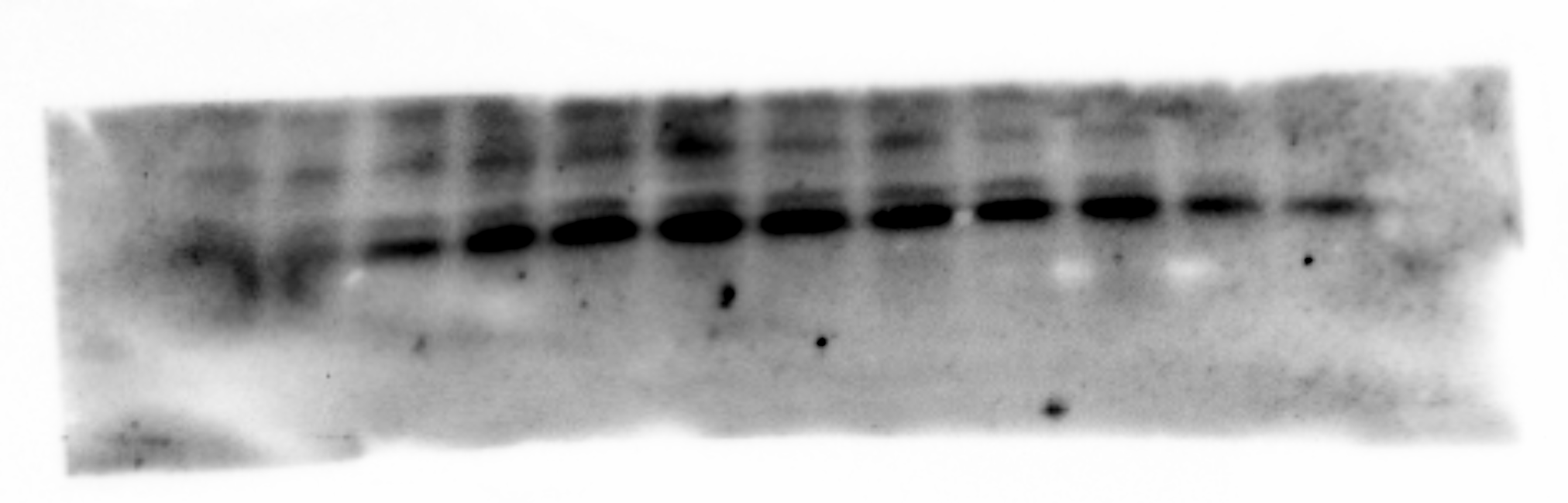

Supplement: Figure 9—source data 2. [file elife-90724-fig9-data2.zip › IL-15.tif]

Figure 9B

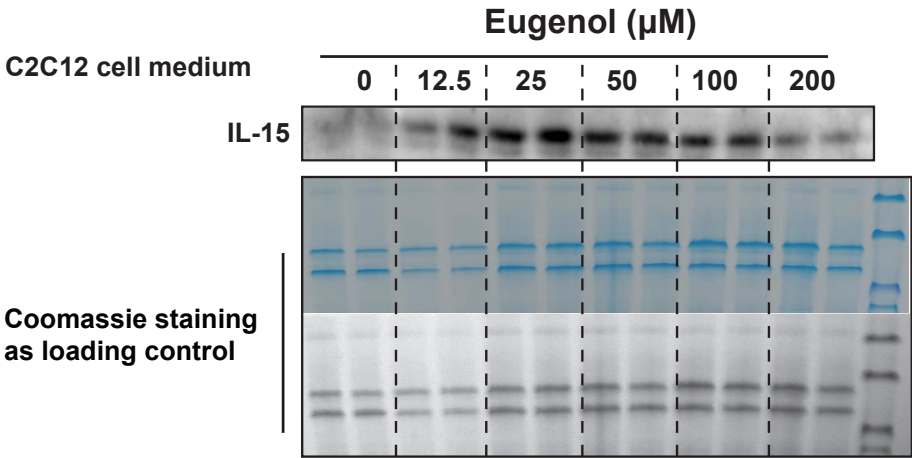

IL-15

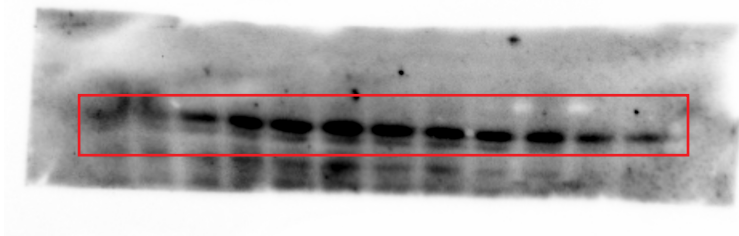

Coomassie staining

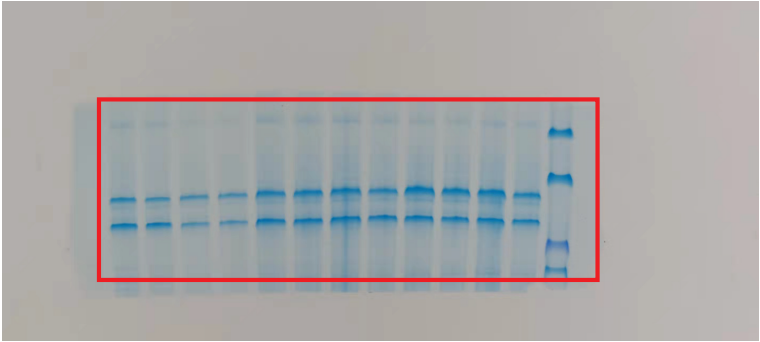

Coomassie staining-Blot

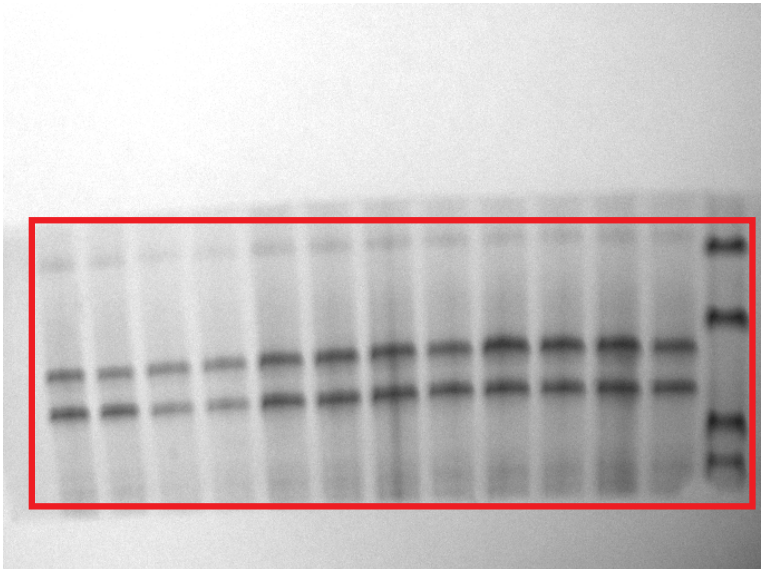

Supplement: Figure 9—source data 3. [file elife-90724-fig9-data3.pdf]

Figure 9D

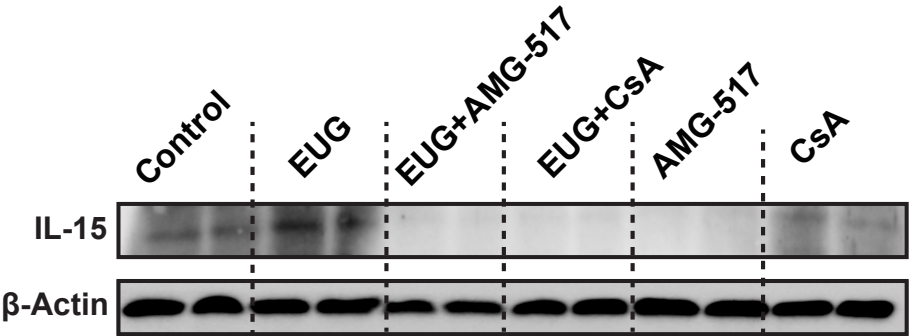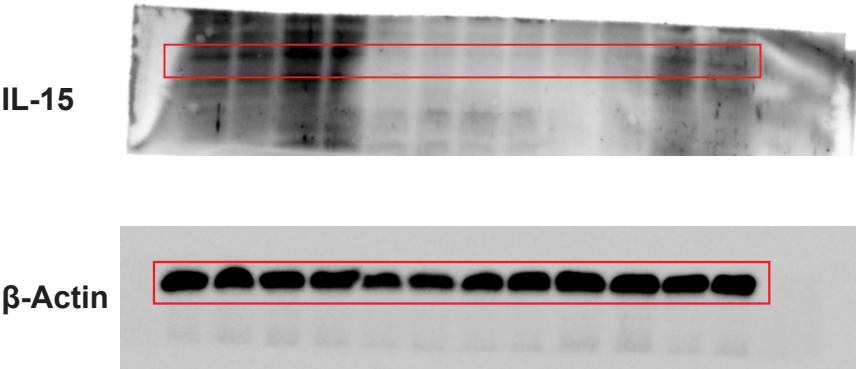

Supplement: Figure 9—source data 5. [file elife-90724-fig9-data5.pdf]

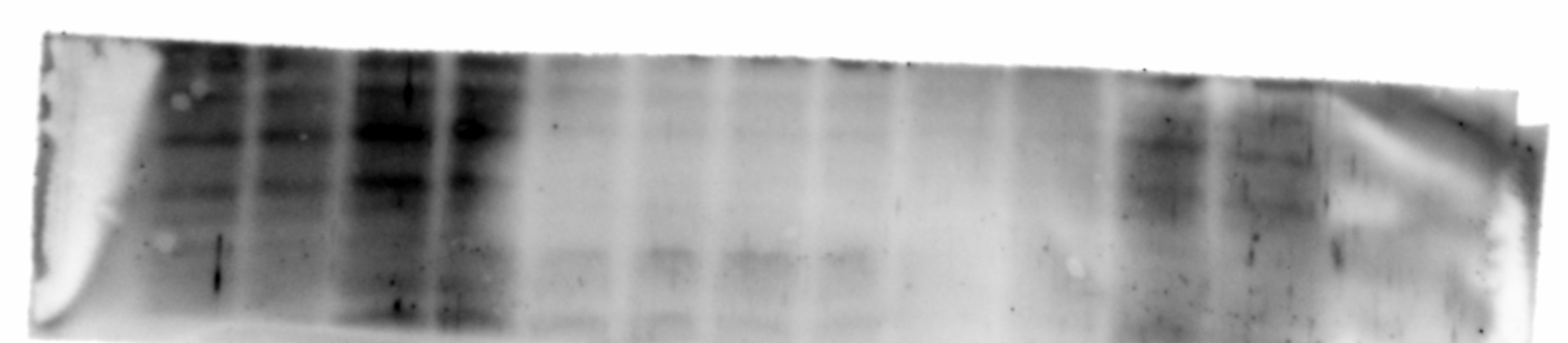

Supplement: Figure 9—source data 6. [file elife-90724-fig9-data6.zip › IL-15.tif]

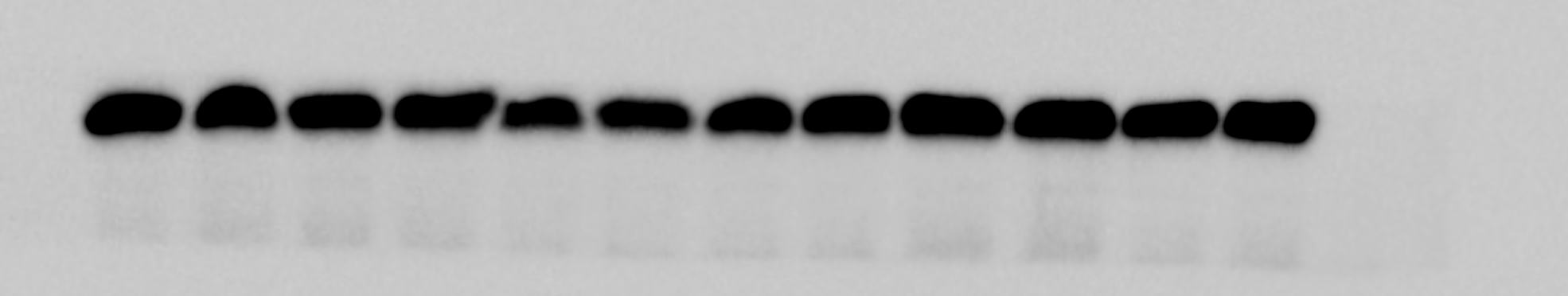

Supplement: Figure 9—source data 6. [file elife-90724-fig9-data6.zip › a┬-Actin.tif]
